# Tumor immune dysfunction and exclusion subtypes in bladder cancer and pan-cancer: a novel molecular subtyping strategy and immunotherapeutic prediction model

**DOI:** 10.1186/s12967-024-05186-8

**Published:** 2024-04-17

**Authors:** Kun Zheng, Youlong Hai, Hongqi Chen, Yukun Zhang, Xiaoyong Hu, Kai Ni

**Affiliations:** 1 Department of Urology, Shanghai Sixth People’s Hospital Affiliated to Shanghai Jiao Tong University School of Medicine, Shanghai, 200233 China; 2 Department of Urology, The Affiliated Jiangsu Shengze Hospital of Nanjing Medical University, Suzhou, 215200 Jiangsu China; 3 Beijing University of Chinese Medicine East Hospital, Zaozhuang Hospital, Zaozhuang, 277000 Shandong China

**Keywords:** Bladder cancer, Immune dysfunction and exclusion, Molecular subtyping, Immunotherapy, RNA-sequencing, Pan‑cancer

## Abstract

**Background:**

Molecular subtyping is expected to enable precise treatment. However, reliable subtyping strategies for clinical application remains defective and controversial. Given the significance of tumor immune dysfunction and exclusion (TIDE), we aimed to develop a novel TIDE-based subtyping strategy to guide personalized immunotherapy in the bladder cancer (BC).

**Methods:**

Transcriptome data of BC was used to evaluate the heterogeneity and the status of TIDE patterns. Subsequently, consensus clustering was applied to classify BC patients based on TIDE marker-genes. Patients’ clinicopathological, molecular features and signaling pathways of the different TIDE subtypes were well characterized. We also utilize the deconvolution algorithms to analyze the tumor microenvironment, and further explore the sensitivity and mechanisms of each subtype to immunotherapy. Furthermore, BC patient clinical information, real-world BC samples and urine samples were collected for the validation of our findings, which were used for RNA-seq analysis, H&E staining, immunohistochemistry and immunofluorescence staining, and enzyme-linked immunosorbent assay. Finally, we also explored the conservation of our novel TIDE subtypes in pan-cancers.

**Results:**

We identified 69 TIDE biomarker genes and classified BC samples into three subtypes using consensus clustering. Subtype I showed the lowest TIDE status and malignancy with the best prognosis and highest sensitivity to immune checkpoint blockade (ICB) treatment, which was enriched of metabolic related signaling pathways. Subtype III represented the highest TIDE status and malignancy with the poorest prognosis and resistance to ICB treatment, resulting from its inhibitory immune microenvironment and T cell terminal exhaustion. Subtype II was in a transitional state with intermediate TIDE level, malignancy, and prognosis. We further confirmed the existence and characteristics of our novel TIDE subtypes using real-world BC samples and collected patient clinical data. This subtyping method was proved to be more efficient than previous known methods in identifying non-responders to immunotherapy. We also propose that combining our TIDE subtypes with known biomarkers can potentially improve the sensitivity and specificity of these biomarkers. Moreover, besides guiding ICB treatment, this classification approach can assist in selecting the frontline or recommended drugs. Finally, we confirmed that the TIDE subtypes are conserved across the pan-tumors.

**Conclusions:**

Our novel TIDE-based subtyping method can serve as a powerful clinical tool for BC and pan-cancer patients, and potentially guiding personalized therapy decisions for selecting potential beneficiaries and excluding resistant patients of ICB therapy.

**Supplementary Information:**

The online version contains supplementary material available at 10.1186/s12967-024-05186-8.

## Introduction

Bladder cancer (BC) is a prevalent malignancy of the urinary system, ranking ninth in incidence and thirteenth in mortality among all cancers [1, 2]. Although early-stage BC is typically treated with surgery, high rates of postoperative recurrence often require multimodal interventions [3–5]. For advanced or metastatic cases, systemic treatments are the current research hotspots, especially for immunotherapy [6]. Despite significant progress in recent years, 5-year recurrence-free survival rate still falls below 43% [7, 8]. Currently, personalized precision therapy is gradually becoming the mainstream treatment. Immune checkpoint blockade (ICB) can elicit long-lasting responses in partial metastatic cancer patients [9]. For locally advanced or metastatic BC patients, who are refractory to platinum-based therapy, ICB has been regarded as a first-line or second-line treatment option [10]. Despite its great potential, only a small percentage of patients benefit from ICB (< 30%) [11, 12]. The exact mechanisms and predictive factors that affect ICB efficacy remain unclear. Previous studies have identified some factors associated with ICB response, such as tumor immune microenvironment (TIME) patterns [13–16], tumor mutational burden (TMB) and neoantigen load [17, 18], and microsatellite instability (MSI) [19]. These findings are essential for understanding the factors related to ICB response and developing predictive biomarkers. The current key obstacle for accurate therapy prediction is the search of ICB response biomarkers and resistance regulators [20, 21]. Tumor molecular subtyping is a research trend for precision diagnosis and treatment, but currently limited subtyping method can accurately guide ICB therapy in BC patients [22, 23].

Within the tumor microenvironment (TME), tumor cells occupy specialized niches where they interact extensively with various factors, such as stromal and immune cells. These interactions have a significant impact on tumor initiation, progression, metastasis, and therapy response [24–26]. Studies have found that some cancer cell subsets can affect patients’ responses to immunotherapy, which are closely in contact with cancer-associated fibroblasts (CAFs) and CD8^+^ T cells (CD8Ts) [27, 28]. Moreover, inhibitory cells, cytokines and metabolites that generate an immunosuppressive environment within the TME can reduce the activation and function of cytotoxic T-cells (CTLs), which will result in the tumor immune dysfunction (TID) or the exclusion of T-cells from the tumor (TIE) [25, 29, 30]. These two tumor-immune escape mechanisms will undermine tumor response to ICB therapy [20]. Liu et al. used tumor expression profiling data of melanoma and non-small cell lung cancer (NSCLC) to score these mechanisms and developed a computational framework called the Tumor Immune Dysfunction and Exclusion (TIDE) algorithm [20]. However, its effectiveness in BC, and further in pan-cancers, requires authoritative validation.

In this study, transcriptome analysis was conducted in the BC patients to assess TIDE status and identify specific biomarkers. Based on the identified marker-genes, a novel TIDE subtyping strategy was constructed which can classify BC patients into three subtypes with different clinicopathological and molecular features, prognoses, functional annotations, TME and therapy responses. This TIDE-based subtyping was also proven to accurately predict ICB treatment and chemotherapy outcomes in the BC patients. Compared to existing methods of BC molecular subtyping and previously published predictive biomarkers for BC immunotherapy, our innovative TIDE-based subtyping strategy not only demonstrates a close correlation with clinicopathological and molecular characteristics in BC patients but also offers improved accuracy and reliability in identifying responders and non-responders to ICB treatment. This approach has the potential to provide precision guidance for enhancing the effectiveness of clinical immunotherapy in BC patients. Furthermore, we validated the existence of the TIDE subtypes and their associations with ICB responses in five pan-cancer cohorts, which potentially indicates the consistency of our TIDE method across pan-tumors. Overall, we aimed to employ transcriptome data to evaluate TIDE status and develop an accurate TIDE-based decision-making tool in the BC and pan-cancer patients, which can facilitate precise treatment for tumors and gains valuable insights into the molecular mechanisms underlying tumor immune dysfunction and exclusion.

## Results

### Correlations of TIDE status with the clinicopathological and molecular features in the BC patients

#### Associations of TIDE scores with clinicopathological and molecular features

**Fig. 1:**
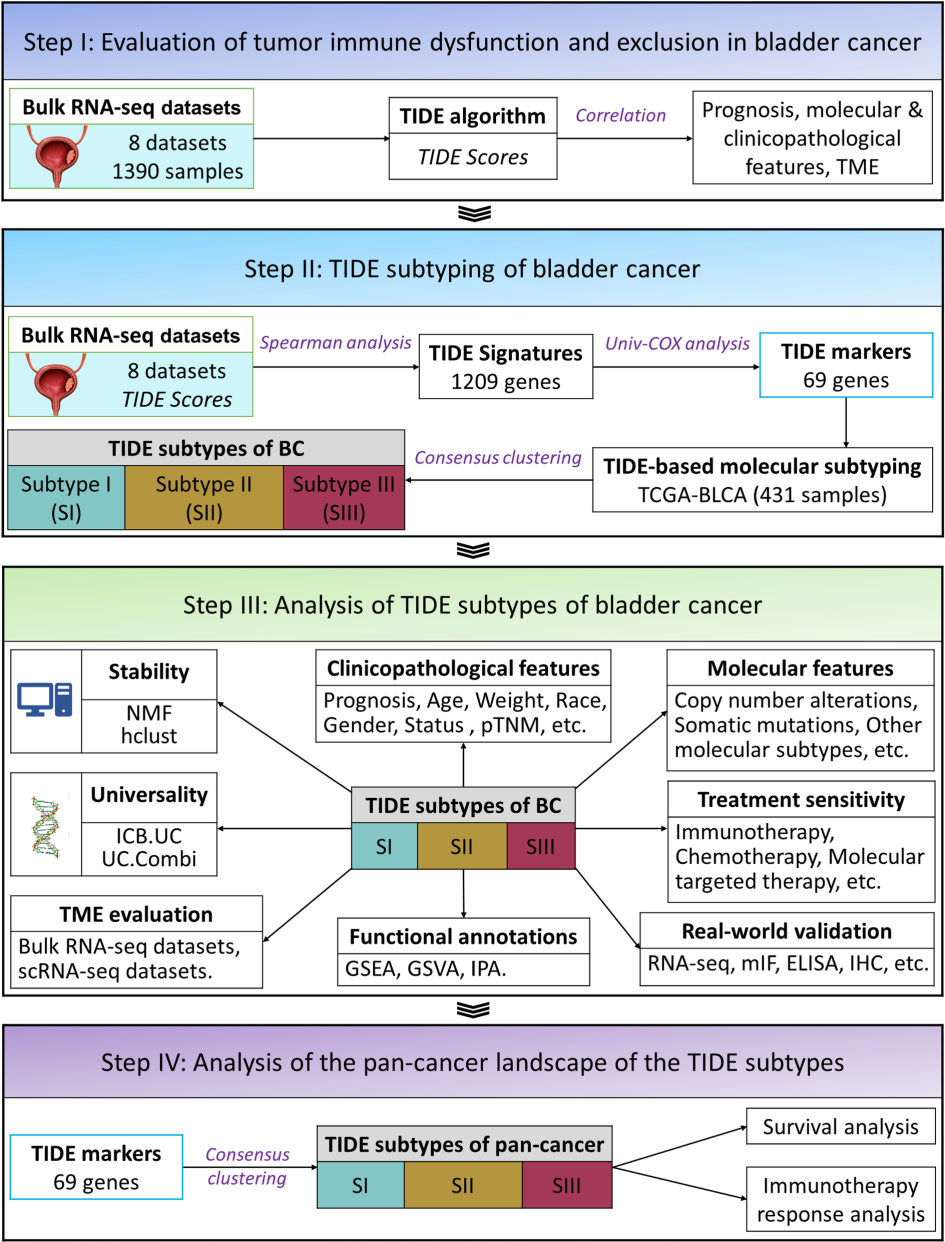
The overall design of the current study

**Fig. 2:**
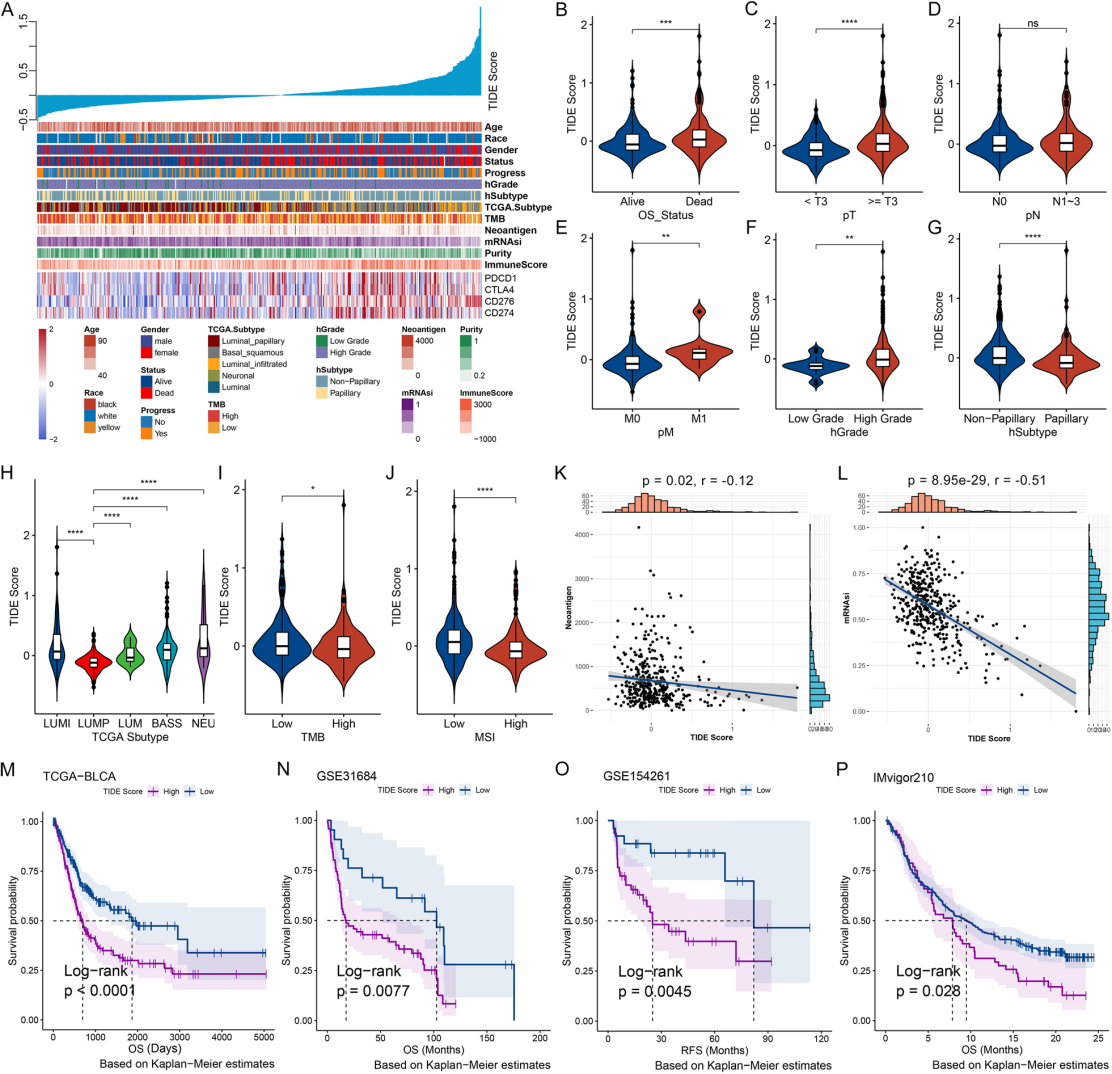
Correlations of TIDE status with clinicopathological and molecular features in the BC patients. **A** Associations of TIDE scores with clinicopathological and molecular features in BC patients. Columns represented samples ranked by TIDE scores from low to high (top row), and rows represent clinicopathological and molecular features associated with TIDE scores. **B**–**J**) Comparisons of TIDE scores with subgroups of survival status (OS_Status) (**B**), pathological TNM stage (pTNM) (**C**–**E**), histological grade (hGrage) (**F**), histological subtype (hSubtype) (**G**), TCGA mRNA subtype (**H**), tumor mutation load (TMB) (**I**) and microsatellite instability (MSI) (**J**). LUMI, Luminal-infiltrated; LUMP, Luminal-papillary; LUM, Luminal; BASS, Basal-squamous; NEU, Neuronal. **K**, **L** Correlations of TIDE scores with neoantigen load (**K**) and stemness index (mRNAsi) (**L**) in BC patients. **M**–**P** Kaplan–Meier (K–M) analysis demonstrated a correlation of the TIDE scores with the prognosis of BC patients from TCGA-BLCA (**M**), GSE31684 (**N**), GSE154261 (**O**) and IMvigor210 (**P**) datasets. OS, overall survival; RFS, recurrence-free survival. Dashed line: median survival time. Color range: 95% confidence interval (CI). *p < 0.05, **p < 0.01, ***p < 0.001, ****p < 0.0001; ns, no significance

The workflow of our study is illustrated in Fig. 1, Additional file 1: Figure S1 and Methods S1. We utilized the TIDE algorithm [20] to evaluate TIDE status and calculate TIDE scores based on bulk RNA sequencing (RNA-seq) data. The samples were sorted from low to high to explore how TIDE scores were related to the clinicopathological and molecular features (Fig. 2A). The results showed that younger patients, Asian ethnicity, and male gender had significantly lower TIDE scores (Additional file 1: Figure S2A). TIDE scores were dramatically higher in those who died of BC, but unrelated to tumor progression (Fig. 2B, Additional file 1: Figure S2B). Additionally, patients with advanced pathological T stage (T3 and T4), distant metastasis and high histological grade had higher TIDE scores, but not lymph node metastasis (Fig. 2C–F). Finally, TIDE scores of Papillary and Luminal papillary subtypes were the lowest among all histological subtypes and TCGA subtypes (Fig. 2G, H).

Based on the somatic mutation data, we revealed that TIDE scores showed obviously negative correlations with TMB, MSI, neoantigen load, and stemness scores (mRNAsi) (Fig. 2I–L). For common biomarker mutation events, there were significant differences of TIDE scores between KDM6A, FGFR3, and TAF11 mutant patients and wild-type patients (Additional file 1: Figure S2C).

Kaplan–Meier (K–M) analysis revealed significant associations of higher TIDE group with poorer overall survival (OS, Fig. 2M), progression-free interval (PFI), and disease-specific survival (DSS) (Additional file 1: Figure S2D). Additional five BC datasets confirmed these findings (Fig. 2N–P, Additional file 1: Figure S2E). Therefore, based on the survival analysis results, tumor immune dysfunction and exclusion levels are probably two risk factors for BC.

#### Correlation between TIDE status and TIME in the BC patients

To determine the immune patterns of 431 samples of the Cancer Genome Atlas—Bladder Urothelial Carcinoma (TCGA_BLCA), we employed the Single Sample Gene Set Enrichment Analysis (ssGSEA) algorithm [31] to quantify scores for the 54 immune signatures published by Charoentong et al. [32] and Şenbabaoğlu et al. [33] The samples were divided into two groups: “High immune infiltration” (High-immu; n = 230, 53.4%) and “Low immune infiltration” (Low-immu; n = 201, 46.6%) (Additional file 1: Figure S3A). Using the ESTIMATE algorithm [34] to evaluate TME, the immune and stromal scores of High-immu group were significantly higher than the scores of Low-immu group (Additional file 1: Figure S3B). DECEPTICON was applied to evaluate the immunocyte abundance. The results indicated that all types of immunocytes were significantly enriched in the High-immu group (Additional file 1: Figure S3C), and a significant association was observed between the High-immu group and high TIDE scores, and vice versa (Additional file 1: Figure S3D). Moreover, TIDE scores were positively correlated with Immune and Stromal scores, and negatively correlated with tumor purity and DNA fraction (Additional file 1: Figure S3E). The myeloid cell infiltration was positively correlated with TIDE scores using Mantel’s test (Additional file 1: Figure S3E).

### Identification of three TIDE subtypes with significant differences in prognosis

#### Identification of TIDE marker genes

**Fig. 3:**
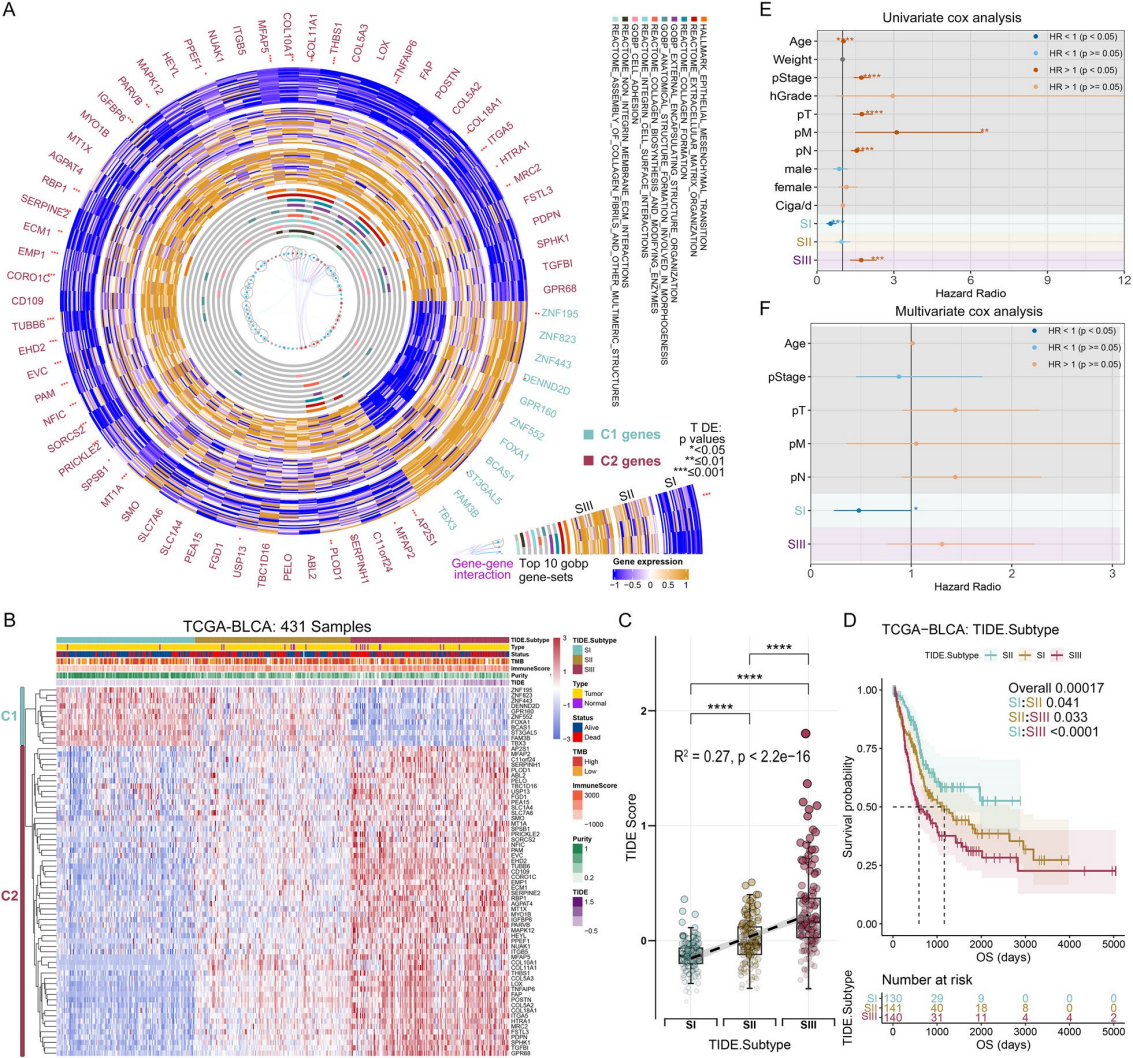
Identification of three TIDE-based subtypes of BC based on the TIDE marker genes. **A** CircosPlot shows the expression levels in TCGA-BLCA, signaling pathways and protein–protein interaction (PPI) networks of 69 TIDE marker genes. These genes are mainly divided into two clusters: C1 consists of 11 genes (cyan) and C2 comprises 58 genes (red). **B** Consensus clustering based on the expression of 69 TIDE marker genes classified patients of TCGA-BLCA into three subtypes: Subtype I (SI), Subtype II (SII), and Subtype III (SIII). C1 genes are labelled in cyan, and C2 are labelled in red. **C** Levels and trends of TIDE scores among three TIDE subtypes. **D** OS of TCGA-BLCA is significantly different among three TIDE subtypes. **E**, **F** Univariate (**E**) and multivariate (**F**) Cox regression analysis of the three TIDE subtypes with clinical and molecular characteristics. *p < 0.05, ***p < 0.001, ****p < 0.0001

Due to the correlation with TIDE scores and the prognosis of BC patients, we hypothesized that certain markers reflecting TIDE status could be used for molecular subtyping. Eight BC bulk RNA-seq datasets [29, 35–39] were applied to develop TIDE marker-genes. The detailed process is shown in Additional file 1: Figure S4A and Methods S1. Sixty-nine genes representing significant association with OS were selected (Fig. 3A, Additional file 2: Data S1). These genes were classified into two clusters (Additional file 1: Figure S4B). C1 comprises 11 genes that have no interaction with each other, while C2 includes 58 genes that were significantly enriched in signal pathways related to collagen and extracellular matrix metabolism, cell adhesion and integrin-mediated signal transduction, and epithelial-mesenchymal transition (Fig. 3A, Additional file 1: Figure S4C). In addition, Venn analysis showed that 43.5% (30/69) of TIDE marker genes also belonged to TIME signature genes (Additional file 1: Figure S4D, Additional file 2: Data S2). These shared genes may play a critical role in shaping both TIDE status and the tumor immune microenvironment.

#### Identification of three TIDE subtypes

Unsupervised consensus clustering [40] was used to identify TIDE subtypes based on the expression profiling of 69 marker-genes. Three clusters were well optimized determined with the help of consensus heatmap, cumulative distribution function (CDF) curves and proportion of ambiguous clustering algorithm (PAC) (35) (Additional file 1: Figure S5A, Fig. 3B). Subtype I (SI) consisted of 132 samples (30.6%) with high-expression of C1 genes and low-expression of C2 genes, subtype II (SII) comprised 148 samples (34.3%) with moderate-expression of both C1 and C2 genes, and subtype III (SIII) contained 151 samples (35.0%) with low-expression of C1 genes and high-expression of C2 genes (Fig. 3B). The TIDE, Dysfunction and Exclusion scores were gradually increased from SI to SIII (Fig. 3C, Additional file 1: Figure S5B).

#### Differences of prognosis among the TIDE subtypes in the BC patients

Based on K-M analysis, SIII represented the poorest prognosis, whereas SI owned the best prognosis, and SII showed an intermediate prognosis (Fig. 3D, Additional file 1: Figure S5C). By univariate Cox analysis, SI and SIII were protective and risk factors for OS, respectively (Fig. 3E), and SI was an independent protective factor for OS by multivariate Cox analysis (Fig. 3F).

#### Stability and universality of TIDE subtyping strategy

Non-negative matrix factorization (NMF) [41] and unsupervised hierarchical clustering were used to classify TCGA-BLCA patients (Additional file 1: Figure S5D–G). The results were comparable to those obtained by using the consensus clustering. Therefore, TIDE subtyping strategy is robust across different algorithms. To further validate its universality, our novel TIDE subtyping strategy was also utilized in the other two integrated bulk RNA-seq cohorts: the UC.Combi cohort (n = 960, five independent datasets [35–39]) and the ICB.UC cohort (n = 384, BC samples sequenced before ICB therapy, three datasets [29, 42]). Both cohorts were consistently classified into three subtypes (Additional file 1: Figure S5H, I) with significant TIDE scores and prognosis differences (Additional file 1: Figure S5J–M). Therefore, these results indicate a good universality of the TIDE subtyping method. To determine whether publication bias impacts the results, we conducted the Egger’s test for the BC datasets [43]. The result revealed no publication bias (p = 0.7689), and funnel plot did not reveal any significant asymmetry (Additional file 1: Figure S13A). Moreover, there was no indications of studies identified with the trim-and-fill analysis (no trimming performed, no new studies added and data unchanged) (Additional file 1: Figure S13B).

### Distinct clinical features, mutational events, and functional annotations among the TIDE subtypes

#### Differences of clinicopathological features among the TIDE subtypes

**Fig. 4:**
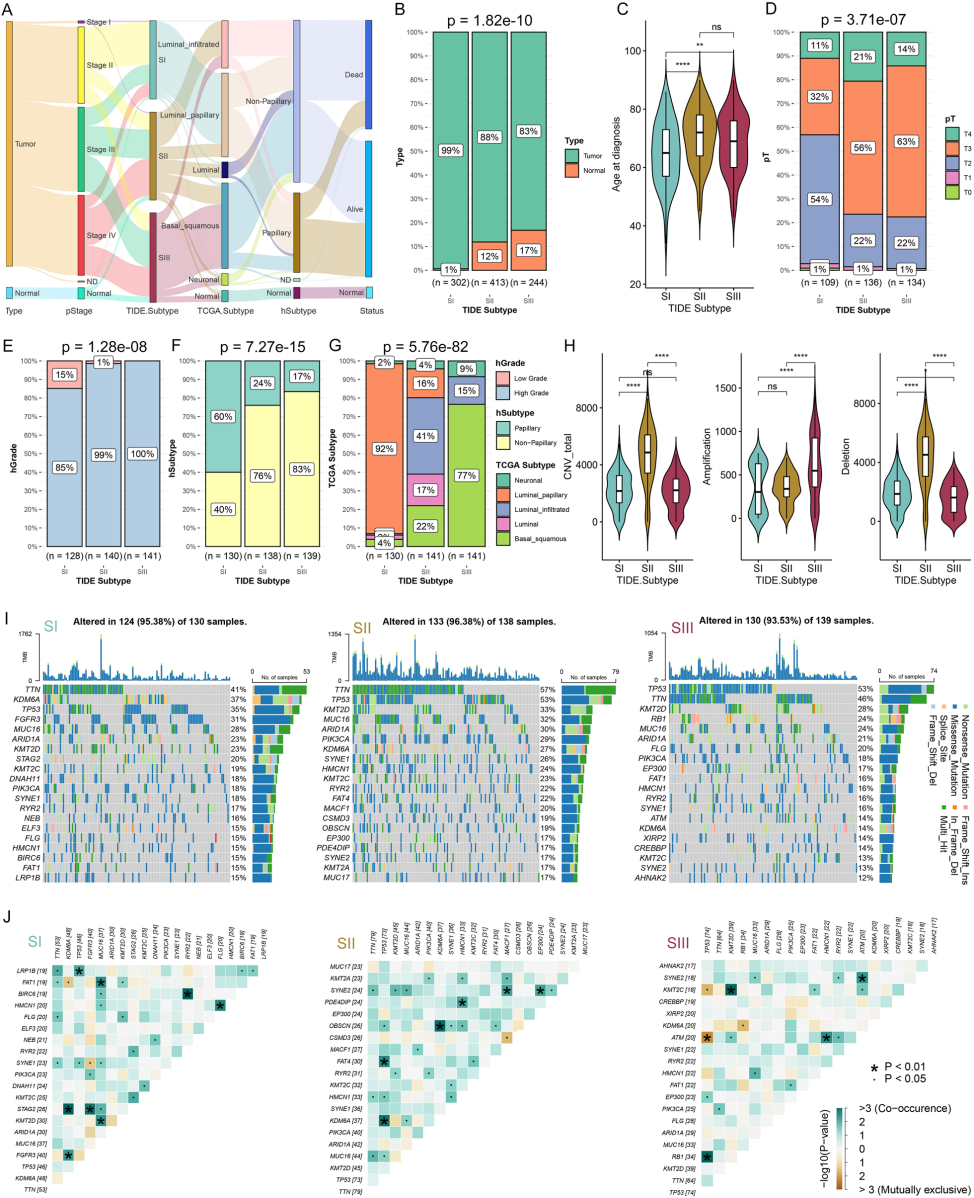
Comparisons of clinicopathological and molecular features among three TIDE subtypes in the BC patients. **A** Sankey diagram showing sample flows for TIDE subtype, sample type, pathological stage (pStage), TCGA Subtype, hSubtype, and survival status. **B** The proportion of sample type among the TIDE subtypes of UC.Combi cohort. **C** Comparisons of age at diagnosis among TIDE subtypes. **D**–**G** The proportion of pathological T stage (pT) (**D**), hGrade (**E**), hSubtype (**F**) and TCGA Subtype (**G**) among the three TIDE subtypes. **H** Comparisons of total copy number variations (CNV), amplifications and deletions among three TIDE subtypes. **I** Oncoplots showing the top 20 mutated genes in SI (left), SII (middle), and SIII (right). **J** Heatmap showing mutually exclusive or co-occurrence events among top mutated genes in SI (left), SII (middle), and SIII (right). *p < 0.05, **p < 0.01, ***p < 0.001, ****p < 0.0001; ns, no significance

The proportion of cancer adjacent samples were gradually increased from SI to SIII (Figs. 4A, B, Additional file 1: Figure S6A). TIDE subtypes have obvious differences in diagnostic age (Fig. 4C), race and gender, but not in weight and daily smoking (Additional file 1: Figure S6A). Regarding histopathology, SI had the lowest proportion of pT3 and pT4, high-grade (Fig. 4D, E) and lymph node-positive patients (Additional file 1: Figure S6B). We also observed a decreased proportion of patients with the Papillary histological subtype from SI to SIII (Fig. 4F), and increased proportions of overall death, BC-specific death and progression (Additional file 1: Figure S6C).

#### Differences of molecular features among the TIDE subtypes

We further analyzed the compositions of different molecular subtypes in the TIDE subtypes and discovered obvious distribution differences, indicating a correlation and similarity between the known molecular subtypes and the new TIDE-based subtyping system (Fig. 4G, Additional file 1: Figure S6D). For example, the Luminal papillary subtype accounted for 92% in SI, and the Basal squamous subtype constituted 77% in SIII (Fig. 4G). The SCNA and somatic mutation analysis showed that SII owned the highest copy number variation (CNV) burden (Fig. 4H). However, SIII and SII displayed the highest amplification and deletion burdens, respectively. SII exhibited the highest level of TMB, as reflected by the total number of single nucleotide polymorphisms (Additional file 1: Figure S6E). Each subtype showed the specific top mutated genes, with significant differences of mutation rates in the shared genes among the subtypes (Fig. 4I). We examined gene co-mutation or mutual exclusivity patterns for each subtype (Fig. 4J), which may affect BC subtyping, treatment, and prognosis. For instance, SIII carried co-mutated SYNE2 and ATM, two DNA damage repair genes. This rare co-mutation may increase DNA damage, tumor progression and metastasis, and alter tumor response to therapies [44]. Additionally, we investigated the mutation status of common BC biomarkers among the TIDE subtypes. TP53, PIK3CA, RB1, KDM6A, FGFR3ELF3, KMT2A, NFE2L2, FAT1 and SSH3 differed significantly in mutation proportions among subtypes (Additional file 1: Figure S6F).

#### Validation of the TIDE subtypes using the collected real-world BC samples

**Fig. 5:**
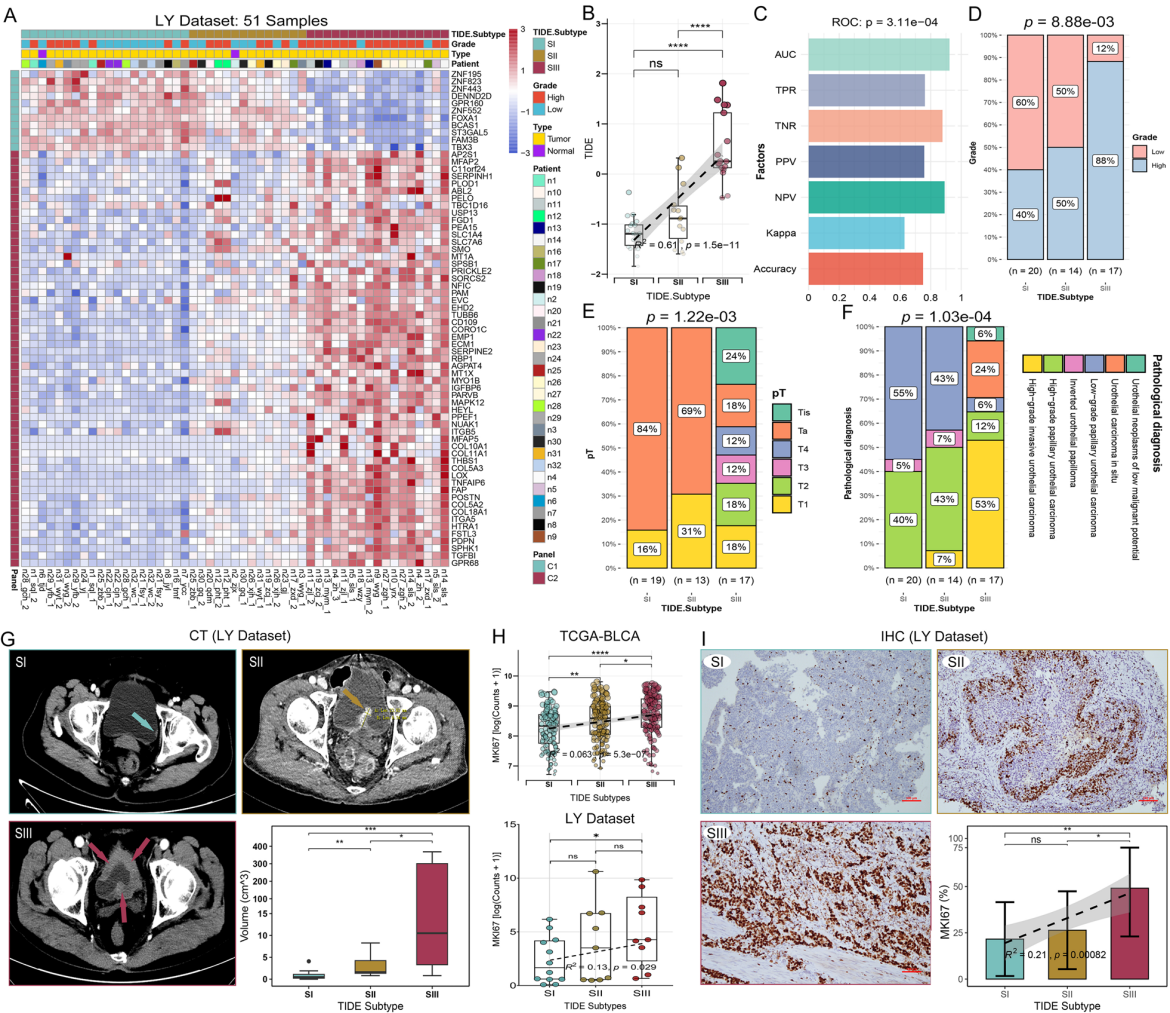
Validation of the TIDE subtypes using the collected real-world BC samples. **A** Consensus clustering by the TIDE marker genes can distinguish the real-world BC samples into three TIDE subtypes. **B** Levels and trends of TIDE scores among the TIDE subtypes. **C** Multi-class ROC curve analysis was used to evaluate the subtyping consistency of repeated samples. **D**–**F** The proportion of the tumor grade (**D**), pT (**E**) and pathological diagnosis (**F**) among the TIDE subtypes. **G** Typical CT tomographic images of bladder tumors ( →) and tumor volumes based on CT tomography in three TIDE subtypes. **H** Transcription levels of representative BC proliferation marker MKI67 in TCGA-BLCA and LY Dataset among three TIDE subtypes. **I** MKi67 IHC images from the collected BC samples and analysis of MKi67 positive cells among the TIDE subtypes. Scale bar, 100 μm. *p < 0.05, **p < 0.01, ***p < 0.001. ns, no significance

Fifty-one BC samples were collected for RNA sequencing (LY Dataset) to confirm the TIDE subtypes (Fig. 5A, B). The detailed clinical information is shown in Additional file 1: Table S2. The area under the receiver operating characteristic curve (AUC) of repeated sample subtyping consistency is as high as 0.92, and the accuracy is 75% (Fig. 5C). Subtyping analysis indicated that the proportion of high-grade BC was increased from SI to SIII (Fig. 5D). Among the 51 samples, the highest proportion of Ta was in SI and the highest proportion of T1 was in SII. T2–T4, and Tis stages belonged to SIII (Fig. 5E). In terms of pathological diagnosis, SI possessed the highest proportion of papillary urothelial carcinoma, while SIII was consisted of the highest proportion of invasive urothelial carcinoma and urothelial carcinoma in situ (Fig. 5F). We also found significant differences in tumor volume among the subtypes based on CT imaging data (Fig. 5G). Tumors belonging to SI were generally smaller, while those belonging to SIII were often larger or even infiltrated the entire bladder (Fig. 5G). Finally, RNA-seq analysis based on TCGA-BLCA and real-world samples (LY Dataset), and immunohistochemistry (IHC) consistently showed that MKI67 (Ki67) expression level were highest in SIII (Fig. 5H, I), indicating the high proliferative ability of this subtype. These results consistently demonstrate that the SIII subtype has the highest malignancy and is closely associated with urothelial carcinoma in situ.

#### Distinct signaling pathways and functional annotations among the three TIDE subtypes

Gene Set Variation Analysis (GSVA), Gene Set Enrichment Analysis (GSEA), and Ingenuity Pathway Analysis (IPA) were conducted to investigate the functional annotations and potential mechanisms associated with TIDE subtypes. GSEA showed SI had enriched signaling pathways related to lipid and xenobiotic metabolism (Additional file 1: Figure S7A). In contrast, SII displayed enriched pathways related to complement activation, humoral immunity, scavenger receptor and B cell receptor (Additional file 1: Figure S7B). SIII showed enriched pathways related to various cellular processes, including migration, activation, development, proliferation, inflammation and immune regulation (Additional file 1: Figure S7C). These results were consistent with the observation from GSVA (Additional file 1: Figure S7D). IPA analysis of canonical signaling pathways were used to reveal the status of activation or inhibition. Our results showed that SI activated biosynthesis and xenobiotic metabolism pathways, but suppressed cancer, stress, cytokine, immune and growth pathways (Additional file 1: Figure S7E). SIII displayed opposite pathway activity to SI (Additional file 1: Figure S7I). SII was a transitional state with mixed pathway signals (Additional file 1: Figure S7G). The graphical summary suggested that SI suppressed immune and inflammatory factors, while SIII activated them (Additional file 1: Figure S7F, J, K). Lastly, consistent with GSVA and GSEA, SI activated lipid metabolism pathways, but SIII suppressed them (Additional file 1: Figure S7L).

### TME patterns of three TIDE subtypes

**Fig. 6:**
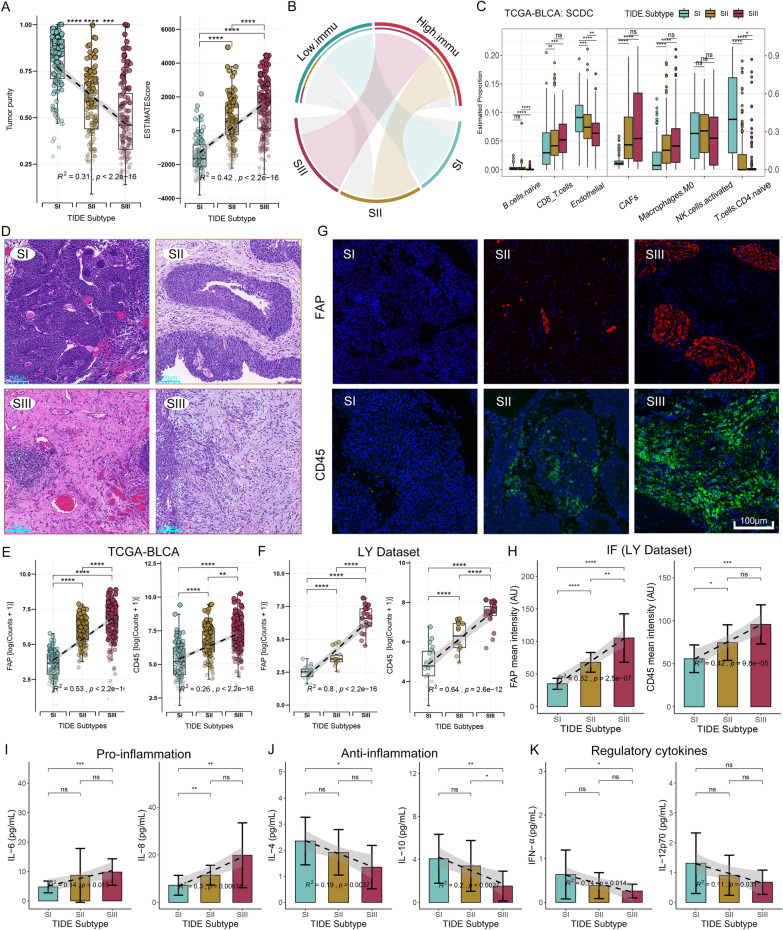
Characterization of TME patterns in the TIDE subtypes. **A** Differences of tumor purity and ESTIMATEScore among the TIDE subtypes. **B** Hypergeometric tests revealed an association between TME patterns and TIDE subtypes. Gray lines represent no significance. **C** Boxplots showing comparisons of stromal cells abundance among the three TIDE subtypes of TCGA-BLCA. Cell proportions are assessed by the SCDC algorithm. **D** H&E histological images of the three subtypes from the real-world BC samples. Scale bar, 100 μm. **E**, **F** Transcription levels of representative fibrosis marker FAP and immune infiltration marker CD45 (PTPRC) in the TCGA-BLCA (**E**) and LY Dataset (**F**) among the TIDE subtypes. **G** IF images of FAP and CD45 (PTPRC) in three TIDE subtypes from the real-world BC samples. Scale bar, 100 μm. **H** Analysis of mean fluorescence intensity (AU) of FAP and CD45 (PTPRC) in the TIDE subtypes. **I**–**K** Comparisons of the concentration of pro-inflammatory (IL-6, IL-8) **(I)**, anti-inflammatory (IL-4, IL-10) (**J**), and regulatory cytokines (IFN-α, IL-12p70) (**K**) in the collected urine samples from BC patient measured by ELISA among the TIDE subtypes. *p < 0.05, **p < 0.01, ***p < 0.001, ****p < 0.0001; ns, no significance

TME is a highly researched topic, as it plays a crucial role in the initiation, advancement, and management of tumors, specifically in ICB treatment [45]. Therefore, we attempted to characterize the TME patterns for TIDE subtypes. We observed a significant decrease in tumor purity and DNA fraction, and a significant increase in ESTIMATE, Stromal and Immune scores, and the proportion of High-immu subtype from SI to SIII (Fig. 6A, Additional file 1: Figures S8A, B). Hypergeometric test indicated that SI was significantly associated with Low-immu subtype, but the SII and SIII subtypes were significantly associated with the High-immu subtype (Fig. 6B). Additionally, we examined the association of TIDE subtypes with immune phenotypes. It displayed that the desert phenotype was mainly in SI, the inflamed phenotype was primarily in SIII, and the excluded phenotype was mainly in SII (Additional file 1: Figure S8C). These results indicated that the proportion of immune and stromal cells was increased gradually from SI to SIII. Using the SCDC algorithm [46], we calculated the immunocyte abundance in TCGA-BLCA patients. A gradual increase in myeloid cells, CD8Ts and CAFs, and a decrease in B cells, CD4Ts and endothelial cells was found from SI to SIII (Fig. 6C). To further validate our findings, the DECEPTICON and TIDE algorithms [20] were used to evaluate TCGA-BLCA and IMvigor210 [29]. The results were consistent with our findings (Additional file 1: Figure S8D–G). H&E staining of the real-world BC samples showed the gene expression patterns of the TIDE subtypes were histologically related to the abundance of stromal and tumor cells. SI represented abundant tumor cells, and SIII showed highly fibrotic stroma with abundant immunocytes (Fig. 6D). These pathological features were verified using diagnostic slides of TCGA-BLCA (Additional file 1: Figure S8H). We further confirmed the expression levels of fibrosis marker gene FAP and immune marker gene CD45 (PTPRC) across TIDE subtypes. Both RNA-seq analysis and IHC staining, conducted on TCGA-BLCA and real-world samples, consistently revealed increase in FAP and CD45 (PTPRC) expression levels from SI to SIII (Figs. 6E–H). To assess the extent of inflammation in the bladder mucosa, we also analyzed inflammatory factors and cytokines in urine samples from BC patients by enzyme-linked immunosorbent assay (ELISA). The results demonstrated a rise in the concentrations of pro-inflammatory cytokines IL-6 and IL-8 from SI to SIII (Fig. 6I). Conversely, the concentrations of anti-inflammatory cytokines IL-4 and IL-10 decreased from SI to SIII (Fig. 6J). Additionally, the concentrations of regulatory cytokines IFN-γ, IL-12p70 decreased across all three subtypes (Fig. 6K), indicating a suppression of immune activation.

**Fig. 7:**
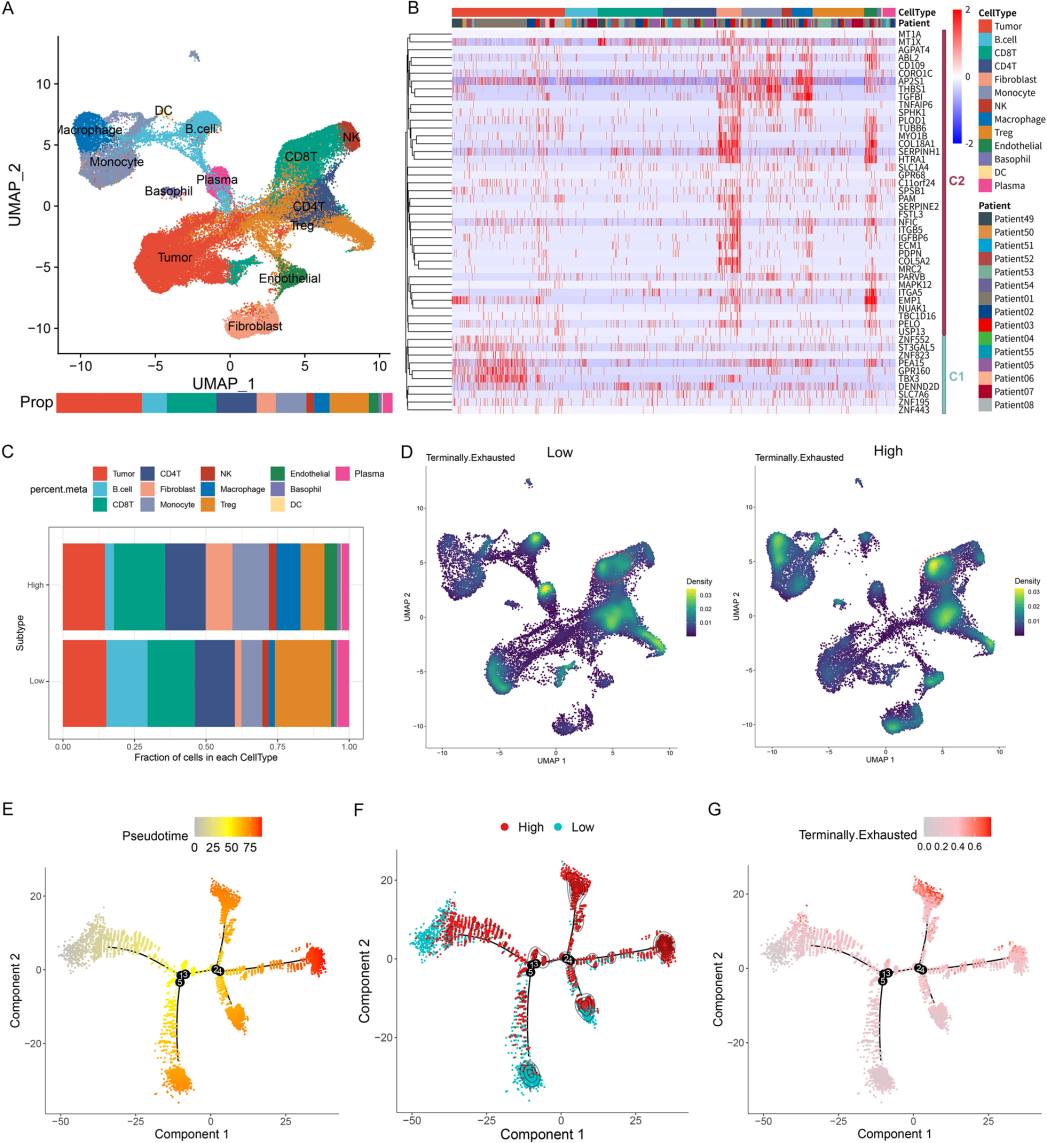
Characterization of TME patterns in the TIDE subtypes based on single-cell RNA-seq dataset. **A** Uniform Manifold Approximation and Projection (UMAP) plot was used to analyze the single-cell RNA-seq dataset. Each color represents one of the 13 cell types in Salomé’s dataset. **B** Heatmap showing the expression levels of TIDE marker genes in 13 cell types. **C** Proportions of 13 cell types in TIDE subtypes. **D** Terminally exhausted signals of two subtype cells assessed by weighted kernel density estimation. Red circle marks CD8Ts. **E**, **F** Differentiation trajectory of CD8^+^ T cells (CD8Ts) in BC, with a color code for pseudotime (**E**) and TIDE subtypes (**F**). (**G**) Expression levels of terminally exhausted signature along the CD8Ts differentiation trajectory in BC

Subsequently, a single-cell RNA-seq (scRNA-seq) dataset published by Salomé et al. [47] was further analyzed to describe the immune landscape of TIDE subtypes. Single-cells were visualized via Uniform Manifold Approximation and Projection (UMAP), and were divided into 31 clusters (Additional file 1: Figure S9A). Each cluster was annotated as a specific cell-type (Fig. 7A, Additional file 1: Figure S9B). The expressions of TIDE marker-genes were examined in each cell-type and found that C1 genes were mainly expressed in tumor cells, and C2 genes were highly expressed in monocytes, macrophages, fibroblasts, and endothelial cells (Fig. 7B). The results confirmed that SI enriched of C1 genes brought higher tumor purity, while SIII enriched of C2 genes carried higher immune and stromal components. To obtain the corresponding TIDE subtypes of these patients, pseudobulk data [48] was obtained from each patient based on scRNA-seq data and consensus clustering [40] was conducted. Patients were divided into two subtypes (Additional file 1: Figure S9D): low expression of C1 and C2 genes (Low), and high expression of C1 and C2 genes (High). By comparing the cell composition, we found that the proportions of monocytes, macrophages, fibroblasts, CD8Ts and endothelial cells were higher in the High subtype, whereas the proportions of Tregs, B cells and plasma cells were at the lower abundance (Fig. 7C). This finding is consistent with our previous deconvolution results. Next, the terminally exhausted signals [49] were evaluated and indicated the High subtype of CD8Ts exhibited a higher level of terminal exhaustion status (Fig. 7D). By trajectory analysis, the High subtype of CD8Ts differentiated into two branches was found as the tumor progressed (Fig. 7E, F). The late differentiating group of CD8Ts did not exhibit significant changes in exhaustion status, whereas the early group displayed severe exhaustion **(**Fig. 7E, G). This specific branch of CD8Ts may represent a critical population for poor prognosis and ICB resistance in High subtype patients. In contrast, the exhaustion status decreased as the tumor progressed in the Low subtype (Fig. 7E, G). Overall, the trend of C2 expression on the trajectory was consistent with the terminal exhaustion signature and further affirmed the reliable subtyping by C2 genes (Additional file 1: Figure S9E).

### Precision treatment of BC following the TIDE subtypes

#### Differential expression of immune checkpoint molecules among the TIDE subtypes

**Fig. 8:**
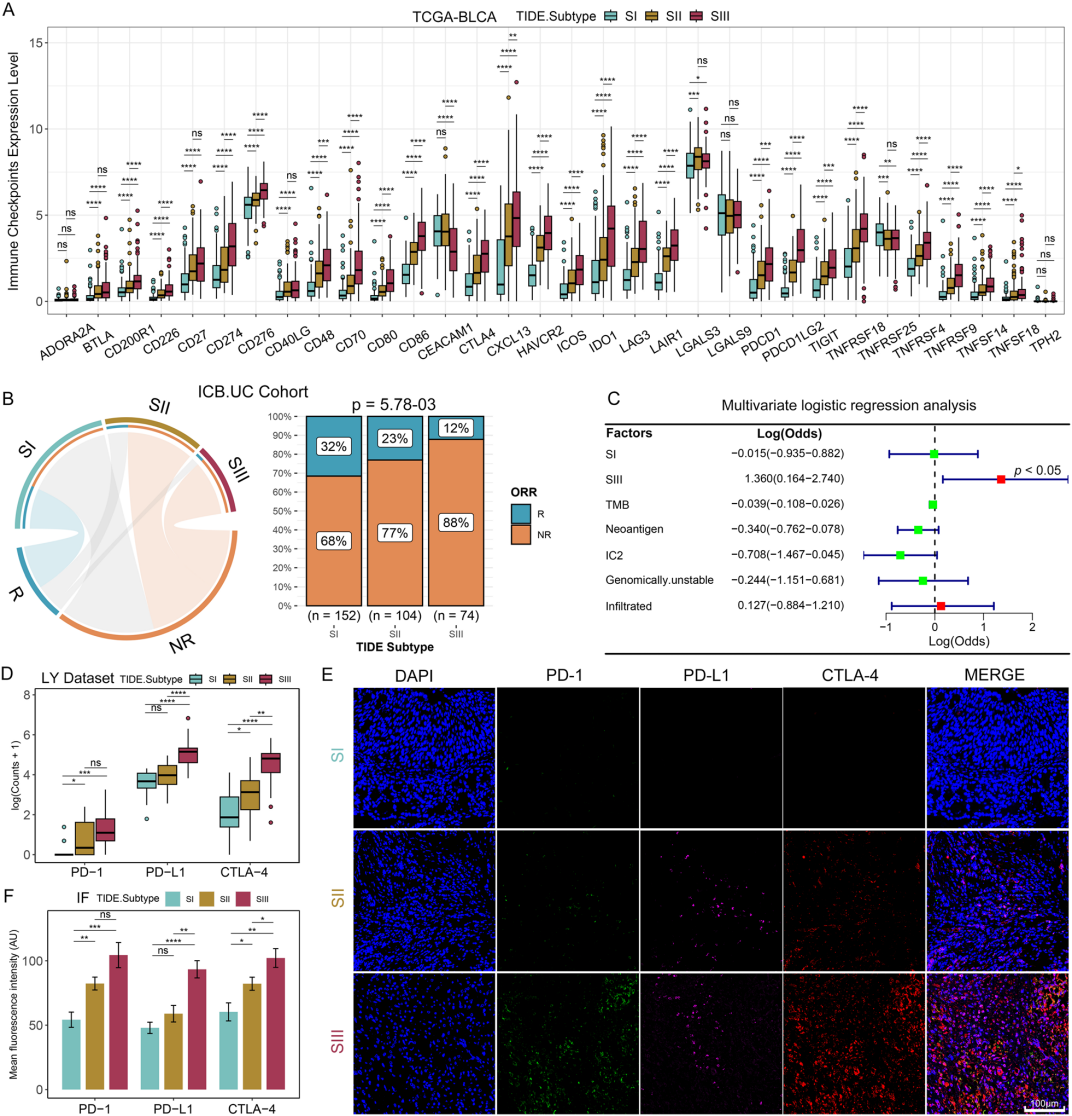
TIDE subtypes were closely related to ICB responses. **A** Comparisons of expression levels of immune checkpoint molecules among the TIDE subtypes of TCGA-BLCA. **B** Hypergeometric test revealed an association between TIDE subtypes of ICB.UC cohort and ICB responses (left), gray lines represent no significance; Stacked histogram showing the differences of ICB responses among the TIDE subtypes of ICB.UC cohort (right). R, response; NR, non-response. **C** Impacts of the TIDE subtypes and other predictive biomarkers on ICB efficacy, which were achieved by multivariate logistic regression analysis. **D** Transcription levels of the representative immune checkpoints including PD-1 (PDCD1), PD-L1 (CD274) and CTLA-4 from LY Dataset among the TIDE subtypes. **E** IF images of PD-1 (PDCD1), PD-L1 (CD274) and CTLA-4 using the collected real-world BC samples among the TIDE subtypes. Scale bar, 100 μm. **F** Analysis of the mean fluorescence intensity (AU) of PD-1 (PDCD1), PD-L1 (CD274) and CTLA-4 among the TIDE subtypes. *p < 0.05, **p < 0.01, ***p < 0.001, ****p < 0.0001; ns, no significance

Since tumor molecular subtyping is pivotal to provide a basis for precise diagnosis and personalized treatment, we further evaluated the potential of TIDE subtypes in guiding precision treatment and drug selection. We found that most immune checkpoint molecules and ligands (such as PD-1, PD-L1, CTLA-4, etc.) were increased from SI to SIII (Fig. 8A, Additional file 1: Figure S10A). In the TIME, CD8Ts are often suppressed and exhausted by immune checkpoint molecules. Meanwhile, exhausted CD8Ts typically express high levels of immune checkpoint molecules [50]. In our study, SIII patients were possibly under this exhausted status, and the observed TIME patterns along with the differential expressions of immune checkpoint molecules could be critical for therapeutic efficacy.

#### Distinct sensitivity to ICB among the TIDE subtypes

We initially examined the profile of the predictive ICB biomarkers in TIDE subtypes. The results showed that the proportions of IC1 and IC2+, as well as TC1 and TC2+, were significantly higher in SIII (Additional file 1: Figure S10B). IC and TC can indicate the PD-L1 levels of immunocytes and tumor cells in the TME patterns. The interferon gamma (IFNG) score was gradually increased from SI to SIII (Additional file 1: Figure S10C, D). Conversely, the proportion of high MSI patients, neoantigen load and immune phenotype score (IPS) were significantly lower in SIII (Additional file 1: Figure S10C, D). Notably, ICB response tends to increase along with the higher level of the abovementioned biomarkers [19, 21, 32, 51]. Therefore, we can find that the prediction of the known ICB biomarkers contains conflicts in evaluating the ICB efficacy of TIDE subtypes.

We further analyzed the ICB response rates of TIDE subtypes in the ICB.UC cohort. The results indicated that SII and SIII were significantly associated with non-responders (NR), SI was closely associated with responders (R), and the proportion of responders were gradually decreased from SI to SIII (Fig. 8B). The analysis of the IMvigor210 dataset produced the consistent results (Additional file 1: Figure S10E). We also analyzed known biomarkers in predicting ICB responses using the IMvigor210 dataset (Additional file 1: Figure S10F). There were no significant differences in ICB response rates between subgroups of TIDE score, MSI, immune phenotype, IPS, and TCGA subtype except for the TMB subgroups. Nanogram showed the SIII contributed the most to ICB non-response (Additional file 1: Figure S10G). Univariate logistic regression analysis indicated that SI was a protective factor, but SIII was a risk factor for ICB response (Additional file 1: Figure S10H). The multivariate logistic regression analysis showed that SIII was the most significant independent predictor of non-response (Fig. 8C, log odds = 1.36, p = 0.036). We also analyzed the predictive value of TIDE subtypes in combination with other biomarkers for ICB outcomes. Receiver operating characteristic (ROC) curves showed that the combined approach of TIDE subtypes and other biomarkers had significantly improved predictive values compared to a single biomarker (Additional file 1: Figure S10I). These findings suggest that TIDE subtype can compensate, to some extent, for the limitations of using a single biomarker in predicting ICB response.

Finally, to validate the above findings, we performed RNA-seq analysis and multiplex immunofluorescence of representative immune checkpoints using the real-world BC samples. The results consistently showed that the expression levels of PD-1 (PDCD1), PD-L1 (CD274) and CTLA-4 were lowest in SI, highest in SIII, and intermediate in SII (Figs. 8D–F), the trend is consistent with public databases (Fig. 8A, Additional file 1: Figure S10A).

#### TIDE subtypes retain sensitivity to specific drugs

**Fig. 9:**
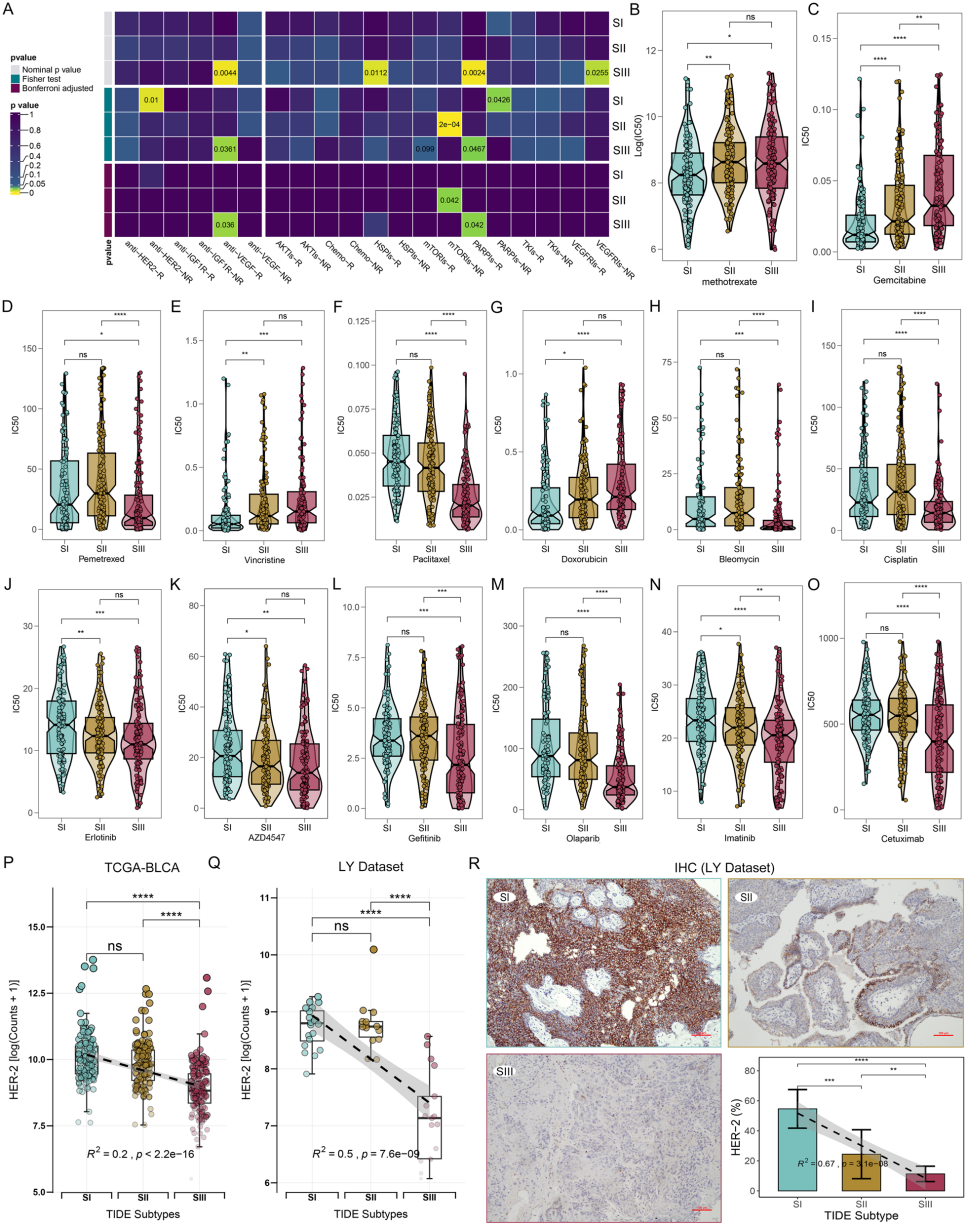
Comparisons of drug sensitivities among the TIDE subtypes of BC. **A** Submap analysis reflects the sensitivity of the TIDE subtypes to the targeted treatments in the BC patients. **B**–**O** Comparisons of the clinically preferred and recommended drugs sensitivity among BC TIDE subtypes: methotrexate (**B**), gemcitabine (**C**), pemetrexed (**D**), vincristine (**E**), paclitaxel (**F**), doxorubicin (**G**), bleomycin (**H**), cisplatin (**I**), erlotinib (**J**), AZD4547 (**K**), gefitinib (**L**), olaparib (**M**), imatinib (**N**), and cetuximab (**O**). **P**, **Q** Transcription levels of HER-2 (ERBB2) among the TIDE subtypes from TCGA-BLCA (**P**) and LY Dataset (**Q**). **R** IHC images of HER-2 from the real-world BC samples and analysis of HER-2 positive cells among the TIDE subtypes. Scale bar, 100 μm. *p < 0.05, **p < 0.01, ***p < 0.001, ****p < 0.0001; ns, no significance

Submap analysis [52] was used to assess the sensitivity of TIDE subtypes to targeted drugs. As shown in Fig. 9A, the SIII subtype tended to be benefited from anti-vascular endothelial growth factor antibodies (anti-VEGF), heat shock protein inhibitors (HPSIs), and poly ADP ribose polymerase inhibitors (PARPIs). This is consistent with the results of a large-scale phase II randomized controlled study, which showed that anti-VEGF combined with ICB treatment significantly improved the OS of patients with ICB-resistant advanced NSCLC [53]. Subsequently, we evaluated the sensitivity of TIDE subtypes to the first-line treatments and recommended drugs. Our findings indicated that certain subtypes showed differential sensitivities to particular drugs. For example, among antimetabolites, SI showed higher sensitivity to methotrexate and gemcitabine (Fig. 9B, C), while SIII was more sensitive to pemetrexed (Fig. 9D). Among plant alkaloids, SI was more responsive to vincristine (Fig. 9E), and SIII showed higher sensitivity to paclitaxel (Fig. 9F). Additionally, SI demonstrated higher sensitivity to anti-tumor antibiotics, such as doxorubicin (Fig. 9G) and epirubicin (Additional file 1: Figure S11A), while SIII was more sensitive to bleomycin (Fig. 9H). Furthermore, our analysis revealed that SIII exhibited higher sensitivity to platinum drugs, including cisplatin (Fig. 9I) and oxaliplatin (Additional file 1: Figure S11B), alkylating agents such as ifosfamide, mitoxantrone and fludarabine (Additional file 1: Figures S11C–E). Consistent with our results, the pemetrexed plus cisplatin can promote immunogenic cell death in models resistant to anti-PD-L1 immunotherapy [54]. In terms of the second-line or recommended drugs, our analysis showed that SI was less responsive to fibroblast growth factor receptor (FGFR) inhibitors, such as erlotinib and AZD4547 (Fig. 9J, K). On the other hand, SIII exhibited higher sensitivity to epidermal growth factor receptor inhibitors (EGFRIs), like gefitinib (Fig. 9L), PARPIs (e.g., olaparib, rucaparib, talazoparib and niraparib) (Fig. 9M, Figure S11F–H), as well as to imatinib (Fig. 9N), and the anti-EGFR antibody cetuximab (Fig. 9O). Similarly, studies have demonstrated that PARPIs [55, 56] and cetuximab [57] can further improve the prognosis of immunotherapy refractory or ineligible patients. Finally, we assessed the expression levels of HER-2 in TIDE subtypes to offer insights into HER-2-targeted therapy. The results of RNA-seq analysis and IHC consistently demonstrated that HER-2 expression was the highest in the SI subtype and the lowest in SIII (Fig. 9P–R). These findings suggest that patients with the SI subtype may have greater potential to benefit from HER-2-targeted therapy.

### Validation of the TIDE-based subtyping strategy in pan-tumors

**Fig. 10:**
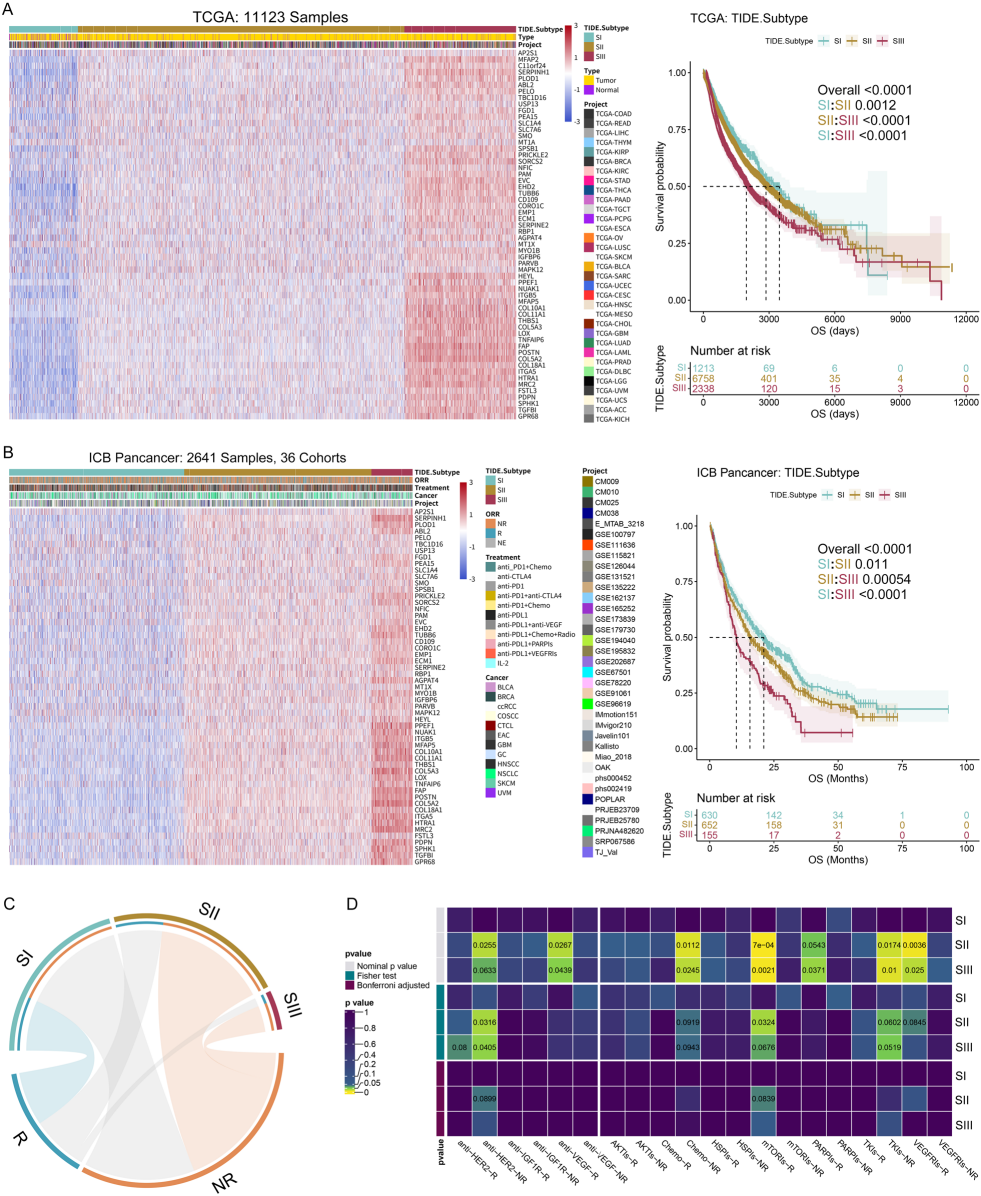
Conservations of the three TIDE subtypes in pan-tumors. **A** Unsupervised hierarchical clustering using the 58 C2 genes to classify pan-tumor samples into three subtypes from TCGA patients (left). K–M analysis shows distinct OS of the TIDE subtypes (right). **B** The same analysis as A. Unsupervised hierarchical clustering of Pan-cancer samples treated with ICB is shown on the left, and K-M analysis showing distinct OS of the TIDE subtypes is on the right. **C** Hypergeometric test collaborates an association of pan-tumor TIDE subtypes with ICB therapy responses. Gray lines represent no significance. **D** Submap analysis reflects the sensitivity of the pan-tumor TIDE subtypes to the targeted treatments

Pan-cancer research enables the application of diagnosis and treatment to a broader range of tumor types by identifying commonalities among them [58, 59]. To determine whether our TIDE subtypes were conserved in pan-cancer, we analyzed five pan-cancer datasets. We initially classified the TCGA pan-cancer samples into three subtypes using 69 TIDE marker genes (Additional file 1: Figure S12A-left). We observed that 11 C1 genes were similarly expressed in pan-cancer samples, and the pan-cancer samples cannot be clustered using these genes (Additional file 1: Figure S12A-right). Therefore, we excluded these 11 genes and used the remaining 58 C2 genes to classify the pan-cancer samples. The results showed that the five pan-cancer datasets were consistently classified into three subtypes: SI (low expression), SII (medium expression), and SIII (high expression) (Fig. 10A, Additional file 1: Figure S12C–F). Moreover, there were significant differences in prognosis among the subtypes (Fig. 10A, Additional file 1: Figure S12B–D), indicating that TIDE subtypes are ubiquitous in pan-tumors.

ICB exerts a remarkable therapeutic effect for many tumors. So, we explored the sensitivity of TIDE subtypes to ICB treatment in pan-tumors. We obtained RNA-seq data from 2641 pan-cancer patients who had received ICB treatment across 12 tumor types, derived from 36 independent datasets [29, 42, 51, 60–89]. Based on the expression levels of C2 genes, we assigned these samples into three subtypes with significantly different prognoses (Fig. 10B, Additional file 1: Figure S12G). In terms of publication bias, we conducted the Peter’s test for the Pan-cancer datasets [43]. The result indicated a certain extent of publication bias probably existed (p = 0.0011), and the funnel plot exhibited asymmetry (Additional file 1: Figure S13C). According to the trim-and-fill analysis, seven putative missing studies were identified on the left side of the distribution (Additional file 1: Figure S13D). The adjusted result of the Peters test revealed no publication bias (p = 0.6037). This demonstrated that publication bias did not substantially affect the overall estimate, and the TIDE-based subtyping strategy applied in the Pan-cancer dataset was relatively robust. Furthermore, hypergeometric test showed that ICB responders were closely related to SI and non-responders were associated with SII and SIII (Fig. 10C). Stacked bar displayed a significant decrease in the response rates from SI to SIII (Additional file 1: Figure S12H). Submap analysis indicated that SIII was mainly correlated with anti-PD-L1 resistance, while SII was associated with IL-2 treatment response (Additional file 1: Figure S12I). Regarding other targeted drugs, SII and SIII were responsive to anti-VEGF, mTORIs, PARPIs, VEGFRIs treatments, but were resistant to anti-HER-2 and TKIs (Fig. 10D).

## Discussion

Tumor immune dysfunction and exclusion are regarded as two main mechanisms of tumor immune escape. The former refers to T cell dysfunction in tumors with high cytotoxic T lymphocyte (CTL) abundance [20, 90]. The persistent antigen stimulation or immunosuppressive signals can trigger functional decline or exhaustion in CTLs, with lower cytokine production and co-stimulatory molecule expression, and higher expression of immune checkpoint receptors and immunosuppressive enzymes [91]. Tumor immune exclusion refers to preventing T cell infiltration in tumors with low CTL abundance, known as “cold tumors” [90, 92]. Tumor cells utilize different mechanisms to obstruct T cell infiltration. The secretion of immunosuppressive factors, like TGF-β, IL-10, and IL-35, will hinder T cell activation and proliferation [93]. The expression of immune checkpoint molecules from cancer cells, that interact with the corresponding receptors and deliver negative signals, impede T cell activity or induce tolerance [50]. The alteration of the physical and metabolic TME characteristics also hamper T cell migration and function, such as interstitial fibrosis, lactate production and hypoxia [29, 94]. The recruitment of other immunosuppressive cells that compete with T cells, such as Tregs, myeloid-derived suppressor cells (MDSCs), and tumor-associated macrophages, will ultimately suppress their anti-tumor response [25, 30, 94].

The development of bioinformatics techniques has propelled unprecedented advances in precision medicine, such as genomic and transcriptomic analyses, and multi-omics integration analysis [95–97]. Elucidation of the specific treatment response changes in the TME patterns and immune cell functional status can provide insights into mechanisms underlying resistance of immune therapy and identify new therapeutic strategies. In our study, transcriptome data was used to evaluate the status of TIDE patterns, and a novel TIDE-based subtyping method was developed for accurately predicting immunotherapy in the BC patients. We found SIII subtype owning the highest immune infiltration. The cytokine, inflammatory response, and immune regulation pathways were highly enriched and activated. The TME analysis indicated that SIII represented the lowest tumor purity, but the highest amount of fibrosis and immunosuppressive cells (macrophages and MDSCs), that would impair ICB efficacy. Moreover, the SIII subtype exhibited elevated CTLs infiltrations, immune checkpoints and ligands, and exhausted CD8Ts, indicating functional defects and exclusion of CTLs in the TME. This immune dysfunction and exclusion would affect immunotherapy and chemotherapy [20, 29]. Additionally, SIII represented reduced B cells and plasma cells, which would also undermine ICB efficacy [27, 77, 98]. Overall, the heterogeneity of TME reflects the resistance of SIII to ICB treatment in the BC patients.

Tumor molecular subtyping is pivotal for cancer diagnosis and treatment, which helps to reflect the TME patterns, select ICB candidates, predict immune-related adverse events, and guide ICB combination strategies [22, 99, 100]. The existing molecular subtyping methods for BC mainly include UNC subtype, MDA subtype, TCGA subtype, Lund subtype, Bold subtype, NAC subtype, NanoString subtype, etc. [22, 100]. However, there is no significant predictive value to identify the response to immune checkpoint inhibitors when using these BC subtypes [22, 23]. Our novel TIDE-based subtyping strategy, in addition to being closely associated with clinicopathological and molecular features in BC patients, has potential application of predicting the response of immunotherapy. Furthermore, we compared the proposed TIDE-based method with previously published predictive immunotherapy biomarkers (including MSI, TMB, neoantigen load, PD-1, PD-L1, CTLA-4, and TIDE score) and existing BC classification methods (including IPS, Immune Phenotype, IC, TC, Lund2, and TCGA Subtype). The result indicates that immunotherapy efficacy was significantly correlated with SI and SIII of TIDE-based subtypes, TMB, neoantigen load, IC2 subtype of IC, Genomically unstable subtype and UroB subtype of Lund2 (Additional file 1: Figure S10H). Nevertheless, only the SIII subtype remains closely associated with immunotherapy efficacy when further controlling for confounding factors and simultaneously considering the impact of the aforementioned factors on immunotherapy (Fig. 8C). It suggests that our TIDE-based subtype can potentially serve as an independent predictive indicator for BC immunotherapy, unlike the existing biomarkers and classification methods. On the other hand, we also found that it possibly exists discrepancies in assessing immunotherapy between the TIDE-based subtyping and the published molecular subtyping methods. For example, SIII represented the highest inflamed phenotype, which normally indicates more responsive to ICB therapy due to high PD-L1 level and abundant tumor-infiltrating lymphocytes [101]. Moreover, it is believed that increased IC1 and IC2+ or TC1 and TC2+ indicate higher objective immunological remission rates [21]. SIII exhibited the highest proportion of IC and TC levels among the subtypes, but in fact, SIII failed to respond to ICB. Therefore, these results indicate the accurate prediction performance of TIDE-based subtyping method in identifying non-responders compared with certain immunotherapy biomarkers. It might suggest an option to combine TIDE subtypes with biomarkers for guiding ICB treatment. We can select patients with inflamed phenotype tumors and further exclude SIII subtype patients for ICB therapy candidates. Based on the IMvigor210 cohort, ROC showed that the immune phenotype alone had lower predictive performance (AUC = 0.55), but it was notably improved with the combination of TIDE subtyping (AUC = 0.65). However, large-scale clinical trials are still needed for future validation.

The TIDE algorithm developed by Liu et al. integrates signatures of T cell dysfunction and T cell exclusion. It exhibited excellent predictive performance for ICB efficacy in melanoma and NSCLC with an AUC up to 80% [20]. However, there is still a lack of evidence to support this method to predict ICB response in BC. Our study showed that the original TIDE algorithm had limited predictive performance for ICB response, and no differences of the ICB response could be detected by the TIDE algorithm (Additional file 1: Figure S10F). Logistic regression analysis showed that TIDE score was not a significant risk factor for immunotherapy (Additional file 1: Figure S10H). ROC curve also indicated that the TIDE scores had no predictive power for ICB efficacy (AUC = 0.57, p > 0.05, Additional file 1: Figure S10I). In addition, the specific calculation method and the genes used by the TIDE algorithm are unknown for users, which further limits the application of the TIDE algorithm in bladder cancer. For example, the “blind box” nature of the TIDE algorithm makes it impossible for users to understand the intrinsic mechanism and biological significance of the TIDE score, and prevents them from verifying and improving the effectiveness and accuracy of the TIDE algorithm. In our study, we identified three TIDE-based subtypes closely associated with ICB efficacy (Fig. 8B, Additional file 1: Figure S10E), which optimized the applicability of the TIDE algorithm in BC. TIDE-based subtype can be used as an independent predictor of BC immunotherapy efficacy (Fig. 8C). Although the TIDE-based subtypes were initially devised for immunotherapy, our comprehensive analysis has unveiled that this molecular classification can also inform the selection of first-line drugs and recommended chemotherapy for BC. The variances in drug sensitivity among the three TIDE subtypes were meticulously assessed utilizing hypergeometric and chi-square tests on immunotherapy RNA-seq data, ridge regression analysis derived from GDSC and CTRP databases, and Submap analysis. These authoritative algorithms serve as pivotal tools for drug evaluation and selection. Nevertheless, further investigation is warranted to elucidate the concordance between drug sensitivity predictions and real-world clinical responses.

In this study, we characterized the TIDE status of BC and developed a TIDE-based subtyping method. Briefly, SI represents the lowest TIDE status, the best prognosis, and it is sensitive to ICB treatment. SIII shows the highest TIDE level, the poorest prognosis, accompanied by suppressive TIME and terminally exhausted T cells, which is sensitive to EGFRIs and PARPIs but resistant to ICB treatment. SII falls into a transitional status with intermediate TIDE level and prognosis. We also validated the conserved characteristics of TIDE subtypes in pan-cancers using five pan-tumor cohorts, one ICB pre-treatment cohort and their responses to immunotherapy. These results suggest that our novel TIDE-based subtyping strategy can be also used for pan-cancers, and potentially bring more benefits for a wider range of cancer patients. In addition, clinicians or biologists can further improve our subtyping method to better suit their own clinical needs. However, this study still has limitations. Firstly, we only provided descriptive conclusions regarding the molecular characteristics of TIDE subtypes, and further research is needed to investigate the underlying mechanisms and effects of these characteristics. Secondly, drug sensitivity assessment lacks in vivo and in vitro validation. Future research needs to explore the biological mechanisms underlying the coherence between TIDE-based drug sensitivity predictions and patient responses. Lastly, prospective clinical trials will further validate our approach by correlating predicted responses to immunotherapy and chemotherapy with real-world observed clinical outcomes.

In conclusion, tumor molecular subtyping represents one future research direction for the personalized cancer therapy, with important implications in guiding clinical treatment and developing new anti-cancer drugs. Our analysis of whole transcriptome data in the BC patients identified three TIDE-based subtypes showing significant differences in clinicopathological and molecular features, as well as functional pathways and treatment responses. This subtyping method can also be applied to pan-cancer. We believe that our novel TIDE-based subtyping strategy has enormous potential for clinical application, as it can assist in making personalized treatment decisions for BC and pan-cancer patients, selecting potential beneficiaries, and excluding resistant patients of ICB therapy.

## Methods

Five bulk RNA-seq datasets and one scRNA-seq dataset of BC, five bulk-RNA-seq cohorts of pan-tumors, one bulk RNA-seq cohort of pan-tumors treated with ICB, and somatic mutation and CNA data from TCGA-BLCA were collected in this study. Details and sources for all datasets are listed in Additional file 1: Table S1. 51 real-world BC samples and urine samples were collected from the Department of Urology, Shanghai Sixth People's Hospital. Detailed clinical information of these patients is available from Additional file 1: Table S2. The overall design of this study is as follows: evaluation of TIDE status and its relationship with BC clinicopathological and molecular features; TIDE subtyping; characterization of clinicopathological and molecular features of TIDE subtypes; and analysis of the pan-tumor landscape of the TIDE subtypes. The analysis methods used include RNA sequencing, differential expression (DE) analysis, clustering analysis, TIDE analysis, Protein–protein interaction network analysis (PPI), pathway analysis, somatic mutation and CNV analysis, survival analysis, IHC, immunofluorescence staining and ELISA, etc. Detailed descriptions of the methods and computational analyses are provided in Additional files 1, 2.

### Supplementary Information

**Additional file 1: Table S1.** Characteristics of bulk RNA-seq datasets and single-cell RNA-seq dataset enrolled in this study. **Table S2.** TIDE subtype and clinical information of LY dataset. **Figure S1.** The overall design of the current study. **Figure S2.** Correlations of TIDE status with clinicopathological and molecular features in the BC patients. **Figure S3.** Correlations of TIDE status with TIME in the BC patients. **Figure S4.** Identification of TIDE marker genes for molecular subtyping. **Figure S5.** Identifications of three BC TIDE subtypes based on TIDE marker genes. **Figure S6.** Comparisons of clinicopathological and molecular features among the TIDE subtypes of BC. **Figure S7.** Signaling pathways and functional annotations of three TIDE subtypes of BC. **Figure S8.** Characterizations of TME patterns among the TIDE subtypes based on bulk RNA-seq datasets. **Figure S9.** Characterizations of TME patterns among the TIDE subtypes based on single-cell RNA-seq dataset. **Figure S10.** TIDE subtypes were closely related to ICB response. **Figure S11.** Comparisons of drug sensitivities and identification of the potential targeted compounds of BC TIDE subtypes. **Figure S12.** Conservations of the TIDE subtypes in pan-tumors. **Figure S13.** Bias analysis and funnel plots of studies investigating the association of TIDE subtypes with OS. **Methods S1.**

**Additional file 2: Data S1.** Details of sixty-nine TIDE marker genes. **Data S2.** Details of thirty TIDE and TIME overlapping genes.

## Data Availability

The original contributions presented in the study are included in the article and supplementary information. The RNA-seq data of the collected BC in the LY dataset has been uploaded to Gene Expression Omnibus (GEO, accession number: GSE248167). The computer R code for processing and analysis of this study is available upon request.

## Abbreviations

BC: Bladder cancer
CAFs: Cancer-associated fibroblasts
CD4Ts: CD4^+^ T cells
CD8Ts: CD8^+^ T cells
CDF: Cumulative distribution function
CNV: Copy number variation
CTL: Cytotoxic T lymphocyte
CTLs: Cytotoxic T-cells
CTRP: Cancer Therapeutics Response Portal
DSS: Disease-specific survival
GDSC: Genomics of Drug Sensitivity in Cancer
GSEA: Gene Set Enrichment Analysis
GSVA: Gene Set Variation Analysis
ICB: Immune checkpoint blockade
IFNG: Interferon gamma
IPA: Ingenuity Pathway Analysis
IPS: Immune phenotype score
irAEs: Immune-related adverse events
MDSC: Myeloid-derived suppressor cells
MSI: Microsatellite instability
NMF: Non-negative matrix factorization
NSCLC: Non-small cell lung cancer
OS: Overall survival
PAC: Proportion of ambiguous clustering
PFI: Progression-free interval
ROC: Receiver operating characteristic
TAM: Tumor-associated macrophages
TID: Tumor immune dysfunction
TIDE: Tumor immune dysfunction and exclusion
TIE: Tumor immune exclusion
TILs: Tumor-infiltrating lymphocytes
TIME: Tumor immune microenvironment
TMB: Tumor mutational burden
TME: Tumor microenvironment
Treg: Regulatory T cells
UMAP: Uniform Manifold Approximation and Projection

## References

[1] Siegel R, Miller K, Fuchs H, Jemal A (2022). Cancer statistics, 2022. CA: a Cancer J Clin.

[2] Chen W, Zheng R, Baade P, Zhang S, Zeng H, Bray F, Jemal A, Yu X, He J (2016). Cancer statistics in China, 2015. CA a Cancer J Clin.

[3] Babjuk M, Burger M, Capoun O, Cohen D, Compérat EM, Dominguez Escrig JL, Gontero P, Liedberg F, Masson-Lecomte A, Mostafid AH (2022). European Association of urology guidelines on non-muscle-invasive bladder cancer (Ta, T1, and Carcinoma in Situ). Eur Urol.

[4] Witjes JA, Bruins HM, Cathomas R, Compérat EM, Cowan NC, Gakis G, Hernández V, Linares Espinós E, Lorch A, Neuzillet Y (2021). European Association of urology guidelines on muscle-invasive and metastatic bladder cancer: summary of the 2020 guidelines. Eur Urol.

[5] Yang J, Jiang X, Chen Y, Teng L (2023). EIF5A2 promotes doxorubicin resistance in bladder cancer cells through the TGF-β signaling pathway. Discov Med.

[6] Cathomas R, Lorch A, Bruins HM, Compérat EM, Cowan NC, Efstathiou JA, Fietkau R, Gakis G, Hernández V, Espinós EL (2022). The 2021 updated european association of urology guidelines on metastatic urothelial carcinoma. Eur Urol.

[7] Lenis AT, Lec PM, Chamie K, Mshs MD (2020). Bladder cancer: a review. JAMA.

[8] Flaig TW, Spiess PE, Abern M, Agarwal N, Bangs R, Boorjian SA, Buyyounouski MK, Chan K, Chang S, Friedlander T (2022). NCCN guidelines® insights: bladder cancer, version 2.2022. J Natl Compr Canc Netw.

[9] Kubli SP, Berger T, Araujo DV, Siu LL, Mak TW (2021). Beyond immune checkpoint blockade: emerging immunological strategies. Nat Rev Drug Discov.

[10] Powles T, Durán I, van der Heijden MS, Loriot Y, Vogelzang NJ, De Giorgi U, Oudard S, Retz MM, Castellano D, Bamias A (2018). Atezolizumab versus chemotherapy in patients with platinum-treated locally advanced or metastatic urothelial carcinoma (IMvigor211): a multicentre, open-label, phase 3 randomised controlled trial. Lancet.

[11] Roviello G, Catalano M, Santi R, Palmieri VE, Vannini G, Galli IC, Buttitta E, Villari D, Rossi V, Nesi G (2021). Immune checkpoint inhibitors in urothelial bladder cancer: state of the art and future perspectives. Cancers (Basel).

[12] Nair SS, Weil R, Dovey Z, Davis A, Tewari AK (2020). The tumor microenvironment and immunotherapy in prostate and bladder cancer. Urol Clin North Am.

[13] Zheng K, Gao L, Hao J, Zou X, Hu X (2022). An immunotherapy response prediction model derived from proliferative CD4(+) T cells and antigen-presenting monocytes in ccRCC. Front Immunol.

[14] Petitprez F, Meylan M, de Reyniès A, Sautès-Fridman C, Fridman WH (2020). The tumor microenvironment in the response to immune checkpoint blockade therapies. Front Immunol.

[15] Ouyang Y, Zhong W, Xu P, Wang B, Zhang L, Yang M, Chen J, Li H, Li S, Chen X (2024). Tumor-associated neutrophils suppress CD8(+) T cell immunity in urothelial bladder carcinoma through the COX-2/PGE2/IDO1 Axis. Br J Cancer.

[16] Chen S, Zeng J, Huang L, Peng Y, Yan Z, Zhang A, Zhao X, Li J, Zhou Z, Wang S (2022). RNA adenosine modifications related to prognosis and immune infiltration in osteosarcoma. J Transl Med.

[17] McGrail DJ, Pilié PG, Rashid NU, Voorwerk L, Slagter M, Kok M, Jonasch E, Khasraw M, Heimberger AB, Lim B (2021). High tumor mutation burden fails to predict immune checkpoint blockade response across all cancer types. Ann Oncol.

[18] Jiang ZR, Yang LH, Jin LZ, Yi LM, Bing PP, Zhou J, Yang JS (2022). Identification of novel cuproptosis-related lncRNA signatures to predict the prognosis and immune microenvironment of breast cancer patients. Front Oncol.

[19] André T, Shiu KK, Kim TW, Jensen BV, Jensen LH, Punt C, Smith D, Garcia-Carbonero R, Benavides M, Gibbs P (2020). Pembrolizumab in microsatellite-instability-high advanced colorectal cancer. N Engl J Med.

[20] Jiang P, Gu S, Pan D, Fu J, Sahu A, Hu X, Li Z, Traugh N, Bu X, Li B (2018). Signatures of T cell dysfunction and exclusion predict cancer immunotherapy response. Nat Med.

[21] Nishino M, Ramaiya NH, Hatabu H, Hodi FS (2017). Monitoring immune-checkpoint blockade: response evaluation and biomarker development. Nat Rev Clin Oncol.

[22] Warrick JI, Alahmadie H, Berman DM, Black PC, Flaig TW, Höglund M, Bubendorf L, van Kwast TH, Cheng L (2024). International society of urological pathology consensus conference on current issues in bladder cancer. Working Group 4: molecular subtypes of bladder cancer-principles of classification and emerging clinical utility. Am J Surg Pathol.

[23] Necchi A, Raggi D, Gallina A, Ross JS, Farè E, Giannatempo P, Marandino L, Colecchia M, Lucianò R, Bianchi M (2020). Impact of molecular subtyping and immune infiltration on pathological response and outcome following neoadjuvant pembrolizumab in muscle-invasive bladder cancer. Eur Urol.

[24] Binnewies M, Roberts EW, Kersten K, Chan V, Fearon DF, Merad M, Coussens LM, Gabrilovich DI, Ostrand-Rosenberg S, Hedrick CC (2018). Understanding the tumor immune microenvironment (TIME) for effective therapy. Nat Med.

[25] Xu L, Zou C, Zhang S, Chu TSM, Zhang Y, Chen W, Zhao C, Yang L, Xu Z, Dong S (2022). Reshaping the systemic tumor immune environment (STIE) and tumor immune microenvironment (TIME) to enhance immunotherapy efficacy in solid tumors. J Hematol Oncol.

[26] Yan J, Liu D, Wang J, You W, Yang W, Yan S, He W (2024). Rewiring chaperone-mediated autophagy in cancer by a prion-like chemical inducer of proximity to counteract adaptive immune resistance. Drug Resist Updat.

[27] Long F, Wang W, Li S, Wang B, Hu X, Wang J, Xu Y, Liu M, Zhou J, Si H (2023). The potential crosstalk between tumor and plasma cells and its association with clinical outcome and immunotherapy response in bladder cancer. J Transl Med.

[28] Gouin KH, Ing N, Plummer JT, Rosser CJ, Ben Cheikh B, Oh C, Chen SS, Chan KS, Furuya H, Tourtellotte WG (2021). An N-Cadherin 2 expressing epithelial cell subpopulation predicts response to surgery, chemotherapy and immunotherapy in bladder cancer. Nat Commun.

[29] Mariathasan S, Turley SJ, Nickles D, Castiglioni A, Yuen K, Wang Y, Kadel EE, Koeppen H, Astarita JL, Cubas R (2018). TGFβ attenuates tumour response to PD-L1 blockade by contributing to exclusion of T cells. Nature.

[30] Loeuillard E, Yang J, Buckarma E, Wang J, Liu Y, Conboy C, Pavelko KD, Li Y, O'Brien D, Wang C (2020). Targeting tumor-associated macrophages and granulocytic myeloid-derived suppressor cells augments PD-1 blockade in cholangiocarcinoma. J Clin Invest.

[31] Hänzelmann S, Castelo R, Guinney J (2013). GSVA: gene set variation analysis for microarray and RNA-seq data. BMC Bioinformatics.

[32] Charoentong P, Finotello F, Angelova M, Mayer C, Efremova M, Rieder D, Hackl H, Trajanoski Z (2017). Pan-cancer immunogenomic analyses reveal genotype-immunophenotype relationships and predictors of response to checkpoint blockade. Cell Rep.

[33] Şenbabaoğlu Y, Gejman RS, Winer AG, Liu M, Van Allen EM, de Velasco G, Miao D, Ostrovnaya I, Drill E, Luna A (2016). Tumor immune microenvironment characterization in clear cell renal cell carcinoma identifies prognostic and immunotherapeutically relevant messenger RNA signatures. Genome Biol.

[34] Yoshihara K, Shahmoradgoli M, Martínez E, Vegesna R, Kim H, Torres-Garcia W, Treviño V, Shen H, Laird PW, Levine DA (2013). Inferring tumour purity and stromal and immune cell admixture from expression data. Nat Commun.

[35] Riester M, Werner L, Bellmunt J, Selvarajah S, Guancial EA, Weir BA, Stack EC, Park RS, O'Brien R, Schutz FA (2014). Integrative analysis of 1q233 copy-number gain in metastatic urothelial carcinoma. Clin Cancer Res.

[36] Robertson AG, Groeneveld CS, Jordan B, Lin X, McLaughlin KA, Das A, Fall LA, Fantini D, Taxter TJ, Mogil LS (2020). Identification of differential tumor subtypes of T1 bladder cancer. Eur Urol.

[37] Choi W, Porten S, Kim S, Willis D, Plimack ER, Hoffman-Censits J, Roth B, Cheng T, Tran M, Lee IL (2014). Identification of distinct basal and luminal subtypes of muscle-invasive bladder cancer with different sensitivities to frontline chemotherapy. Cancer Cell.

[38] Sjödahl G, Lauss M, Lövgren K, Chebil G, Gudjonsson S, Veerla S, Patschan O, Aine M, Fernö M, Ringnér M (2012). A molecular taxonomy for urothelial carcinoma. Clin Cancer Res.

[39] Kim WJ, Kim EJ, Kim SK, Kim YJ, Ha YS, Jeong P, Kim MJ, Yun SJ, Lee KM, Moon SK (2010). Predictive value of progression-related gene classifier in primary non-muscle invasive bladder cancer. Mol Cancer.

[40] Wilkerson MD, Hayes DN (2010). ConsensusClusterPlus: a class discovery tool with confidence assessments and item tracking. Bioinformatics.

[41] Brunet JP, Tamayo P, Golub TR, Mesirov JP (2004). Metagenes and molecular pattern discovery using matrix factorization. Proc Natl Acad Sci U S A.

[42] Snyder A, Nathanson T, Funt SA, Ahuja A, Buros Novik J, Hellmann MD, Chang E, Aksoy BA, Al-Ahmadie H, Yusko E (2017). Contribution of systemic and somatic factors to clinical response and resistance to PD-L1 blockade in urothelial cancer: An exploratory multi-omic analysis. PLoS Med.

[43] Huang R, Li Y, Wu H, Liu B, Zhang X, Zhang Z (2023). (68)Ga-PSMA-11 PET/CT versus (68)Ga-PSMA-11 PET/MRI for the detection of biochemically recurrent prostate cancer: a systematic review and meta-analysis. Front Oncol.

[44] Cancer Genome Atlas Research Network (2014). Comprehensive molecular characterization of urothelial bladder carcinoma. Nature.

[45] Huang AC, Zappasodi R (2022). A decade of checkpoint blockade immunotherapy in melanoma: understanding the molecular basis for immune sensitivity and resistance. Nat Immunol.

[46] Dong M, Thennavan A, Urrutia E, Li Y, Perou CM, Zou F, Jiang Y (2021). SCDC: bulk gene expression deconvolution by multiple single-cell RNA sequencing references. Brief Bioinform.

[47] Salomé B, Sfakianos JP, Ranti D, Daza J, Bieber C, Charap A, Hammer C, Banchereau R, Farkas AM, Ruan DF (2022). NKG2A and HLA-E define an alternative immune checkpoint axis in bladder cancer. Cancer Cell.

[48] Crowell HL, Soneson C, Germain PL, Calini D, Collin L, Raposo C, Malhotra D, Robinson MD (2020). muscat detects subpopulation-specific state transitions from multi-sample multi-condition single-cell transcriptomics data. Nat Commun.

[49] Sade-Feldman M, Yizhak K, Bjorgaard SL, Ray JP, de Boer CG, Jenkins RW, Lieb DJ, Chen JH, Frederick DT, Barzily-Rokni M (2018). Defining T cell states associated with response to checkpoint immunotherapy in melanoma. Cell.

[50] Blank CU, Haining WN, Held W, Hogan PG, Kallies A, Lugli E, Lynn RC, Philip M, Rao A, Restifo NP (2019). Defining 'T cell exhaustion'. Nat Rev Immunol.

[51] Garcia-Diaz A, Shin DS, Moreno BH, Saco J, Escuin-Ordinas H, Rodriguez GA, Zaretsky JM, Sun L, Hugo W, Wang X (2017). Interferon receptor signaling pathways regulating PD-L1 and PD-L2 expression. Cell Rep.

[52] Hoshida Y, Brunet JP, Tamayo P, Golub TR, Mesirov JP (2007). Subclass mapping: identifying common subtypes in independent disease data sets. PLoS ONE.

[53] Reckamp KL, Redman MW, Dragnev KH, Minichiello K, Villaruz LC, Faller B, Al Baghdadi T, Hines S, Everhart L, Highleyman L (2022). Phase II randomized study of ramucirumab and pembrolizumab versus standard of care in advanced non-small-cell lung cancer previously treated with immunotherapy-lung-MAP S1800A. J Clin Oncol.

[54] Limagne E, Nuttin L, Thibaudin M, Jacquin E, Aucagne R, Bon M, Revy S, Barnestein R, Ballot E, Truntzer C (2022). MEK inhibition overcomes chemoimmunotherapy resistance by inducing CXCL10 in cancer cells. Cancer Cell.

[55] Gay CM, Stewart CA, Park EM, Diao L, Groves SM, Heeke S, Nabet BY, Fujimoto J, Solis LM, Lu W (2021). Patterns of transcription factor programs and immune pathway activation define four major subtypes of SCLC with distinct therapeutic vulnerabilities. Cancer Cell.

[56] Franzese O, Graziani G (2022). Role of PARP inhibitors in cancer immunotherapy: potential friends to immune activating molecules and foes to immune checkpoints. Cancers (Basel).

[57] Marin-Acevedo JA, Withycombe BM, Kim Y, Brohl AS, Eroglu Z, Markowitz J, Tarhini AA, Tsai KY, Khushalani NI (2023). Cetuximab for immunotherapy-refractory/ineligible cutaneous squamous cell carcinoma. Cancers (Basel).

[58] He B, Zhang Y, Zhou Z, Wang B, Liang Y, Lang J, Lin H, Bing P, Yu L, Sun D (2020). A neural network framework for predicting the tissue-of-origin of 15 common cancer types based on RNA-Seq data. Front Bioeng Biotechnol.

[59] He B, Sun H, Bao M, Li H, He J, Tian G, Wang B (2023). A cross-cohort computational framework to trace tumor tissue-of-origin based on RNA sequencing. Sci Rep.

[60] Pusztai L, Yau C, Wolf DM, Han HS, Du L, Wallace AM, String-Reasor E, Boughey JC, Chien AJ, Elias AD (2021). Durvalumab with olaparib and paclitaxel for high-risk HER2-negative stage II/III breast cancer: results from the adaptively randomized I-SPY2 trial. Cancer Cell.

[61] Wolf DM, Yau C, Wulfkuhle J, Brown-Swigart L, Gallagher RI, Lee PRE, Zhu Z, Magbanua MJ, Sayaman R, O'Grady N (2022). Redefining breast cancer subtypes to guide treatment prioritization and maximize response: predictive biomarkers across 10 cancer therapies. Cancer Cell.

[62] Keenan TE, Guerriero JL, Barroso-Sousa R, Li T, O'Meara T, Giobbie-Hurder A, Tayob N, Hu J, Severgnini M, Agudo J (2021). Molecular correlates of response to eribulin and pembrolizumab in hormone receptor-positive metastatic breast cancer. Nat Commun.

[63] Choueiri TK, Fishman MN, Escudier B, McDermott DF, Drake CG, Kluger H, Stadler WM, Perez-Gracia JL, McNeel DG, Curti B (2016). Immunomodulatory activity of nivolumab in metastatic renal cell carcinoma. Clin Cancer Res.

[64] Mahoney KM, Ross-Macdonald P, Yuan L, Song L, Veras E, Wind-Rotolo M, McDermott DF, Stephen Hodi F, Choueiri TK, Freeman GJ (2022). Soluble PD-L1 as an early marker of progressive disease on nivolumab. J Immunother Cancer.

[65] Braun DA, Hou Y, Bakouny Z, Ficial M, Sant' Angelo M, Forman J, Ross-Macdonald P, Berger AC, Jegede OA, Elagina L (2020). Interplay of somatic alterations and immune infiltration modulates response to PD-1 blockade in advanced clear cell renal cell carcinoma. Nat Med.

[66] Motzer RJ, Rini BI, McDermott DF, Redman BG, Kuzel TM, Harrison MR, Vaishampayan UN, Drabkin HA, George S, Logan TF (2015). Nivolumab for metastatic renal cell carcinoma: results of a randomized phase II trial. J Clin Oncol.

[67] Motzer RJ, Tannir NM, McDermott DF, Arén Frontera O, Melichar B, Choueiri TK, Plimack ER, Barthélémy P, Porta C, George S (2018). Nivolumab plus ipilimumab versus sunitinib in advanced renal-cell carcinoma. N Engl J Med.

[68] Ascierto ML, McMiller TL, Berger AE, Danilova L, Anders RA, Netto GJ, Xu H, Pritchard TS, Fan J, Cheadle C (2016). The intratumoral balance between metabolic and immunologic gene expression is associated with anti-PD-1 response in patients with renal cell carcinoma. Cancer Immunol Res.

[69] Motzer RJ, Banchereau R, Hamidi H, Powles T, McDermott D, Atkins MB, Escudier B, Liu LF, Leng N, Abbas AR (2020). Molecular subsets in renal cancer determine outcome to checkpoint and angiogenesis blockade. Cancer Cell.

[70] Motzer RJ, Robbins PB, Powles T, Albiges L, Haanen JB, Larkin J, Mu XJ, Ching KA, Uemura M, Pal SK (2020). Avelumab plus axitinib versus sunitinib in advanced renal cell carcinoma: biomarker analysis of the phase 3 JAVELIN Renal 101 trial. Nat Med.

[71] Phillips D, Matusiak M, Gutierrez BR, Bhate SS, Barlow GL, Jiang S, Demeter J, Smythe KS, Pierce RH, Fling SP (2021). Immune cell topography predicts response to PD-1 blockade in cutaneous T cell lymphoma. Nat Commun.

[72] van den Ende T, de Clercq NC, van Berge Henegouwen MI, Gisbertz SS, Geijsen ED, Verhoeven RHA, Meijer SL, Schokker S, Dings MPG, Bergman J (2021). Neoadjuvant chemoradiotherapy combined with atezolizumab for resectable esophageal adenocarcinoma: a single-arm phase II feasibility trial (PERFECT). Clin Cancer Res.

[73] Zhao J, Chen AX, Gartrell RD, Silverman AM, Aparicio L, Chu T, Bordbar D, Shan D, Samanamud J, Mahajan A (2019). Immune and genomic correlates of response to anti-PD-1 immunotherapy in glioblastoma. Nat Med.

[74] Kim ST, Cristescu R, Bass AJ, Kim KM, Odegaard JI, Kim K, Liu XQ, Sher X, Jung H, Lee M (2018). Comprehensive molecular characterization of clinical responses to PD-1 inhibition in metastatic gastric cancer. Nat Med.

[75] Cho JW, Hong MH, Ha SJ, Kim YJ, Cho BC, Lee I, Kim HR (2020). Genome-wide identification of differentially methylated promoters and enhancers associated with response to anti-PD-1 therapy in non-small cell lung cancer. Exp Mol Med.

[76] Jung H, Kim HS, Kim JY, Sun JM, Ahn JS, Ahn MJ, Park K, Esteller M, Lee SH, Choi JK (2019). DNA methylation loss promotes immune evasion of tumours with high mutation and copy number load. Nat Commun.

[77] Patil NS, Nabet BY, Müller S, Koeppen H, Zou W, Giltnane J, Au-Yeung A, Srivats S, Cheng JH, Takahashi C (2022). Intratumoral plasma cells predict outcomes to PD-L1 blockade in non-small cell lung cancer. Cancer Cell.

[78] Auslander N, Zhang G, Lee JS, Frederick DT, Miao B, Moll T, Tian T, Wei Z, Madan S, Sullivan RJ (2018). Robust prediction of response to immune checkpoint blockade therapy in metastatic melanoma. Nat Med.

[79] Pomeranz Krummel DA, Nasti TH, Izar B, Press RH, Xu M, Lowder L, Kallay L, Rupji M, Rosen H, Su J (2020). Impact of sequencing radiation therapy and immune checkpoint inhibitors in the treatment of melanoma brain metastases. Int J Radiat Oncol Biol Phys.

[80] Hugo W, Zaretsky JM, Sun L, Song C, Moreno BH, Hu-Lieskovan S, Berent-Maoz B, Pang J, Chmielowski B, Cherry G (2016). Genomic and transcriptomic features of response to anti-PD-1 therapy in metastatic melanoma. Cell.

[81] Riaz N, Havel JJ, Makarov V, Desrichard A, Urba WJ, Sims JS, Hodi FS, Martín-Algarra S, Mandal R, Sharfman WH (2017). Tumor and microenvironment evolution during immunotherapy with nivolumab. Cell.

[82] Liu D, Schilling B, Liu D, Sucker A, Livingstone E, Jerby-Arnon L, Zimmer L, Gutzmer R, Satzger I, Loquai C (2019). Integrative molecular and clinical modeling of clinical outcomes to PD1 blockade in patients with metastatic melanoma. Nat Med.

[83] Gide TN, Quek C, Menzies AM, Tasker AT, Shang P, Holst J, Madore J, Lim SY, Velickovic R, Wongchenko M (2019). Distinct immune cell populations define response to anti-pd-1 monotherapy and anti-PD-1/Anti-CTLA-4 combined therapy. Cancer Cell.

[84] Kraehenbuehl L, Holland A, Armstrong E, O'Shea S, Mangarin L, Chekalil S, Johnston A, Bomalaski JS, Erinjeri JP, Barker CA (2022). Pilot trial of arginine deprivation plus nivolumab and ipilimumab in patients with metastatic uveal melanoma. Cancers (Basel).

[85] Miao D, Margolis CA, Gao W, Voss MH, Li W, Martini DJ, Norton C, Bossé D, Wankowicz SM, Cullen D (2018). Genomic correlates of response to immune checkpoint therapies in clear cell renal cell carcinoma. Science.

[86] Liu S, Knochelmann HM, Lomeli SH, Hong A, Richardson M, Yang Z, Lim RJ, Wang Y, Dumitras C, Krysan K (2021). Response and recurrence correlates in individuals treated with neoadjuvant anti-PD-1 therapy for resectable oral cavity squamous cell carcinoma. Cell Rep Med.

[87] Obradovic A, Graves D, Korrer M, Wang Y, Roy S, Naveed A, Xu Y, Luginbuhl A, Curry J, Gibson M (2022). Immunostimulatory cancer-associated fibroblast subpopulations can predict immunotherapy response in head and neck cancer. Clin Cancer Res.

[88] Chiappinelli KB, Strissel PL, Desrichard A, Li H, Henke C, Akman B, Hein A, Rote NS, Cope LM, Snyder A (2017). Inhibiting DNA methylation causes an interferon response in cancer via dsRNA including endogenous retroviruses. Cell.

[89] Lauss M, Donia M, Harbst K, Andersen R, Mitra S, Rosengren F, Salim M, Vallon-Christersson J, Törngren T, Kvist A (2017). Mutational and putative neoantigen load predict clinical benefit of adoptive T cell therapy in melanoma. Nat Commun.

[90] Joyce JA, Fearon DT (2015). T cell exclusion, immune privilege, and the tumor microenvironment. Science.

[91] Gajewski TF, Schreiber H, Fu YX (2013). Innate and adaptive immune cells in the tumor microenvironment. Nat Immunol.

[92] Liang W, Liu H, Zeng Z, Liang Z, Xie H, Li W, Xiong L, Liu Z, Chen M, Jie H (2023). KRT17 promotes T-lymphocyte infiltration through the YTHDF2-CXCL10 axis in colorectal cancer. Cancer Immunol Res.

[93] Kohli K, Pillarisetty VG, Kim TS (2022). Key chemokines direct migration of immune cells in solid tumors. Cancer Gene Ther.

[94] Watson MJ, Vignali PDA, Mullett SJ, Overacre-Delgoffe AE, Peralta RM, Grebinoski S, Menk AV, Rittenhouse NL, DePeaux K, Whetstone RD (2021). Metabolic support of tumour-infiltrating regulatory T cells by lactic acid. Nature.

[95] Bagaev A, Kotlov N, Nomie K, Svekolkin V, Gafurov A, Isaeva O, Osokin N, Kozlov I, Frenkel F, Gancharova O (2021). Conserved pan-cancer microenvironment subtypes predict response to immunotherapy. Cancer Cell.

[96] Zhou L, Zhang Q, Deng H, Ou S, Liang T, Zhou J (2022). The SNHG1-centered ceRNA network regulates cell cycle and is a potential prognostic biomarker for hepatocellular carcinoma. Tohoku J Exp Med.

[97] Zhu Y, Huang R, Wu Z, Song S, Cheng L, Zhu R (2021). Deep learning-based predictive identification of neural stem cell differentiation. Nat Commun.

[98] Helmink BA, Reddy SM, Gao J, Zhang S, Basar R, Thakur R, Yizhak K, Sade-Feldman M, Blando J, Han G (2020). B cells and tertiary lymphoid structures promote immunotherapy response. Nature.

[99] Smelser WW, Woolbright BL, Taylor JA (2019). Molecular subtyping of bladder cancer: current trends and future directions in 2019. Curr Opin Urol.

[100] Wang S, Yuan X, Shen Z, Zhao J, Zheng B, Zhang J, Ge C (2023). Therapeutic responses to chemotherapy or immunotherapy by molecular subtype in bladder cancer patients: a meta-analysis and systematic review. Investig Clin Urol.

[101] Sobottka B, Nowak M, Frei AL, Haberecker M, Merki S, Levesque MP, Dummer R, Moch H, Koelzer VH (2021). Establishing standardized immune phenotyping of metastatic melanoma by digital pathology. Lab Invest.

