# Supplementary material for: Tumor immune dysfunction and exclusion subtypes in bladder cancer and pan-cancer: a novel molecular subtyping strategy and immunotherapeutic prediction model
Source: J Transl Med. 2024 Apr 17;22:365. doi: 10.1186/s12967-024-05186-8 (PMC11025237; doi:10.1186/s12967-024-05186-8)
Supplement: Supplementary file 1 — Additional file 1: Table S1. Characteristics of bulk RNA-seq datasets and single-cell RNA-seq dataset enrolled in this study. Table S2. TIDE subtype and clinical information of LY dataset. Figure S1. The overall design of the current study. Figure S2. Correlations of TIDE status with clinicopathological and molecular features in the BC patients. Figure S3. Correlations of TIDE status with TIME in the BC patients. Figure S4. Identification of TIDE marker genes for molecular subtyping. Figure S5. Identifications of three BC TIDE subtypes based on TIDE marker genes. Figure S6. Comparisons of clinicopathological and molecular features among the TIDE subtypes of BC. Figure S7. Signaling pathways and functional annotations of three TIDE subtypes of BC. Figure S8. Characterizations of TME patterns among the TIDE subtypes based on bulk RNA-seq datasets. Figure S9. Characterizations of TME patterns among the TIDE subtypes based on single-cell RNA-seq dataset. Figure S10. TIDE subtypes were closely related to ICB response. Figure S11. Comparisons of drug sensitivities and identification of the potential targeted compounds of BC TIDE subtypes. Figure S12. Conservations of the TIDE subtypes in pan-tumors. Figure S13. Bias analysis and funnel plots of studies investigating the association of TIDE subtypes with OS. Methods S1. [file 12967_2024_5186_MOESM1_ESM.pdf]

Tumor immune dysfunction and exclusion subtypes in bladder cancer and pan-cancer: a novel molecular subtyping strategy and immunotherapeutic prediction model

Kun Zheng[1†], Youlong Hai[1†], Hongqi Chen[2†], Yukun Zhang[3], Xiaoyong Hu[1*], Kai Ni[1*]

[1]Department of Urology, Shanghai Sixth People's Hospital Affiliated to Shanghai Jiao Tong University School of Medicine, 200233, Shanghai, China.
[2]Department of Urology, Jiangsu Shengze Hospital of Nanjing Medical University, 215200, Suzhou, Jiangsu, China.
[3]Beijing University of Chinese Medicine East Hospital, Zaozhuang Hospital, 277000, Zaozhuang, Shandong, China.

*** Correspondence:** Kai Ni and Xiaoyong Hu

† These authors contributed equally to this study.

Additional file 1

Additional Tables

- Table S1
- Table S2

Additional Figures

- Figure S1
- Figure S2
- Figure S3
- Figure S4
- Figure S5
- Figure S6
- Figure S7
- Figure S8
- Figure S9
- Figure S10
- Figure S11
- Figure S12
- Figure S13

Additional Methods

- Study Design
- Datasets collection
- Real-world bladder tumor samples collection
- Bulk RNA sequencing and processing
- Single-cell RNA-seq dataset processing
- Differential expression (DE) analysis
- Tumor immune dysfunction (TID) and tumor immune exclusion (TIE)

analysis

- Protein–protein interaction network analysis (PPI)
- Clustering analysis
- Signaling pathway analysis
- Somatic mutation and CNV analysis
- Survival Analysis
- Bias Analysis
- Evaluation of tumor immune microenvironment (TIME) patterns
- Evaluation of immunotherapy and targeted therapeutic efficacy
- Evaluation of drug sensitivity
- Identification of TIDE marker genes for TIDE subtyping
- Hematoxylin-eosin (H&E) and immunohistochemistry (IHC) staining
- Enzyme-linked immunosorbent assay (ELISA)
- Immunofluorescent (IF) staining
- CT-based calculation of tumor volume
- Statistical analysis

Additional References

Table S1. Characteristics of bulk RNA-seq datasets and single-cell RNA-seq dataset enrolled in this study.

a. Bulk RNA-seq datasets of bladder cancer

Datasets	Samples		Age (yr)	pT/cT					pN/cN		pM/cM		Gender		Survival	TMB	Purity	Ref.	Download
	T	N		T0	T1	T2	T3	T4	N0	N1-N3	M0	M1	Male	Female					
**TCGA-BLCA**	412	19	69 (34-90)	1	5	123	207	62	249	138	211	11	314	117	OS, PFI, DSS, DFI	√	√		TCGA
**GSE31684**	93		69 (42-91)	5	10	17	42	19	49	28	57	36	68	25	OS, DSS, RFS			[1]	GEO
**GSE154261**	99														RFS, PFS			[2]	GEO
**GSE48075**	142		69 (43-89)	38	33	41	22	8	123	12	119	8	52	17	OS, DSS			[3]	GEO
**GSE32894**	345	25	71 (20-96)	116	97	85	7	1	49	22			228	80	OS			[4]	GEO
**GSE13507**	188	67	66 (24-88)	24	80	31	19	11	149	15	158	7	135	30	OS			[5]	GEO

b. Bulk RNA-seq datasets of pan-tumors

Datasets	Samples			Age (yr)	Tissue	Cancer type	Gender		Survival	Download
	Total	T	N				Male	Female		
**TCGA**	11123	10391	732	61 (14-90)	138	33	5344	5739	OS, PFI, DFI, DSS	TCGA
**PCAWG**	1466	1305	161	61 (17-90)	22	29	691	654	OS	UCSC Xena
**ICGC**	8746	8097	649	60 (14-90)	16	21	4030	4716	OS	UCSC Xena
**TARGET**	734	723	11	4 (0-30)		7	321	293		UCSC Xena
**GSE2109**	2158	2158			192		697	1458		GEO

c. Baseline bulk RNA-seq datasets of pan-tumors treated with immune-checkpoint blockade therapy

Datasets	Patients	Cancer type	Treatment	Response			OS	PFS	Gender		Ref.	Download
				R	NR	NE			Male	Female		
**IMvigor210**	348	BLCA	anti-PDL1	68	230	50	√		272	76	[6]	R package
**Kallisto**	25	BLCA	anti-PDL1	7	14	4	√	√	22	3	[7]	zenodo
**GSE111636**	11	BLCA	anti-PD1	6	5							GEO

**GSE173839**	71	BRCA	anti-PDL1+PARPIs	29	42					71	[8]	GEO
**GSE194040**	69	BRCA	anti-PD1+Chemotherapy	31	38					69	[9]	GEO
**phs002419**	14	BRCA	anti-PD1+Chemotherapy	4	9	1	√	√		14	[10]	dbGaP
**Checkmate009**	16	KIRC	anti-PD1	3	13		√	√	13	3	[11, 12]	ArrayExpress
**Checkmate010**	45	KIRC	anti-PD1	11	34		√	√	30	15	[12, 13]	Supplements
**Checkmate025**	120	KIRC	anti-PD1	25	86	9	√	√	94	26	[12, 14]	EGA
**E_MTAB_3218**	59	KIRC	anti-PD1	13	43	3	√	√	39	20	[15]	ArrayExpress
**Miao_2018**	33	KIRC	anti-PD1/anti-PDL1/anti-PD1+anti-CTLA4	8	25		√	√	24	9	[16]	
**GSE67501**	11	KIRC	anti-PD1	4	7				7	4	[17]	GEO
**IMmotion151**	407	KIRC	anti-PDL1+anti-VEGF	150	230	27		√	281	126	[18]	EGA
**Javelin101**	354	KIRC	anti-PDL1+VEGFRIs					√	257	97	[19]	Supplements
**GSE179730**	11	COSCC	anti-PD1	3	8		√	√			[20]	GEO
**GSE162137**	25	CTCL	anti-PD1	11	14						[21]	GEO
**GSE165252**	35	EAC	anti-PD1+Chemoterapy+Radiotherapy	12	20	3					[22]	GEO
**PRJNA482620**	17	GBM	anti-PD1	10	7		√				[23]	NCBI
**PRJEB25780**	45	GC	anti-PD1	12	33						[24]	NCBI
**GSE195832**	28	HNSCC	anti-PD1	9	19				26	2	[25]	Mendeley Data
**TJ_Val**	20	HNSCC	anti-PD1	5	15						[25]	Mendeley Data
**GSE126044**	16	NSCLC	anti-PD1	5	11						[26]	GEO
**GSE135222**	27	NSCLC	anti-PD1					√	22	5	[27]	GEO
**OAK**	344	NSCLC	anti-PDL1	48	270	26	√	√	219	125	[28]	EGA
**POPLAR**	95	NSCLC	anti-PDL1	13	74	8	√	√	68	27	[28]	EGA
**Checkmate038**	49	SKCM	anti-PD1	9	34	6	√	√	24	25	[15]	ArrayExpress
**GSE115821**	14	SKCM	anti-PD1/ anti-PD1+anti-CTLA4/ anti-CTLA4	2	12						[29]	GEO
**GSE131521**	17	SKCM	anti-PD1				√				[30]	GEO
**GSE78220**	27	SKCM	anti-PD1	15	12		√		19	8	[31]	GEO
**GSE91061**	51	SKCM	anti-PD1	10	39	2					[32]	GEO
**phs000452**	116	SKCM	anti-PD1/ anti-CTLA4	48	68		√	√	67	49	[33]	dbGaP
**PRJEB23709**	73	SKCM	anti-PD1/ anti-PD1+anti-CTLA4	40	33		√	√	47	26	[34]	NCBI
**SRP067586**	9	SKCM	anti-CTLA4	4	5		√		5	4	[35]	NCBI

**GSE100797**	25	SKCM	IL-2	10	15	√	√	[36]	GEO
**GSE96619**	5	SKCM	anti-PD1	2	3			[37]	GEO
**GSE202687**	9	UVM	anti-PD1+anti-CTLA4		9			[38]	GEO

d. Single-cell RNA-seq dataset of bladder cancer

Dataset	Patients Number	Samples Number	Total cells	Tumor cells	CD8Ts	CD4Ts	Monocytes	Macrophages	Treg	Plasma	B cells	Fibroblasts	Endothelial	Ref.	Download
**Salomé's dataset**	15	17	70244	17884	10303	8456	6383	3311	8153	2037	5202	3988	2034	[39]	Mendeley Data

TMB = tumor mutation load

pT/cT = pathological/clinical tumor stage

pN/cN = pathological/clinical lymph node status (0 = no lymph node metastases, 1~3 = lymph node metastases)

pM/cM = pathological/clinical metastasis status (0 = no distant metastases, 1 = distant metastases)

Samples: T = tumor samples, N = benign samples

Survival: OS = overall survival, PFI = progression-free interval, DFI = disease-free interval, DSS = disease-specific survival, RFS = recurrence-free survival, PFS = progression-free interval

Cancer type: BLCA = bladder cancer, BRCA = breast cancer, KIRC = kidney clear cell carcinoma, COSCC = oral-cavity squamous cell carcinoma, EAC = esophageal adenocarcinoma, GBM = Glioblastoma, GC = gastric cancer, HNSCC = head and neck squamous cell carcinoma, NSCLC = non-small cell lung cancer, SKCM = melanoma, UVM = uveal melanoma, CTCL = cutaneous T cell lymphoma

Response: R = response, NR = non-response, NE = not evaluation

Table S2. TIDE subtype and clinical information of LY dataset.

Patient	TIDE Subtype	pT	Pathological Diagnosis	RNA-seq Samples
**N1**	SI	Ta	High-grade papillary urothelial carcinoma	2
**N2**	SII		Inverted urothelial papilloma	1
**N3**	SI/SII	Ta	High-grade papillary urothelial carcinoma	2
**N4**	SIII	T1	High-grade invasive urothelial carcinoma	2
**N5**	SIII	Tis	Urothelial carcinoma in situ	2
**N6**	SI		Inverted urothelial papilloma	1
**N7**	SI	Ta	High-grade papillary urothelial carcinoma	1
**N8**	SI	Ta	High-grade papillary urothelial carcinoma	1
**N9**	SIII	Ta	High-grade papillary urothelial carcinoma	1
**N10**	SIII	T2	High-grade papillary urothelial carcinoma	1
**N11**	SIII	T4	High-grade invasive urothelial carcinoma	2
**N12**	SII	T1	High-grade papillary urothelial carcinoma	2
**N13**	SIII	T2	High-grade invasive urothelial carcinoma	2
**N14**	SIII	Tis	Urothelial carcinoma in situ	2
**N16**	SI	Ta	Low-grade papillary urothelial carcinoma	1
**N17**	SII/SIII	Ta	Low-grade papillary urothelial carcinoma	2
**N18**	SIII	Ta	Urothelial neoplasms of low malignant potential	1
**N19**	SII/SIII	T1	High-grade invasive urothelial carcinoma	2
**N20**	SII	Ta	High-grade papillary urothelial carcinoma	1
**N21**	SI	Ta	Low-grade papillary urothelial carcinoma	2
**N22**	SI	Ta	Low-grade papillary urothelial carcinoma	2
**N23**	SII	Ta	High-grade papillary urothelial carcinoma	1
**N24**	SI	Ta	Low-grade papillary urothelial carcinoma	1
**N25**	SI/SII	Ta	Low-grade papillary urothelial carcinoma	2
**N26**	SII	Ta	Low-grade papillary urothelial carcinoma	2
**N27**	SIII	T3	High-grade invasive urothelial carcinoma	2
**N28**	SI	Ta	Low-grade papillary urothelial carcinoma	2
**N29**	SI	T1	High-grade papillary urothelial carcinoma	2
**N30**	SII	Ta	Low-grade papillary urothelial carcinoma	2
**N31**	SI/SII	T1	High-grade papillary urothelial carcinoma	2
**N32**	SI	Ta	Low-grade papillary urothelial carcinoma	2

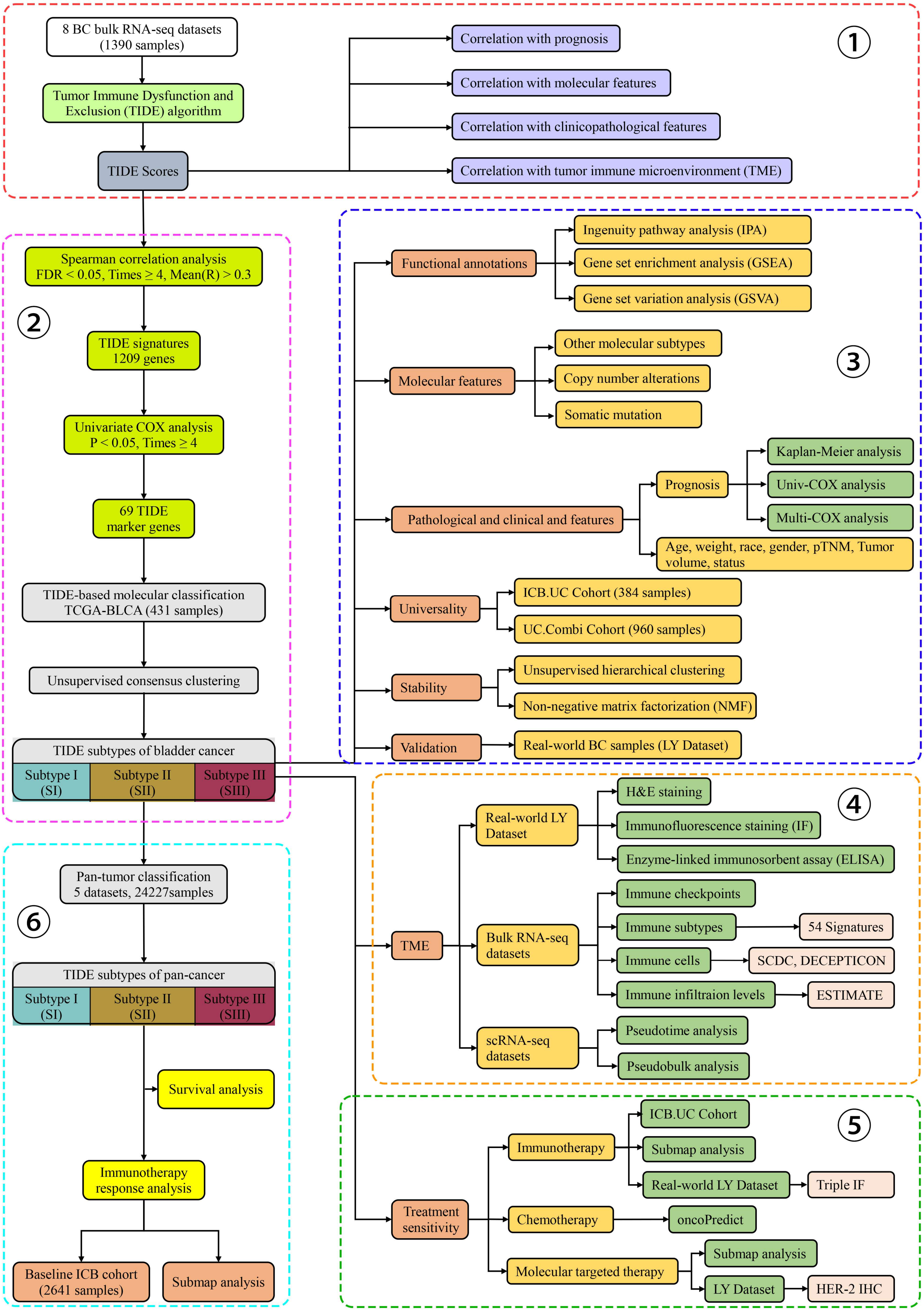

Figure S1. The overall design of the current study (related to Figure 1).

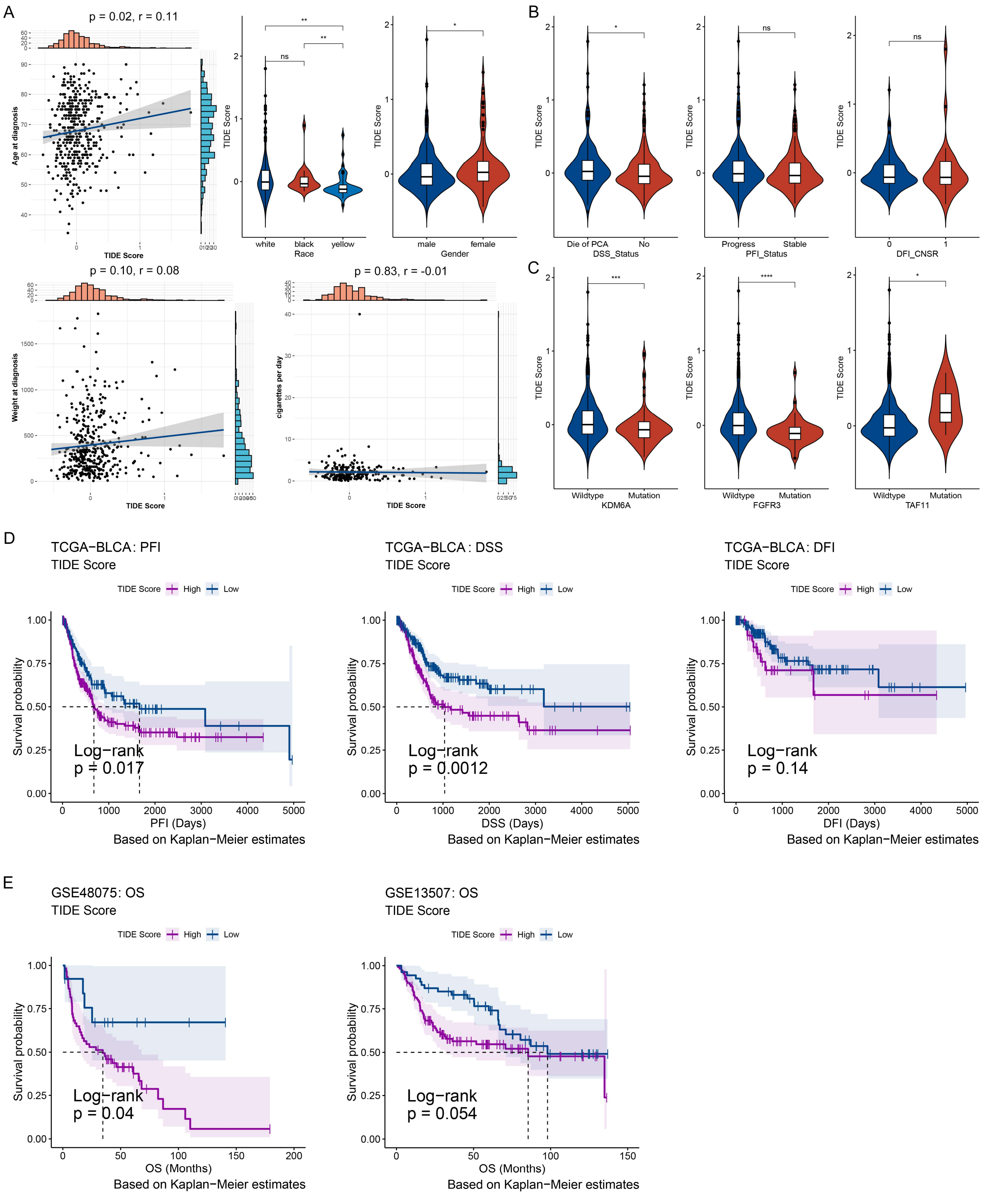

Figure S2. Correlations of TIDE status with clinicopathological and molecular features in the BC patients (related to Figure 2). (**A)** Correlations of TIDE scores with age and weight at diagnosis, race, gender, and daily smoking in BC patients. **(B)** Comparisons of TIDE scores with disease specific death (DSS_Status), progress (PFI_Status) and disease presence (DFI_Status). **(C)** Comparisons of TIDE scores between mutant and wild-type patients at KDM6A, FGFR3 and TAF11 genes. **(D, E)** Kaplan-Meier (K-M) analysis demonstrated a correlation between TIDE scores and the prognosis of BC patients from TCGA-BLCA **(D)**, GSE48075 and GSE13507 **(E)** datasets. OS, overall survival; PFI, progression-free interval; DFI, disease-free interval; DSS, disease-specific survival. Dashed line: median survival time. Color range: 95% confidence interval (CI). *p < 0.05, **p < 0.01, ***p < 0.001, ****p < 0.0001; ns, no significance.

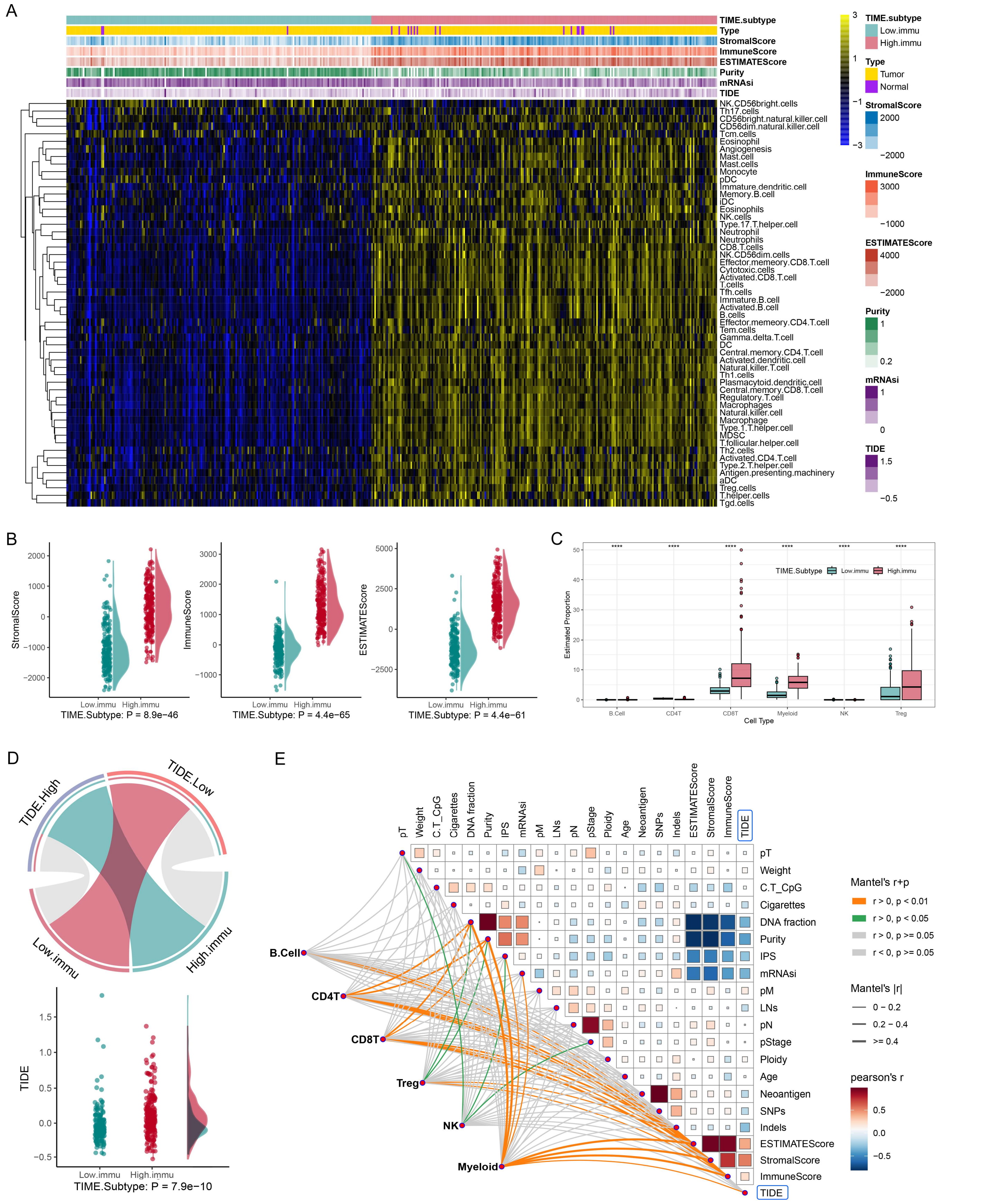

Figure S3. Correlations of TIDE status with TIME in the BC patients. (A) Consensus clustering divided BC patients from TCGA-BLCA into two subtypes according to the activity scores of 54 published immune signatures: low immune infiltration (Low.immu) and high immune infiltration (High.immu) subtypes. **(B)** Raincloudplot shows the TME scores (StromalScore, ImmuneScore, ESTIMATEScore) of BC patients in two TIME subtypes. **(C)** Comparisons of immunocyte abundance among the TIME subtypes of TCGA-BLCA. Cell proportions are assessed by the DECEPTICON algorithm. **(D)** The association between TIME subtypes and TIDE groups, achieved by the hypergeometric test. Gray lines represent no significance. The TIDE groups were dichotomized at the median TIDE scores **(Upper)**. Raincloud plot showing the TIDE levels in BC patients

among the TIME subtypes **(Under)**. **(E)** Heatmaps showing correlations of TIDE scores with clinical and molecular features, and tumor microenvironment (TME) scores (Stromal, Immune, ESTIMATE scores), achieved by Pearson correlation analysis. Links showing the correlations between immunocyte abundance and TIDE scores, TME scores, and clinical and molecular features, implemented by Mantel test. IPS, Immunophenotype score; LNs, Number of lymph nodes examined; SNPs, single nucleotide polymorphisms. The square size represents the absolute value of Pearson's R.

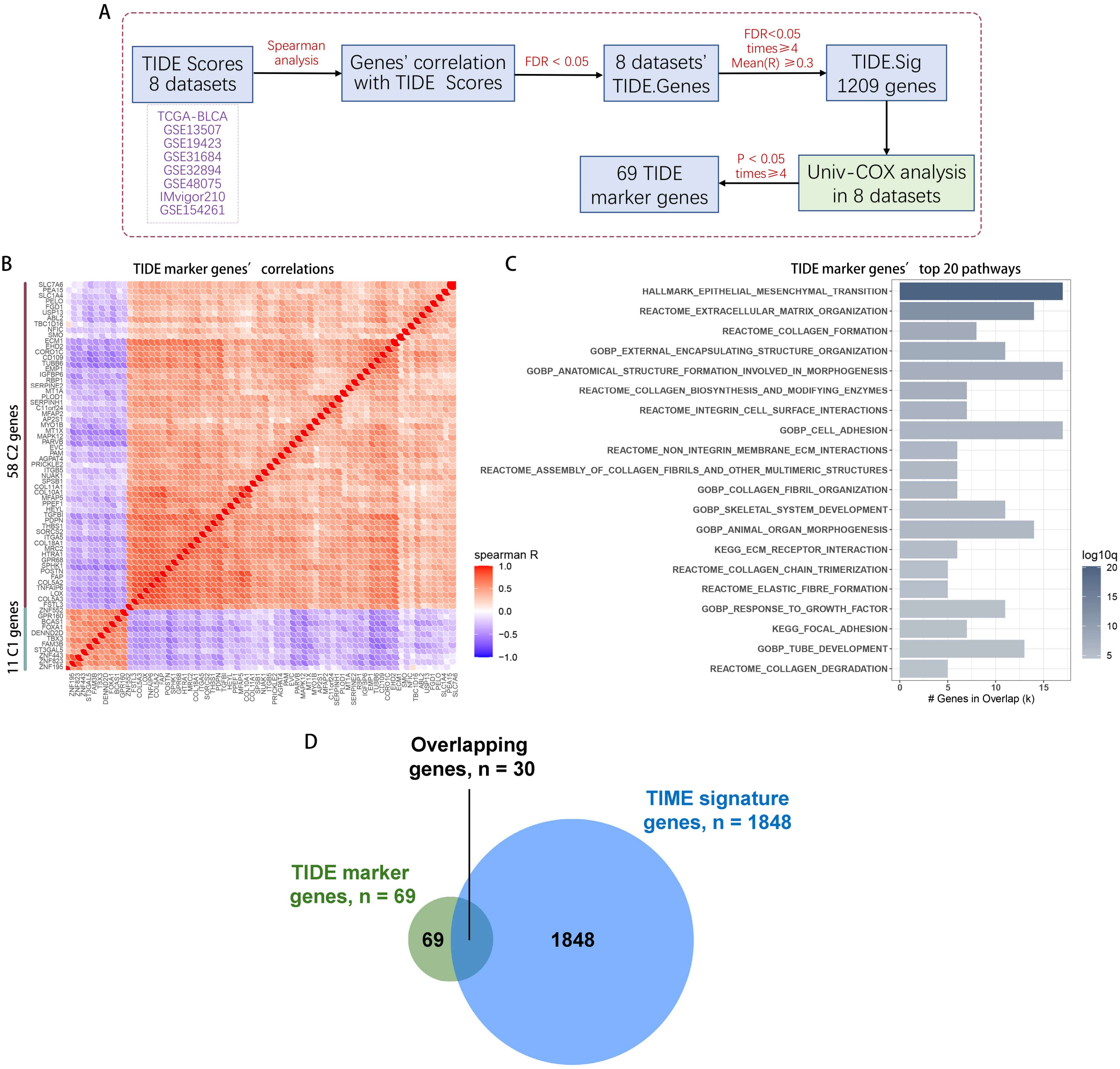

Figure S4. Identification of TIDE marker genes for molecular subtyping. (A) The workflow of TIDE marker genes identification. **(B)** Correlation heatmap of the 69 TIDE marker genes. These genes are mainly divided into two clusters: C1 consists of 11 genes and C2 comprises 58 genes. **(C)** Top 20 signaling pathways enriched based on the 58 C2 genes. **(D)** The Venn diagram reveals an overlap of 30 genes between the TIDE marker genes (n = 69) and the TIME signature genes (n = 1848). The TIME signature genes were identified following the workflow in the Figure S4A.

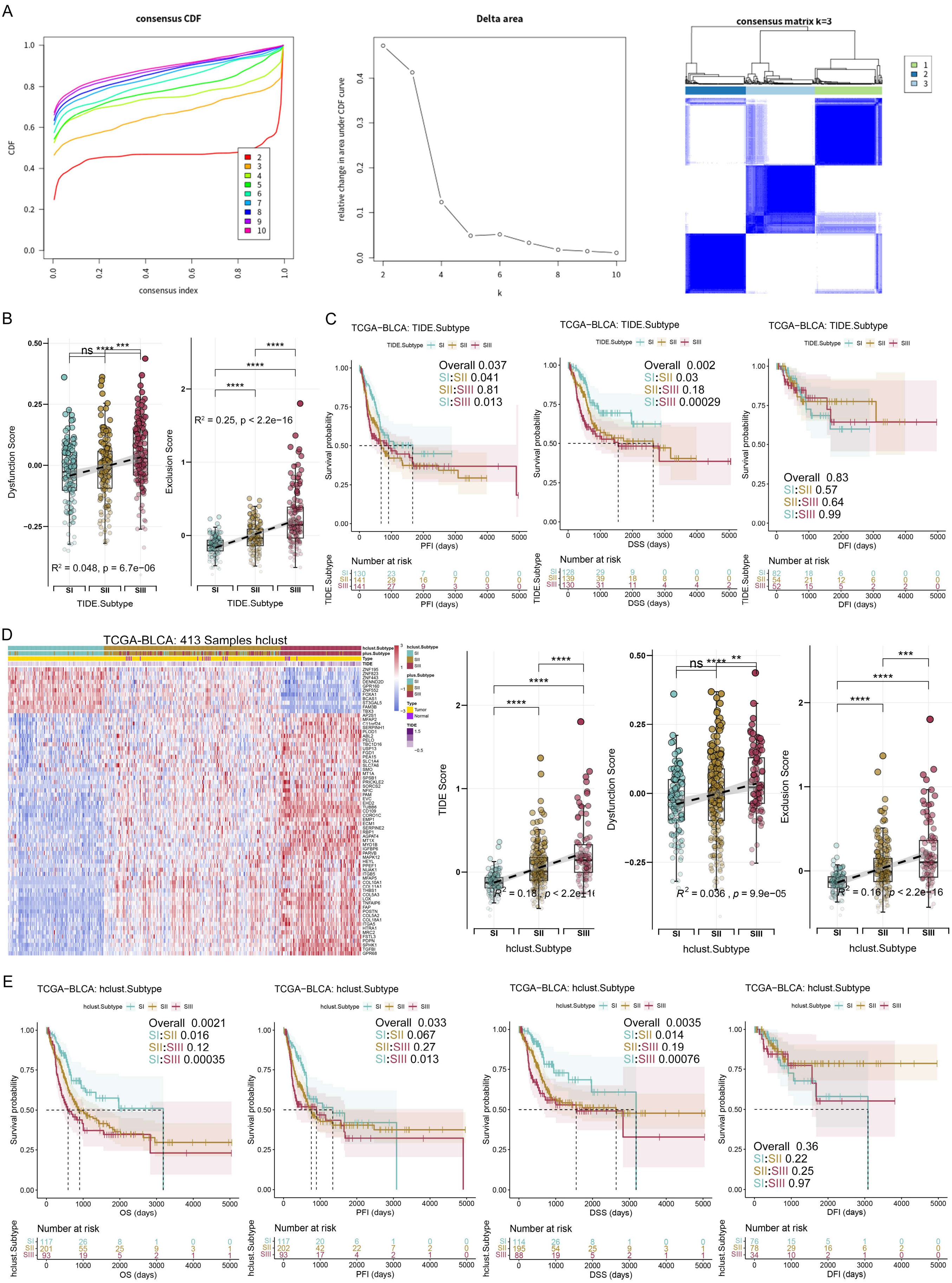
A
consensus CDF
CDF
consensus index
Delta area
relative change in area under CDF curve
k
consensus matrix k=3
B
Dysfunction Score
TIDE.Subtype
R² = 0.048, p = 6.7e−06
Exclusion Score
R² = 0.25, p < 2.2e−16
C
TCGA−BLCA: TIDE.Subtype
Overall 0.037
SI:SII 0.041
SII:SIII 0.81
SI:SIII 0.013
PFI (days)
Overall 0.002
SI:SII 0.03
SII:SIII 0.18
SI:SIII 0.00029
DSS (days)
Overall 0.83
SI:SII 0.57
SII:SIII 0.64
SI:SIII 0.99
DFI (days)
Survival probability
Number at risk
D
TCGA-BLCA: 413 Samples hclust
TIDE Score
R² = 0.18, p < 2.2e−16
Dysfunction Score
R² = 0.036 , p = 9.9e−05
Exclusion Score
R² = 0.16, p < 2.2e−16
hclust.Subtype
E
TCGA−BLCA: hclust.Subtype
Overall 0.0021
SI:SII 0.016
SII:SIII 0.12
SI:SIII 0.00035
OS (days)
Overall 0.033
SI:SII 0.067
SII:SIII 0.27
SI:SIII 0.013
PFI (days)
Overall 0.0035
SI:SII 0.014
SII:SIII 0.19
SI:SIII 0.00076
DSS (days)
Overall 0.36
SI:SII 0.22
SII:SIII 0.25
SI:SIII 0.97
DFI (days)
Number at risk

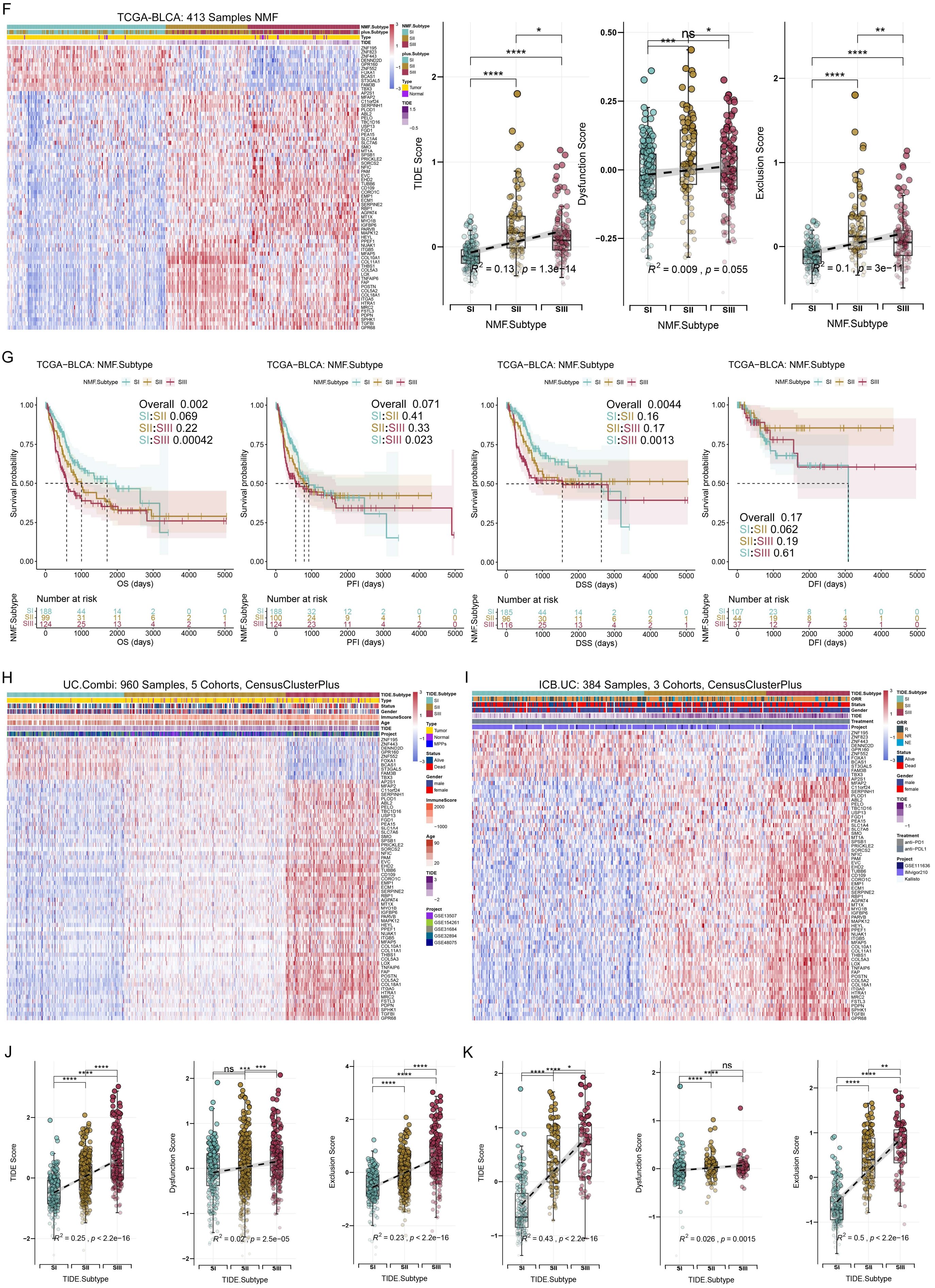

F
TCGA-BLCA: 413 Samples NMF
NMF.Subtype
plus.Subtype
Type
TIDE
TIDE Score
Dysfunction Score
Exclusion Score
NMF.Subtype
R^2 = 0.13 , p = 1.3e−14
R^2 = 0.009 , p = 0.055
R^2 = 0.1 , p = 3e−11
G
TCGA−BLCA: NMF.Subtype
Overall 0.002
SI:SII 0.069
SII:SIII 0.22
SI:SIII 0.00042
Overall 0.071
SI:SII 0.41
SII:SIII 0.33
SI:SIII 0.023
Overall 0.0044
SI:SII 0.16
SII:SIII 0.17
SI:SIII 0.0013
Overall 0.17
SI:SII 0.062
SII:SIII 0.19
SI:SIII 0.61
Survival probability
OS (days)
PFI (days)
DSS (days)
DFI (days)
Number at risk
H
UC.Combi: 960 Samples, 5 Cohorts, CensusClusterPlus
I
ICB.UC: 384 Samples, 3 Cohorts, CensusClusterPlus
J
R^2 = 0.25 , p < 2.2e−16
R^2 = 0.02 , p = 2.5e−05
R^2 = 0.23 , p < 2.2e−16
K
R^2 = 0.43 , p < 2.2e−16
R^2 = 0.026 , p = 0.0015
R^2 = 0.5 , p < 2.2e−16
TIDE.Subtype

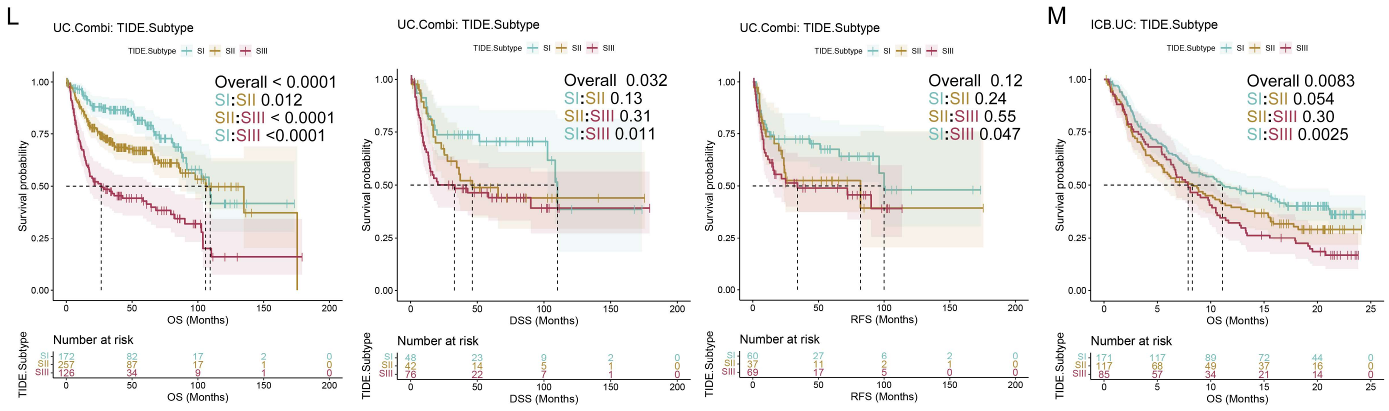

Figure S5. Identifications of three BC TIDE subtypes based on TIDE marker genes (related to Figure 3). **(A)** Cumulative distribution function (CDF) curves of the consensus score from k = 2 to 10 (left). The relative change in the area under the CDF curve from k = 2 to 10 (**medium**). Consensus matrix for k = 3, which was the optimal cluster number (**right**). **(B)** Levels and trends of Dysfunction scores and Exclusion scores among the TIDE subtypes. **(C)** K-M analysis shows significant differences in PFI and DSS among the TIDE subtypes of TCGA-BLCA. **(D)** Unsupervised hierarchical clustering (hclust) based on the expression of the 69 TIDE marker genes classified TCGA-BLCA patients into three subtypes: Subtype I (SI), Subtype II (SII), and Subtype III (SIII) **(left)**. Levels and trends of TIDE, Dysfunction and Exclusion scores among the TIDE subtypes **(right)**. **(E)** K-M analysis of the TIDE subtypes of TCGA-BLCA classified by hclust. **(F)** Unsupervised nonnegative matrix factorization (NMF) based on the expression of the 69 TIDE marker genes classified TCGA-BLCA patients into three subtypes: SI, SII and SIII **(left)**. Levels and trends of TIDE, Dysfunction and Exclusion scores among the TIDE subtypes **(right)**. **(G)** K-M analysis of the TIDE subtypes of TCGA-BLCA classified by NMF. **(H, I)** Consensus clustering based on the expression of 69 TIDE marker genes classified UC.Combi **(H)** and ICB.UC **(I)** cohorts into three subtypes. **(J, K)** Levels and trends of TIDE, Dysfunction and Exclusion scores among the TIDE subtypes of UC.Combi **(J)** and ICB.UC **(K)** cohorts. **(L, M)** K-M analysis of the TIDE subtypes of UC.Combi **(L)** and ICB.UC **(M)** cohorts. *p < 0.05, **p < 0.01, ***p < 0.001, ****p < 0.0001; ns, no significance.

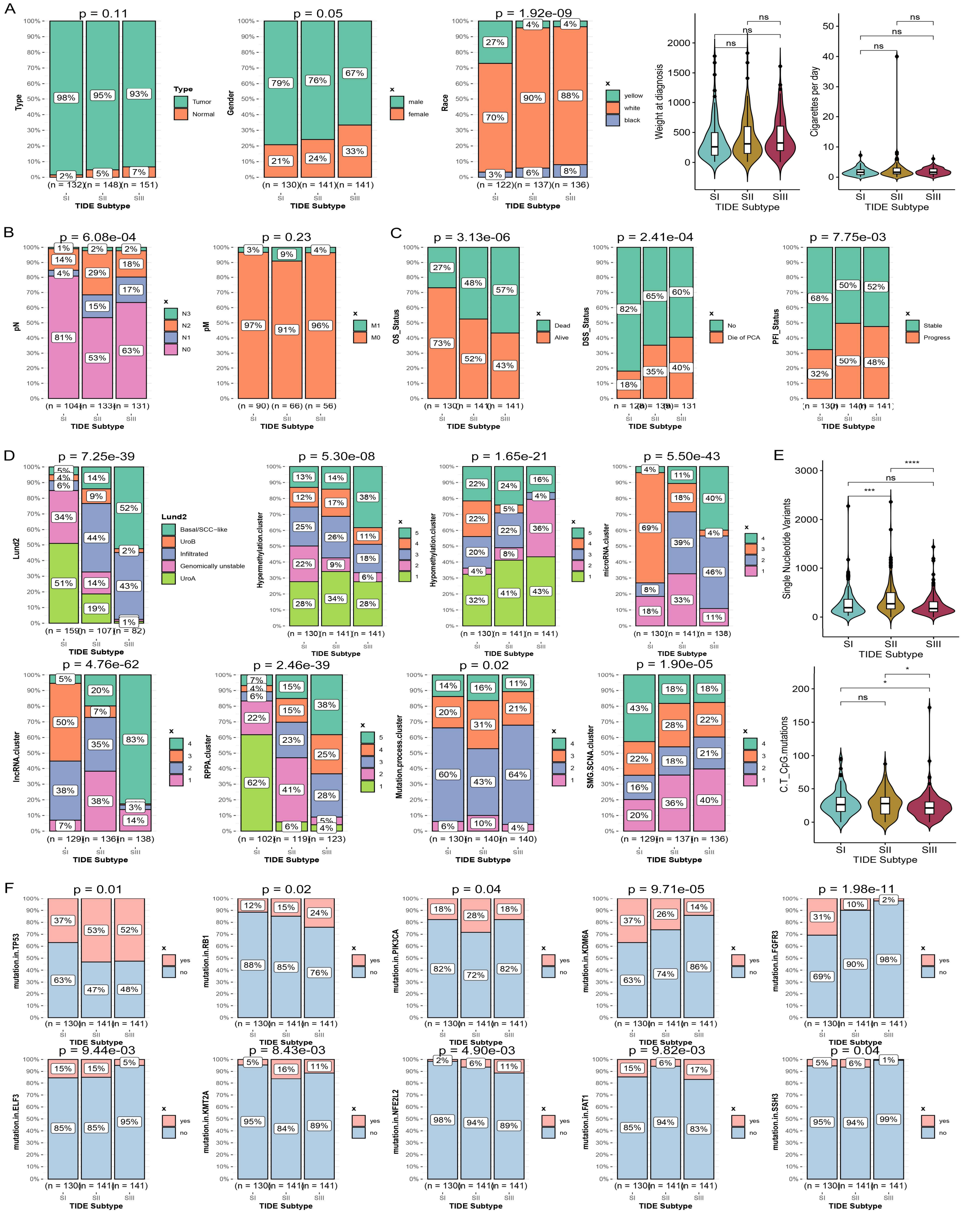

Figure S6. Comparisons of clinicopathological and molecular features among the TIDE subtypes of BC (related to Figure 4). **(A)** Comparisons of sample type, gender, race, weight at diagnosis, and daily smoking among three TIDE subtypes. **(B)** The proportions of pathological N stage (pN) and pathological M stage (pM) among the TIDE subtypes. **(C)** The proportions of OS_Status, DSS_Status and DFI_Status among the TIDE subtypes. **(D)** Associations and similarities of TIDE subtypes with other molecular subtypes. **(E)** Comparisons of single nucleotide variants **(upper)** and C/T CpG mutations **(under)** among the TIDE subtypes. **(F)** Comparisons of mutations in commonly identified biomarkers among the three TIDE subtypes in BC patients. *p < 0.05, ***p < 0.001, ****p < 0.0001; ns, no significance.

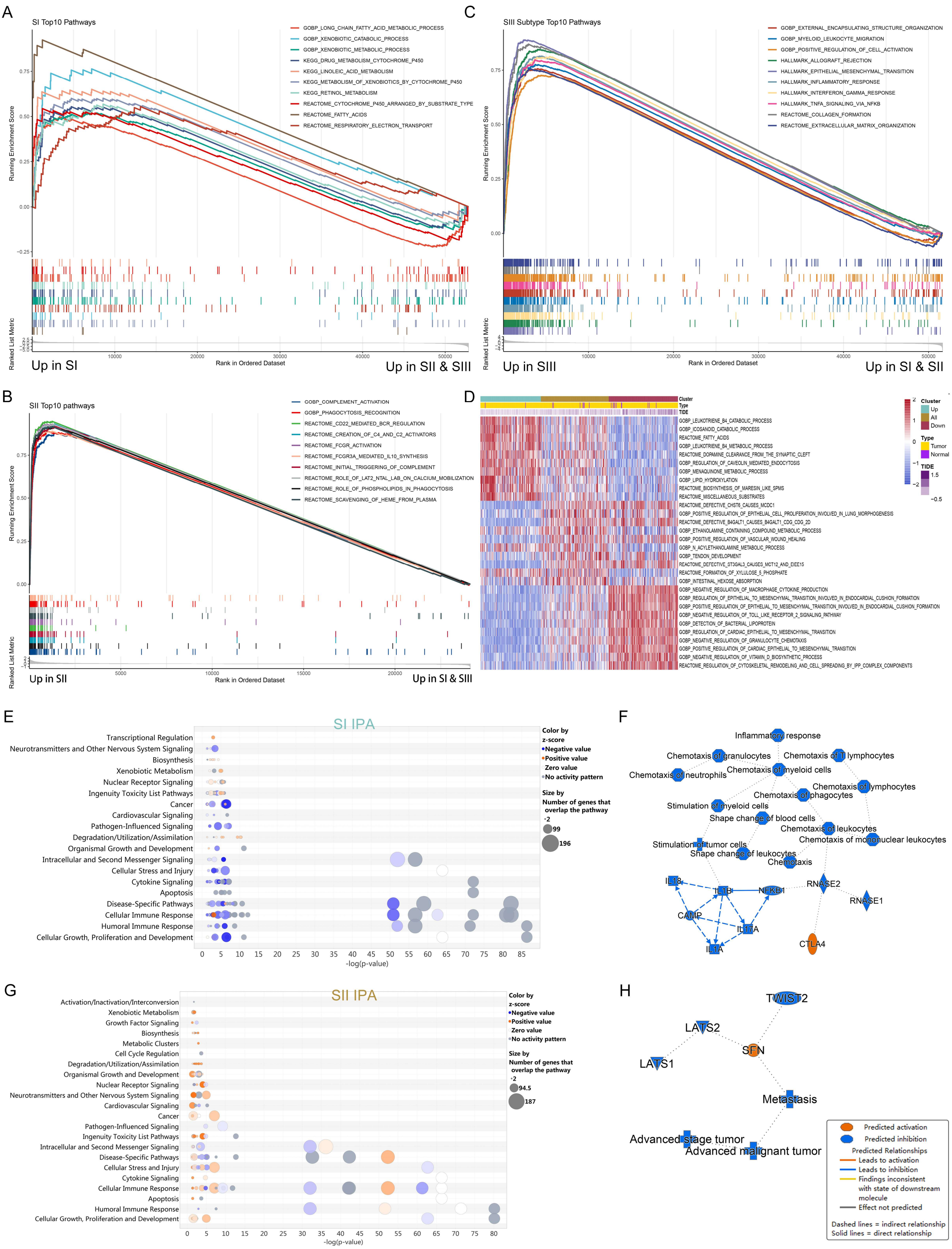

A
SI Top10 Pathways
GOBP_LONG_CHAIN_FATTY_ACID_METABOLIC_PROCESS
GOBP_XENOBIOTIC_CATABOLIC_PROCESS
GOBP_XENOBIOTIC_METABOLIC_PROCESS
KEGG_DRUG_METABOLISM_CYTOCHROME_P450
KEGG_LINOLEIC_ACID_METABOLISM
KEGG_METABOLISM_OF_XENOBIOTICS_BY_CYTOCHROME_P450
KEGG_RETINOL_METABOLISM
REACTOME_CYTOCHROME_P450_ARRANGED_BY_SUBSTRATE_TYPE
REACTOME_FATTY_ACIDS
REACTOME_RESPIRATORY_ELECTRON_TRANSPORT
Running Enrichment Score
Ranked List Metric
Rank in Ordered Dataset
Up in SI
Up in SII & SIII
C
SIII Subtype Top10 Pathways
GOBP_EXTERNAL_ENCAPSULATING_STRUCTURE_ORGANIZATION
GOBP_MYELOID_LEUKOCYTE_MIGRATION
GOBP_POSITIVE_REGULATION_OF_CELL_ACTIVATION
HALLMARK_ALLOGRAFT_REJECTION
HALLMARK_EPITHELIAL_MESENCHYMAL_TRANSITION
HALLMARK_INFLAMMATORY_RESPONSE
HALLMARK_INTERFERON_GAMMA_RESPONSE
HALLMARK_TNFA_SIGNALING_VIA_NFKB
REACTOME_COLLAGEN_FORMATION
REACTOME_EXTRACELLULAR_MATRIX_ORGANIZATION
Up in SIII
Up in SI & SII
B
SII Top10 pathways
GOBP_COMPLEMENT_ACTIVATION
GOBP_PHAGOCYTOSIS_RECOGNITION
REACTOME_CD22_MEDIATED_BCR_REGULATION
REACTOME_CREATION_OF_C4_AND_C2_ACTIVATORS
REACTOME_FCGR_ACTIVATION
REACTOME_FCGR3A_MEDIATED_IL10_SYNTHESIS
REACTOME_INITIAL_TRIGGERING_OF_COMPLEMENT
REACTOME_ROLE_OF_LAT2_NTAL_LAB_ON_CALCIUM_MOBILIZATION
REACTOME_ROLE_OF_PHOSPHOLIPIDS_IN_PHAGOCYTOSIS
REACTOME_SCAVENGING_OF_HEME_FROM_PLASMA
Up in SII
Up in SI & SIII
D
Cluster
Type
TIDE
Up
All
Down
Tumor
Normal
E
SI IPA
Transcriptional Regulation
Neurotransmitters and Other Nervous System Signaling
Biosynthesis
Xenobiotic Metabolism
Nuclear Receptor Signaling
Ingenuity Toxicity List Pathways
Cancer
Cardiovascular Signaling
Pathogen-Influenced Signaling
Degradation/Utilization/Assimilation
Organismal Growth and Development
Intracellular and Second Messenger Signaling
Cellular Stress and Injury
Cytokine Signaling
Apoptosis
Disease-Specific Pathways
Cellular Immune Response
Humoral Immune Response
Cellular Growth, Proliferation and Development
-log(p-value)
Color by z-score
Negative value
Positive value
Zero value
No activity pattern
Size by Number of genes that overlap the pathway
2
99
196
F
Inflammatory response
Chemotaxis of granulocytes
Chemotaxis of T lymphocytes
Chemotaxis of myeloid cells
Chemotaxis of neutrophils
Chemotaxis of lymphocytes
Chemotaxis of phagocytes
Stimulation of myeloid cells
Shape change of blood cells
Chemotaxis of leukocytes
Chemotaxis of mononuclear leukocytes
Stimulation of tumor cells
Shape change of leukocytes
Chemotaxis
IL18
IL1B
NFKB1
RNASE2
RNASE1
CAMP
IL17A
IL1A
CTLA4
G
SII IPA
Activation/Inactivation/Interconversion
Xenobiotic Metabolism
Growth Factor Signaling
Biosynthesis
Metabolic Clusters
Cell Cycle Regulation
Degradation/Utilization/Assimilation
Organismal Growth and Development
Nuclear Receptor Signaling
Neurotransmitters and Other Nervous System Signaling
Cardiovascular Signaling
Cancer
Pathogen-Influenced Signaling
Ingenuity Toxicity List Pathways
Intracellular and Second Messenger Signaling
Disease-Specific Pathways
Cellular Stress and Injury
Cytokine Signaling
Cellular Immune Response
Apoptosis
Humoral Immune Response
Cellular Growth, Proliferation and Development
-log(p-value)
Color by z-score
Negative value
Positive value
Zero value
No activity pattern
Size by Number of genes that overlap the pathway
2
94.5
187
H
TWIST2
LATS2
SFN
LATS1
Metastasis
Advanced stage tumor
Advanced malignant tumor
Predicted activation
Predicted inhibition
Predicted Relationships
Leads to activation
Leads to inhibition
Findings inconsistent with state of downstream molecule
Effect not predicted
Dashed lines = indirect relationship
Solid lines = direct relationship

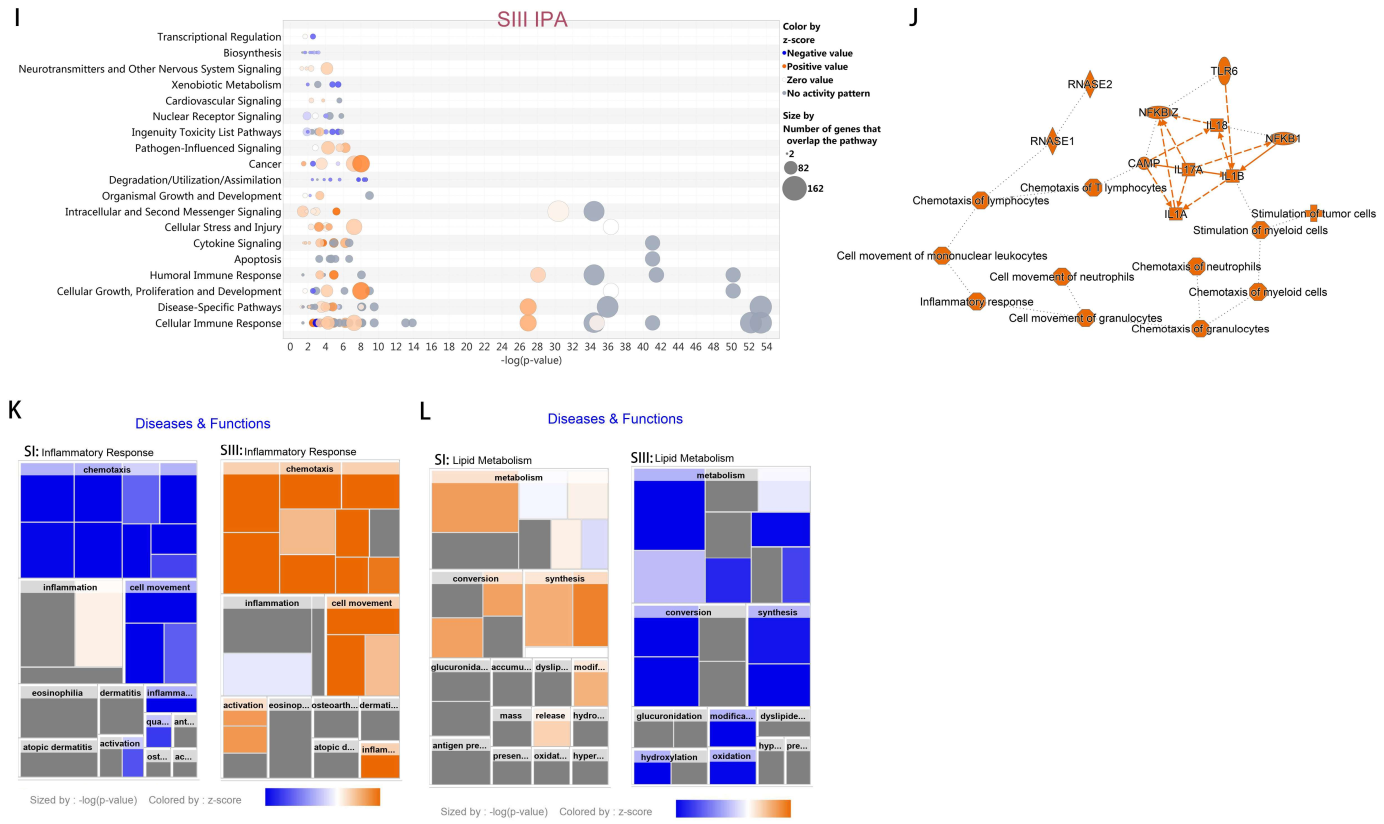

Figure S7. Signaling pathways and functional annotations of three TIDE subtypes of BC. **(A-C)** Gene set enrichment analysis (GSEA) shows the top 10 signaling pathways enriched in SI **(A)**, SII **(B)** and SIII **(C)**. **(D)** Gene set variation analysis (GSVA) reveals the top 10 signaling pathways enriched in the three TIDE subtypes. **(E, G, I)** Ingenuity pathways analysis (IPA) demonstrates the activation or inhibition status of canonical signaling pathways for SI **(E)**, SII **(G)** and SIII **(I)**. **(F, H, J)** Graphical summary explains biomolecular interactions and biological processes in SI **(F)**, SII **(H)** and SIII **(J)**. **(K, L)** Heatmap showing correlations of marker genes of three TIDE subtype with inflammatory response **(K)** and lipid metabolism **(L)**.

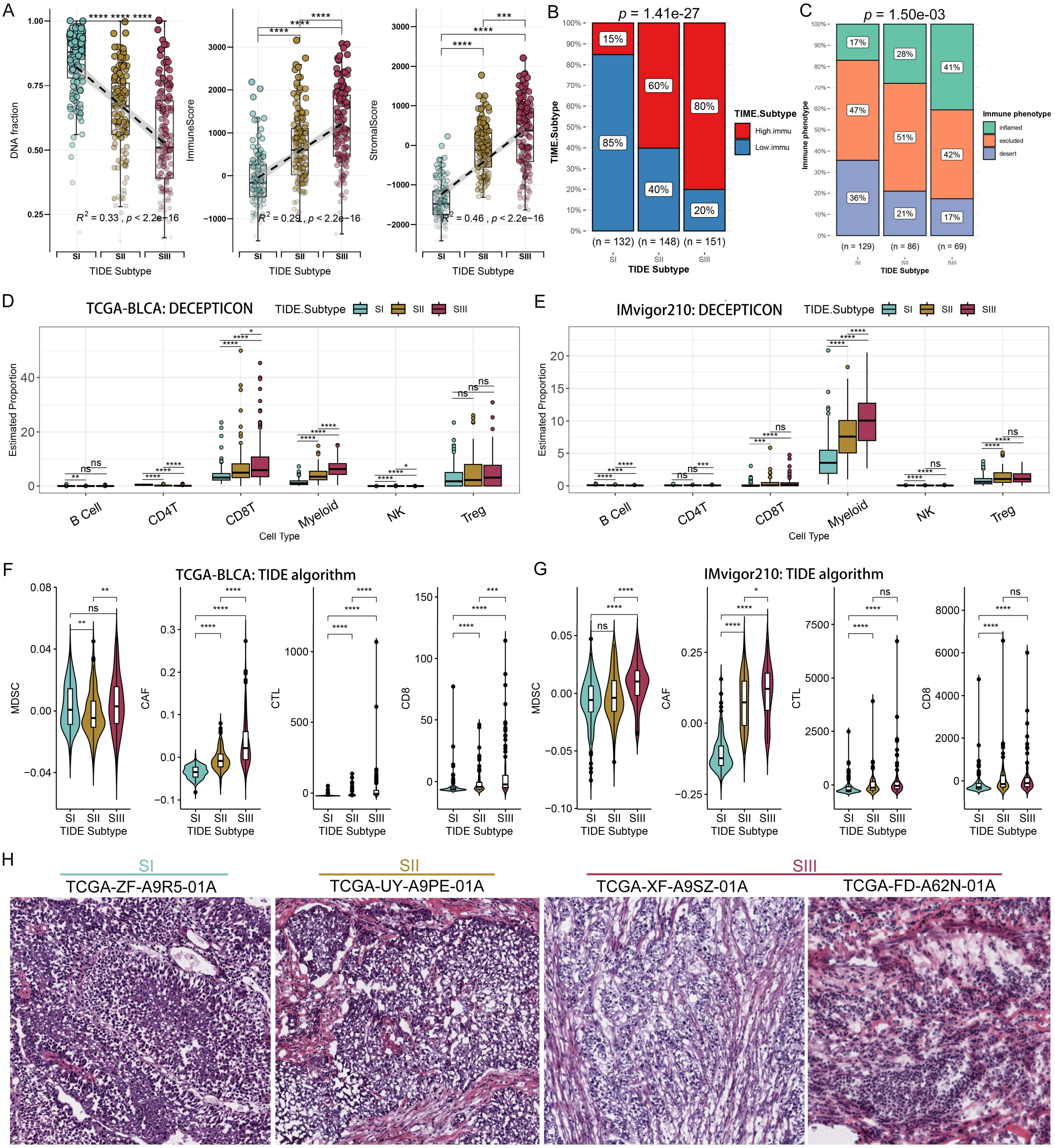

Figure S8. Characterizations of TME patterns among the TIDE subtypes based on bulk RNA-seq datasets (related to Figure 6). **(A)** Differences of DNA fraction, ImmuneScore, and StromalScore among the TIDE subtypes. **(B)** Stacked histogram showing the compositional differences of the TIME subtypes between the three TIDE subtypes of BC. **(C)** The proportion of immune phenotype among the TIDE subtypes of TCGA-BLCA. **(D, E)** Comparisons of immunocyte abundance among the three TIDE subtypes of TCGA-BLCA **(D)** and IMvigor210 **(E)**. Cell proportions are assessed by the DECEPTICON algorithm. **(F, G)** Comparison of the immunocyte proportion among three TIDE subtypes of TCGA-BLCA **(F)** and IMvigor210 **(G)**. Cell proportions are assessed by the TIDE algorithm. **(H)** Representative TCGA-BLCA H&E histological images of the three subtypes. *p < 0.05, **p < 0.01, ***p < 0.001, ****p < 0.0001; ns, no significance.

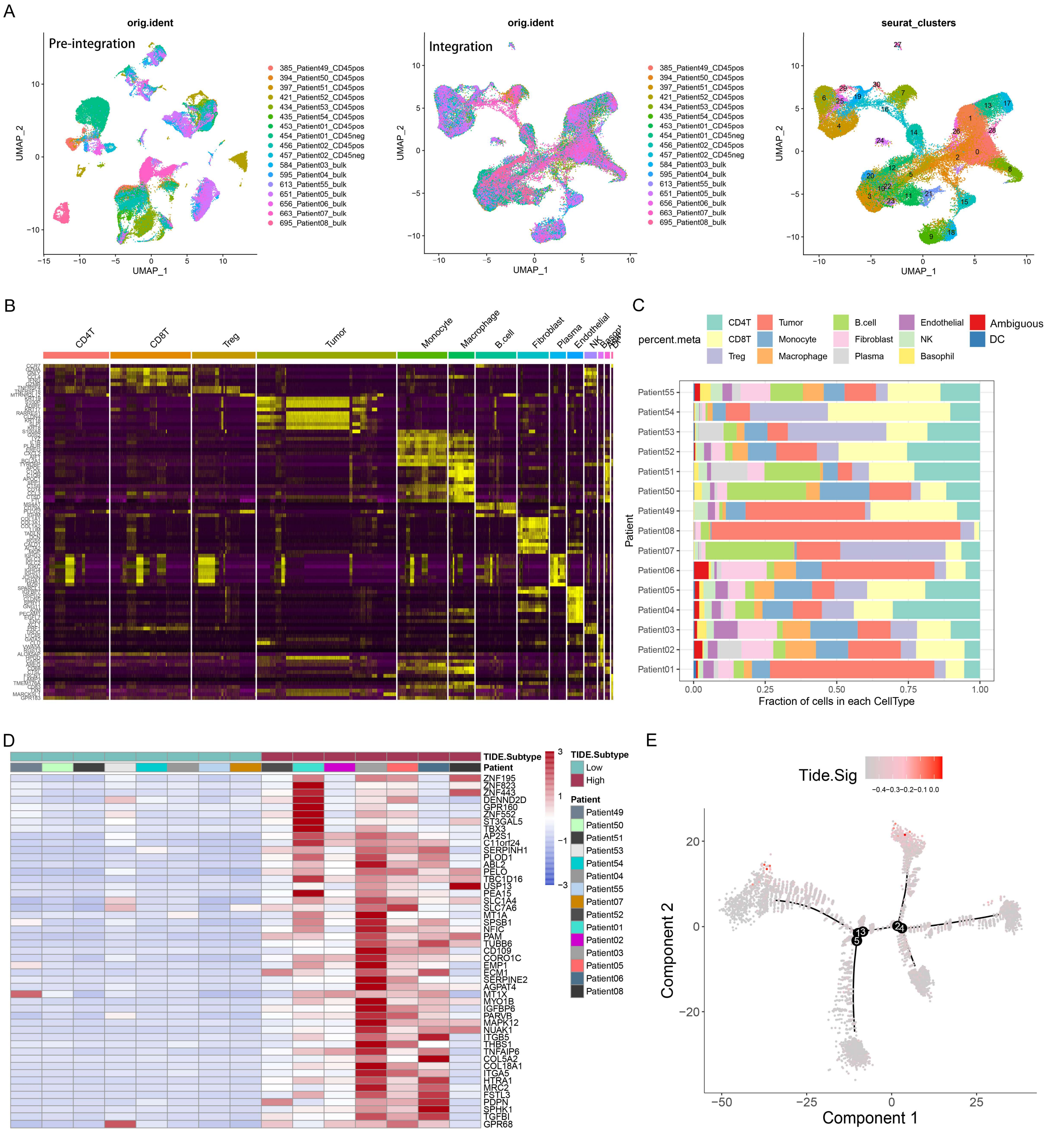

Figure S9. Characterizations of TME patterns among the TIDE subtypes based on single-cell RNA-seq dataset (related to Figure 7). **(A)** Uniform Manifold Approximation and Projection (UMAP) plot was used to analyze the single-cell RNA-seq dataset. Each color coded for the 17 pre-integrated samples **(left)**, 17 integrated samples **(middle)**, and 31 clusters **(right)** in Salomé's dataset. **(B)** Heatmap of top 10 marker gene expression of the 13 cell types. **(C)** Proportions of the 13 cell types in each sample. **(D)** Consensus clustering based on the expression of 69 TIDE marker genes classified pseudobulk data of Salomé's dataset into two subtypes: Low and High subtypes. **(E)** Expression levels of TIDE marker genes during the CD8T+ T cells (CD8Ts) differentiation trajectory in BC.

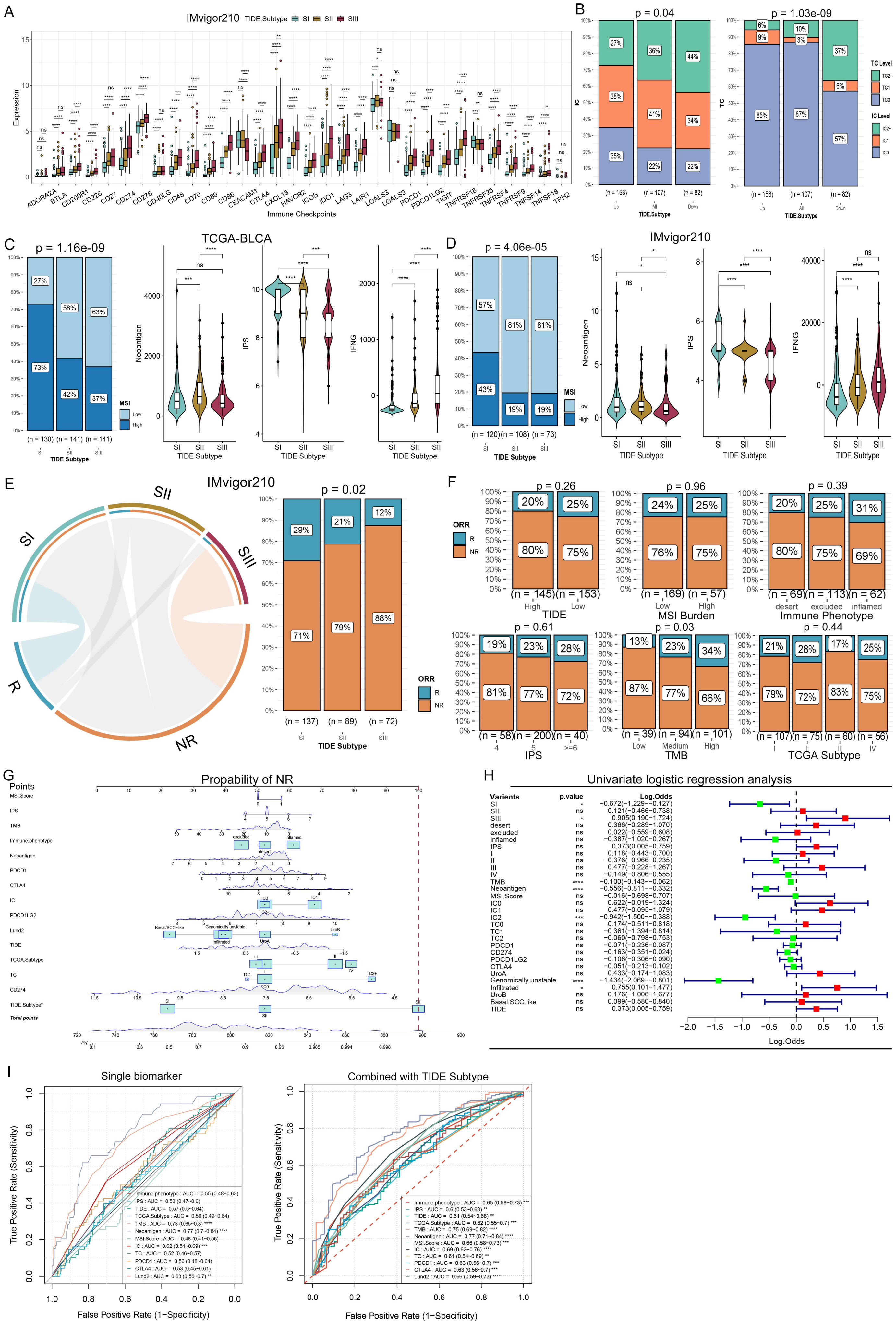
A
IMvigor210
TIDE.Subtype SI SII SIII
Expression
Immune Checkpoints
ADORA2A BTLA CD200R1 CD226 CD27 CD274 CD276 CD40LG CD48 CD70 CD80 CD86 CEACAM1 CTLA4 CXCL13 HAVCR2 ICOS IDO1 LAG3 LAIR1 LGALS3 LGALS9 PDCD1 PDCD1LG2 TIGIT TNFRSF18 TNFRSF25 TNFRSF4 TNFRSF9 TNFSF14 TNFSF18 TPH2
B
p = 0.04
p = 1.03e-09
IC
TC
TIDE.Subtype
Up All Down
TC Level TC2+ TC1 TC0
IC Level IC2+ IC1 IC0
C
p = 1.16e-09
TCGA-BLCA
Neoantigen
IPS
IFNG
MSI Low High
TIDE Subtype
D
p = 4.06e-05
IMvigor210
E
IMvigor210
SI SII SIII R NR
p = 0.02
ORR R NR
TIDE Subtype
F
p = 0.26 TIDE
p = 0.96 MSI Burden
p = 0.39 Immune Phenotype
p = 0.61 IPS
p = 0.03 TMB
p = 0.44 TCGA Subtype
G
Propability of NR
Points
MSI.Score IPS TMB Immune.phenotype Neoantigen PDCD1 CTLA4 IC PDCD1LG2 Lund2 TIDE TCGA.Subtype TC CD274 TIDE.Subtype*
Total points
H
Univariate logistic regression analysis
Varients p.value Log.Odds
SI * −0.672(−1.229~−0.127)
SII ns 0.121(−0.466~0.738)
SIII * 0.905(0.190~1.724)
desert ns 0.366(−0.289~1.070)
excluded ns 0.022(−0.559~0.608)
inflamed ns −0.387(−1.020~0.267)
IPS ns 0.373(0.005~0.759)
I ns 0.118(−0.443~0.700)
II ns −0.376(−0.966~0.235)
III ns 0.477(−0.228~1.267)
IV ns −0.149(−0.806~0.555)
TMB **** −0.100(−0.143~−0.062)
Neoantigen **** −0.556(−0.811~−0.332)
MSI.Score ns −0.016(−0.698~0.707)
IC0 ns 0.622(−0.019~1.324)
IC1 ns 0.477(−0.095~1.079)
IC2 *** −0.942(−1.500~−0.388)
TC0 ns 0.174(−0.511~0.818)
TC1 ns −0.361(−1.394~0.814)
TC2 ns −0.060(−0.798~0.753)
PDCD1 ns −0.071(−0.236~0.087)
CD274 ns −0.163(−0.351~0.024)
PDCD1LG2 ns −0.106(−0.306~0.090)
CTLA4 ns −0.051(−0.213~0.102)
UroA ns 0.433(−0.174~1.083)
Genomically.unstable **** −1.434(−2.069~−0.801)
Infiltrated * 0.755(0.101~1.477)
UroB ns 0.176(−1.006~1.677)
Basal.SCC.like ns 0.099(−0.580~0.840)
TIDE ns 0.373(0.005~0.759)
Log.Odds
I
Single biomarker
Combined with TIDE Subtype
True Positive Rate (Sensitivity)
False Positive Rate (1−Specificity)

Figure S10. TIDE subtypes were closely related to ICB response (related to Figure 8). **(A)** Comparisons of the immune checkpoint molecules expression levels among the TIDE subtypes of IMvigor210 dataset. **(B)** Proportions of IC and TC levels in the TIDE subtypes. **(C, D)** Comparisons of MSI, neoantigen load, IPS and interferon gamma among the TIDE subtypes of TCGA-BLCA **(C)** and IMvigor210 dataset **(D)**. **(E)** Hypergeometric test revealed an association between TIDE subtypes of IMvigor210 and ICB responses **(left)**, gray lines represent no significance; Stacked histogram showing the differences of ICB responses among the TIDE subtypes of IMvigor210 dataset **(right)**. **(F)** The relationship of common biomarkers (TIDE score, MSI, immune phenotype, IPS, TMB, TCGA subtype) with ICB responses in the IMvigor210 dataset. **(G)** Nomogram for predicting ICB non-responders based on logistic regression model. Red line showing the SIII points. **(H)** Impacts of the TIDE subtypes and the predictive biomarkers on ICB efficacy, which was achieved by univariate logistic regression analysis. **(I)** ROC curves of single biomarkers **(left)** and TIDE subtypes+ other biomarkers **(right)** for predicting the ICB efficacy. R, response; NR, non-response. *$p < 0.05$, **$p < 0.01$, ***$p < 0.001$, ****$p < 0.0001$; ns, no significance.

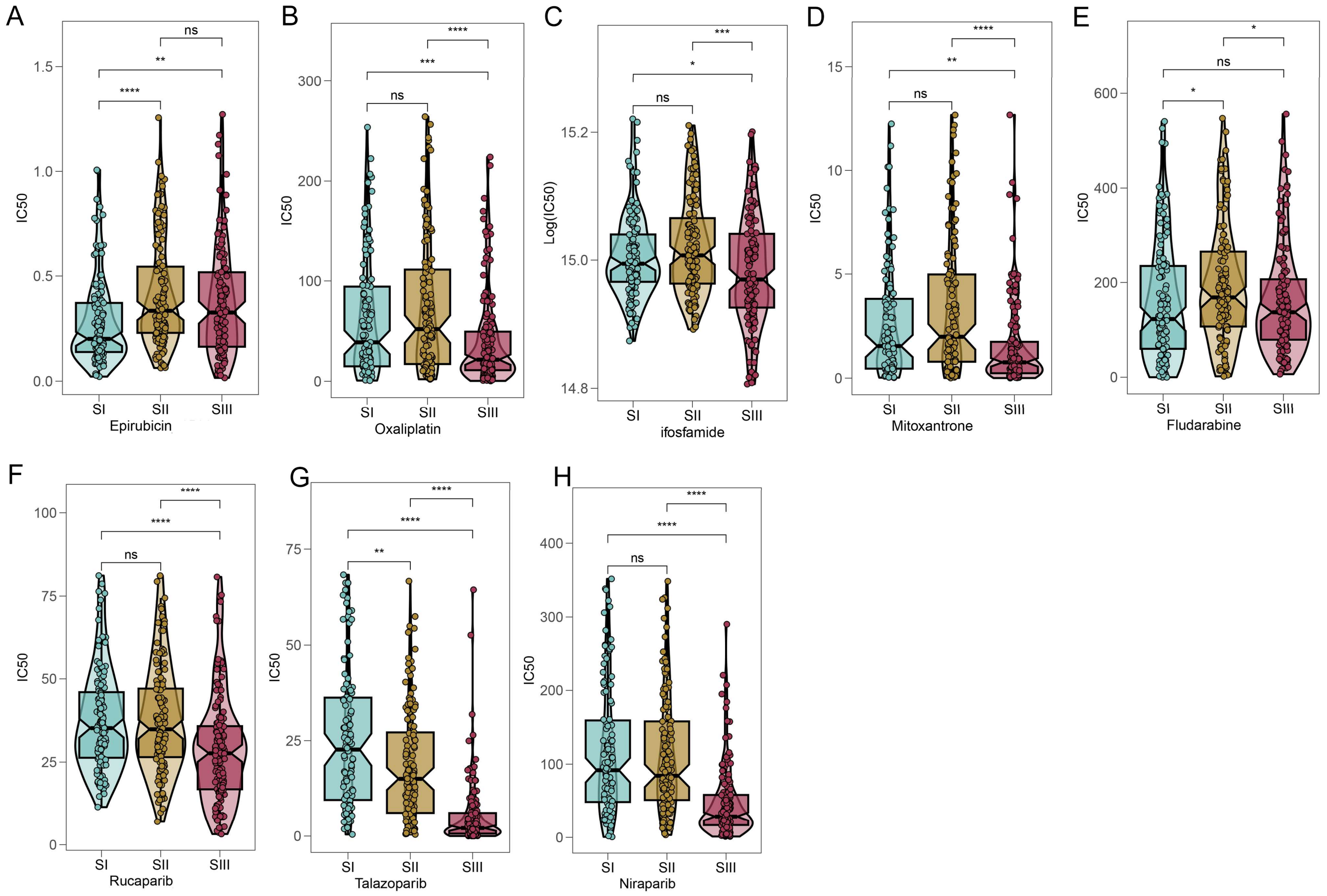

Figure S11. Comparisons of drug sensitivities and identification of the potential targeted compounds of BC TIDE subtypes (related to Figure 9). **(A-H)** Comparisons of sensitivities of the TIDE subtypes to clinically recommended drugs: epirubicin **(A)**, oxaliplatin **(B)**, ifosfamide **(C)**, mitoxantrone **(D)**, fludarabine **(E)**, rucaparib **(F)**, talazoparib **(G)**, and niraparib **(H)**. *p < 0.05, **p < 0.01, ***p < 0.001, ****p < 0.0001; ns, no significance.

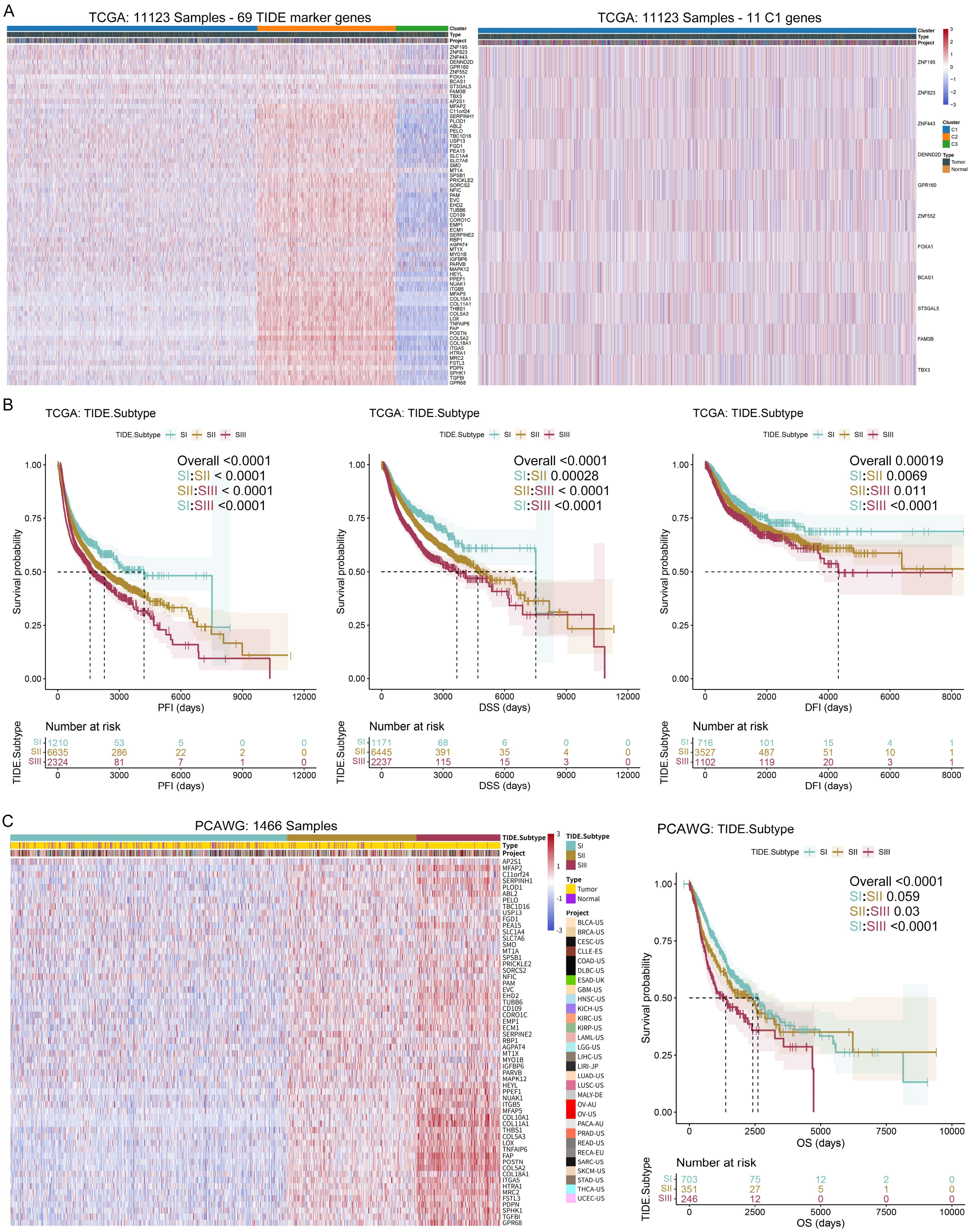
A
TCGA: 11123 Samples - 69 TIDE marker genes
Cluster
Type
Project
ZNF195 ZNF823 ZNF443 DENND2D GPR160 ZNF552 FOXA1 BCAS1 ST3GAL5 FAM3B TBX3 AP2S1 MFAP2 C11orf24 SERPINH1 PLOD1 ABL2 PELO TBC1D16 USP13 FGD1 PEA15 SLC1A4 SLC7A6 SMO MT1A SPSB1 PRICKLE2 SORCS2 NFIC PAM EVC EHD2 TUBB6 CD109 CORO1C EMP1 ECM1 SERPINE2 RBP1 AGPAT4 MT1X MYO1B IGFBP6 PARVB MAPK12 HEYL PPEF1 NUAK1 ITGB5 MFAP5 COL10A1 COL11A1 THBS1 COL5A3 LOX TNFAIP6 FAP POSTN COL5A2 COL18A1 ITGA5 HTRA1 MRC2 FSTL3 PDPN SPHK1 TGFBI GPR68
TCGA: 11123 Samples - 11 C1 genes
Cluster
Type
Project
ZNF195
ZNF823
ZNF443
DENND2D
GPR160
ZNF552
FOXA1
BCAS1
ST3GAL5
FAM3B
TBX3
Cluster C1 C2 C3
Type Tumor Normal
B
TCGA: TIDE.Subtype
TIDE.Subtype SI SII SIII
Overall <0.0001
SI:SII < 0.0001
SII:SIII < 0.0001
SI:SIII <0.0001
Survival probability
PFI (days)
Number at risk
SI 1210 53 5 0 0
SII 6635 286 22 2 0
SIII 2324 81 7 1 0
TCGA: TIDE.Subtype
Overall <0.0001
SI:SII 0.00028
SII:SIII < 0.0001
SI:SIII <0.0001
DSS (days)
Number at risk
SI 1171 68 6 0 0
SII 6445 391 35 4 0
SIII 2237 115 15 3 0
TCGA: TIDE.Subtype
Overall 0.00019
SI:SII 0.0069
SII:SIII 0.011
SI:SIII <0.0001
DFI (days)
Number at risk
SI 716 101 15 4 1
SII 3527 487 51 10 1
SIII 1102 119 20 3 1
C
PCAWG: 1466 Samples
TIDE.Subtype
Type
Project
AP2S1 MFAP2 C11orf24 SERPINH1 PLOD1 ABL2 PELO TBC1D16 USP13 FGD1 PEA15 SLC1A4 SLC7A6 SMO MT1A SPSB1 PRICKLE2 SORCS2 NFIC PAM EVC EHD2 TUBB6 CD109 CORO1C EMP1 SERPINE2 RBP1 AGPAT4 MT1X MYO1B IGFBP6 PARVB MAPK12 HEYL PPEF1 NUAK1 ITGB5 MFAP5 COL10A1 COL11A1 THBS1 COL5A3 LOX TNFAIP6 FAP POSTN COL5A2 COL18A1 ITGA5 HTRA1 MRC2 FSTL3 PDPN SPHK1 TGFBI GPR68
TIDE.Subtype SI SII SIII
Type Tumor Normal
Project BLCA-US BRCA-US CESC-US CLLE-ES COAD-US DLBC-US ESAD-UK GBM-US HNSC-US KICH-US KIRC-US KIRP-US LAML-US LGG-US LIHC-US LIRI-JP LUAD-US LUSC-US MALY-DE OV-AU OV-US PACA-AU PRAD-US READ-US RECA-EU SARC-US SKCM-US STAD-US THCA-US UCEC-US
PCAWG: TIDE.Subtype
TIDE.Subtype SI SII SIII
Overall <0.0001
SI:SII 0.059
SII:SIII 0.03
SI:SIII <0.0001
Survival probability
OS (days)
Number at risk
SI 703 75 12 2 0
SII 351 27 5 1 0
SIII 246 12 0 0 0

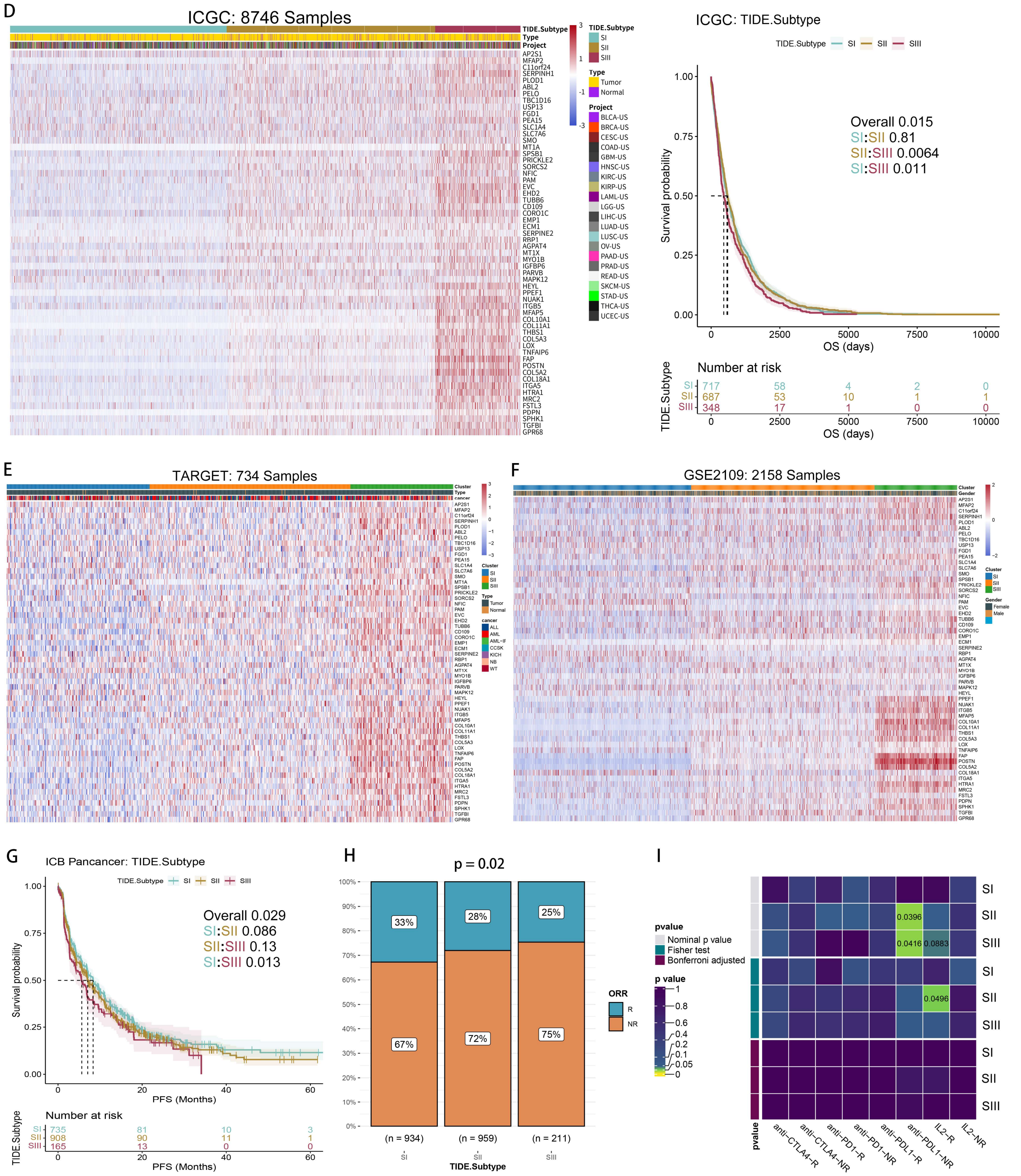

Figure S12. Conservations of the TIDE subtypes in pan-tumors (related to Figure 10). **(A)** Unsupervised hierarchical clustering based on the 69 TIDE marker genes classified pan-tumor samples from TCGA into three subtypes.11 C1 genes were approximately equally expressed among the TIDE subtypes **(left)**. Unsupervised clustering analysis using the 11 C1 genes failed to cluster the pan-cancer samples **(right). (B)** K-M analysis showing significant differences in PFI, DSS and DFI among the TIDE subtypes of TCGA pan-tumors. **(C-F)** Unsupervised hierarchical clustering based on the 58 C2 genes classified pan-tumor samples from PCAWG **(C)**, ICGC **(D)**, TARGET **(E)** and GSE2109 **(F)** into three TIDE subtypes. K-M analysis shows distinct prognosis of the TIDE subtypes of PCAWG **(C-right)** and ICGC **(D-right)**. **(G)** K-M analysis shows distinct prognosis of the TIDE subtypes in the ICB-treated pan-tumor cohort. **(H)** Stacked histogram showing the differences of pan-tumor ICB responses among the TIDE subtypes. **(I)** Submap analysis showing the sensitivity of the pan-tumor TIDE subtypes to ICB therapy.

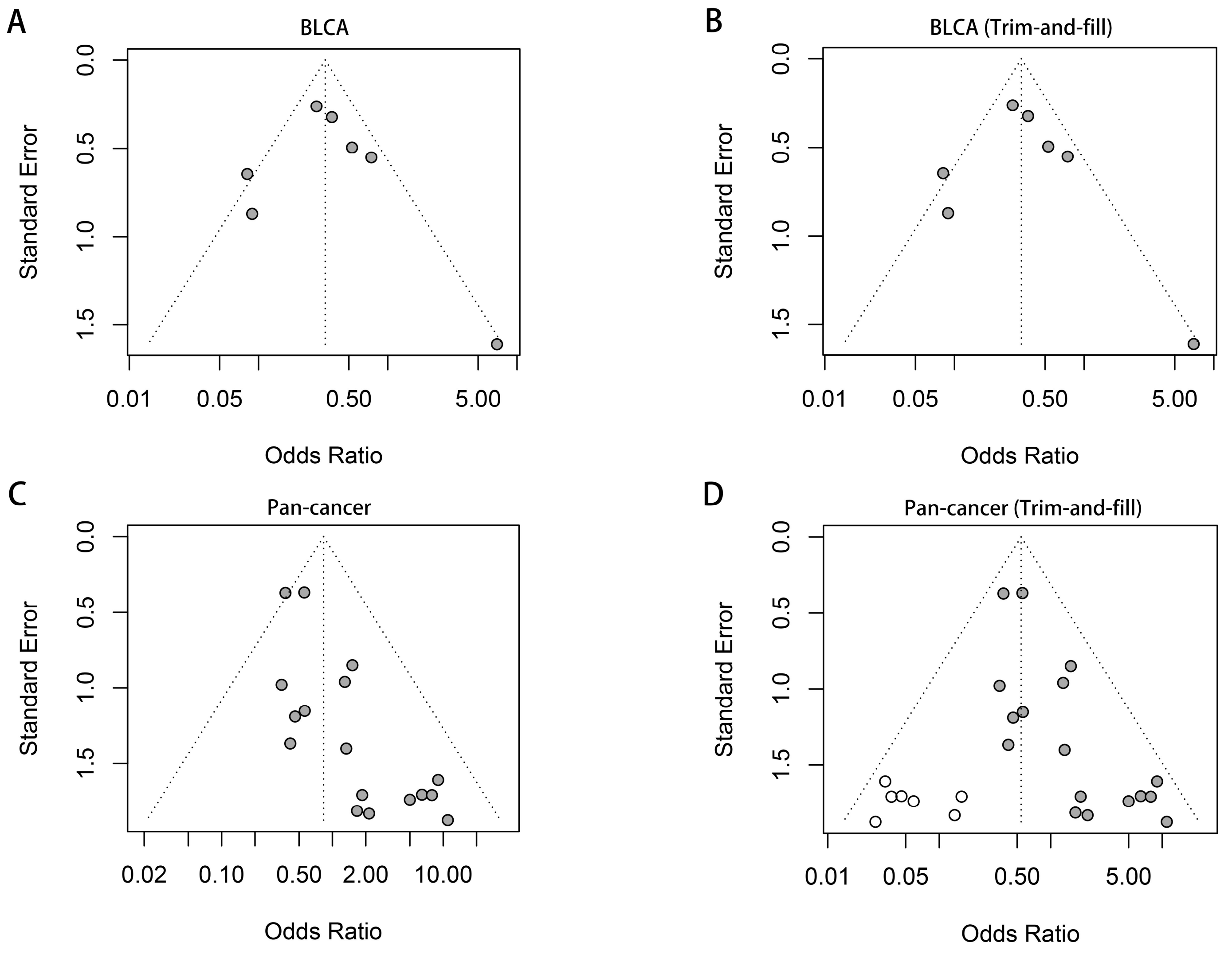

Figure S13. Funnel plots to evaluate the publication bias of TIDE-based subtypes regarding the overall survival of both BC and Pan-cancer datasets. (A-B) For the BC datasets (n = 7), the Egger test indicated no publication bias (p = 0.7689), and the funnel plot did not reveal significant asymmetry (**A**). Meanwhile, no indications of studies identified according to the trim and fill analysis (**B**). **(C-D)** For the Pan-cancer datasets (n = 17), the Peters test indicated publication bias (p = 0.0011), and the funnel plot exhibited asymmetry (**C**). After conducting the trim and fill analysis, seven putative missing studies were identified on the left side of the distribution (**D**), and the adjusted result of the Peters test revealed no publication bias (p = 0.6037).

Methods S1

Study Design

The design of this study is as follows:

(1) Evaluation of tumor immune dysfunction and exclusion (TIDE) status and its relationship with clinicopathological and molecular features of bladder cancer (BC): We used the Tumor Immune Dysfunction and Exclusion (TIDE) algorithm[40] to evaluate the TIDE status and calculate the TIDE scores of samples in eight bulk RNA-seq datasets of BC[1-5]. Then, we explored the association between TIDE scores and clinicopathological features (such as pathological TNM staging, age, weight, race, gender, and follow-up information) and molecular features (such as tumor mutational burden [TMB], somatic copy number alterations) of BC.

(2) TIDE subtyping of BC: First, we identified 69 TIDE marker genes through correlation analysis and univariate COX analysis. Then, we divided BC patients into three subtypes using unsupervised consensus clustering[41] based on these TIDE marker genes.

(3) Characterization of clinicopathological and molecular features of TIDE subtypes: First, we used two additional algorithms - Unsupervised hierarchical clustering and Non-negative Matrix Factorization (NMF)[42] - based on 69 TIDE marker genes to classify BC samples, to test the stability of the subtypes. Next, we characterized the differences in clinical and pathological features, molecular features, tumor immune microenvironment (TIME), functional annotations and signaling pathways, as well as drug sensitivity of three TIDE subtypes.

(4) Analysis of the pan-tumor landscape of the TIDE subtypes: First, we performed unsupervised hierarchical clustering on five pan-tumor datasets based on 69 TIDE marker genes, dividing each dataset into three TIDE subtypes. Next, we validated the sensitivity differences of the three TIDE subtypes to immune checkpoint blockade (ICB) therapy using a bulk RNA-seq pan-tumor cohort of baseline samples treated with ICB.

Datasets collection

Five bulk RNA-seq datasets[1-5] and one scRNA-seq dataset[39] of BC, five bulk-RNA-seq cohorts of pan-tumors, one bulk RNA-seq cohort of pan-tumors treated with ICB[6-38], and somatic mutation and CNA data from TCGA-BLCA were collected in this study. Bulk RNA-seq datasets of BC and follow-up information of TCGA-BLCA were collected from TCGA, Gene Expression Omnibus (GEO) and UCSC Xena. The CNA and somatic mutation data were downloaded from TCGA using the TCGAbiolinks package (v2.25.3). Single-cell RNA-seq dataset of BC was collected from the Mendeley Data (https://data.mendeley.com/datasets/7yb7s9769c/1). Pan-cancer cohorts were collected from UCSC Xena and GEO. The pretreatment bulk RNA-seq cohort of pan-tumors treated with ICB was obtained from multiple databases (**Table S1**). This cohort included 2641 samples from 36 datasets of 12 tumor types. The database of MSigDB[43] (v2023.1.Hs, http://www.gsea-msigdb.org/gsea/index.jsp) and STRING[44] (v11.5, https://cn.string-db.org/) were also collected in this study. Details and sources for all datasets are listed in **Table S1**.

Real-world bladder tumor samples collection

After obtaining patient consent and approval from the institutional research ethics committee, we collected 51 surgical resection samples of bladder tumors from 31 patients who hospitalized in the Department of Urology, Shanghai Sixth People's Hospital. **Table S2** lists detailed information of the patients. For all resected tumor samples, we invited pathology experts to review the samples for accurate pathological diagnosis of the patients. All tumor samples were collected within 5 minutes after excision and immediately placed in liquid nitrogen for preservation. Subsequently, they were operated for bulk RNA sequencing (LY dataset) to validate the TIDE subtypes. Bulk RNA-seq data have been uploaded to GEO and are available through accession number GSE248167.

Bulk RNA sequencing and processing

RNA quality was measured with Bioanalyzer 2100 system and RNA Nano 6000 Assay Kit. mRNA was purified from total RNA with magnetic beads with poly-T oligos. mRNA was fragmented with divalent cations and high temperature. First and second strand cDNA were made with random primers, M-MuLV Reverse Transcriptase (RNase H-), DNA Polymerase I and RNase H. cDNA ends were polished and adenylated. Adaptor with hairpin loop structure was ligated to cDNA. cDNA fragments of about 370~420 bp were selected with AMPure XP system. PCR was done with Phusion High-Fidelity DNA polymerase, Universal PCR primers and Index (X) Primer. PCR products were cleaned with AMPure XP system and library quality was checked with Agilent Bioanalyzer 2100 system. Index-coded samples were clustered with cBot Cluster Generation System and TruSeq PE Cluster Kit v3-cBot-HS (Illumia). Libraries were sequenced on Illumina Novaseq platform and 150 bp paired-end reads were obtained. Raw data of fastq format were processed with fastp software. Reads with adapter, ploy-N and low quality were removed. Clean data were obtained and Q20, Q30 and GC content were calculated. Reference genome and gene model annotation files were downloaded from genome website. Reference genome index was built with Hisat2 v2.0.5. Clean reads were aligned to reference genome with Hisat2 v2.0.5. Gene model annotation file was used by Hisat2 to make splice junctions database and get better mapping result. Mapped reads were assembled in reference-based way with StringTie (v1.3.3b). StringTie used new network flow algorithm and optional de novo assembly step to make and quantify full-length transcripts for each gene locus. Reads numbers for each gene were counted with featureCounts v1.5.0-p3. FPKM of each gene was calculated based on gene length and reads count.

Single-cell RNA-seq dataset processing

Single-cell RNA-seq dataset was processed using uniform methods and standards with the Seurat package[45] (v4.2.1). Cells with less than 200 genes or more than 20% mitochondrial genes were removed, as well as features detected in less than 3 cells. Normalization was performed using Seurat's defaults and 2000 hypervariable features were selected for downstream analysis. The sample integration and batch effect removal were performed using the anchor-based canonical correlation analysis (CCA) method, followed by data normalization using the ScaleData function. The integrated RNA assay was used for dimensionality reduction and clustering, and principal component analysis (PCA) was performed using the RunPCA function. Louvain clustering was done

with 30 PCs and resolution = 1, and the results were visualized in two-dimensional space using the uniform manifold approximation and projection (UMAP). To identify marker genes for each cluster, differential expression (DE) analysis was conducted using the FindAllMarkers function. SingleR package[46] (v2.0.0) was used to annotate all clusters with relevant marker genes reported in the literature. Single-cell pseudotime and trajectory analysis was implemented with the Monocle 2 package (v2.22.0)[47].

Differential expression (DE) analysis

Marker genes for each Louvain cluster were identified using the FindAllMarkers function[45]. For bulk RNA-seq datasets, DE analysis was performed using the scCODE package (v1.2.0.1)[48]. Genes with Bonferroni FDR-corrected p-values < 0.05 and Detected_Times ⩾ 3 were considered as DE genes.

Tumor immune dysfunction (TID) and tumor immune exclusion (TIE) analysis

The computational framework developed by Liu et al., called Tumor Immune Dysfunction Exclusion (TIDE), is used to evaluate the TID and TIE status of cancer patients[40]. This algorithm evaluates TIDE scores based on two cancer immune escape mechanisms: the promotion of T cell dysfunction in tumors exhibiting significant infiltration of cytotoxic T lymphocytes (CTL), and the inhibition of T cell infiltration in tumors characterized by a low level of CTL[40]. Higher TIDE scores indicate greater TID and TIE levels. We used the TIDE package (v1.3) in Python software to assign TIDE score for each sample.

In the K-M analysis, the optimal cutoff point was calculated using the built-in surv_cutpoint function in the Survminer package (v0.4.9) to divide the patients into high and low TIDE groups.

Protein–protein interaction network analysis (PPI)

PPI network of TIDE marker genes were constructed using the STRING database[44] (https://cn.string-db.org/).

Clustering analysis

Consensus clustering was carried out using the ConsensusClusterPlus package[41] (v1.58.0). To improve the reliability of our clustering, we repeated the process 1000 times, randomly selecting 80% of the samples each time. To ensure the accuracy of our classification results, we employed two additional techniques: unsupervised hierarchical clustering and non-negative matrix factorization (NMF). When performing unsupervised hierarchical clustering, we applied hclust to the normalized data using default parameters, which perform agglomerative clustering using the complete linkage method. We then visualized the results using a dendrogram, which represents the hierarchical structure of the clusters. To specify a desired number of clusters, we used the cutree function to cut the dendrogram. Meanwhile, we employed the NMF algorithm from the NMF

package[42] (v0.24.0), which decomposes the matrix and performs 100 repetitions for stable, unsupervised clustering.

The optimal number of clusters was comprehensively determined by the consensus heatmap, the cumulative distribution function (CDF) curves, and the proportion of ambiguous clustering algorithm (PAC)[49] (**Figure S5A**).

Signaling pathway analysis

Download the reference gene-sets for GSEA analysis, including GOBP, Hallmark, KEGG, and Reactome, from the Molecular Signatures Database[43] (MSigDB, http://www.gsea-msigdb.org/gsea/index.jsp). The Investigate Gene Sets tool (http://www.gsea-msigdb.org/gsea/msigdb/annotate.jsp) can be used to identify the overlapping gene-sets of the submitted gene-lists in MSigDB.

Use the "ssgsea" method of the GSVA package[50] (v1.44.5) to perform single-sample gene set enrichment analysis (ssGSEA) and assign corresponding signature activity scores to each sample.

Evaluate the enrichment scores of signaling pathways using gene set variation analysis (GSVA) with the "gsva" method of the GSVA package[50]. Use the Limma package[51] to perform differential analysis on the enrichment scores, which can identify pathways significantly enriched in different TIDE subtypes.

Perform gene set enrichment analysis (GSEA) on pre-ranked DE gene-lists using the clusterProfiler package[52] (v4.4.4), with reference to the GOBP, Hallmark, KEGG, and Reactome gene-sets.

Use the QIAGEN IPA software (IPA Winter Release December 2022, https://digitalinsights.qiagen.com/products-overview/discovery-insights-portfolio/analysis-and-visualization/qiagen-ipa/) to perform ingenuity pathway analysis (IPA) on identified DE genes of each TIDE subtype. The Pathways tool can expose the activation or inhibition status of canonical pathways in each subtype. The Disease and Function tool can reveal the correlation between the interested gene sets and biological functions or diseases.

Somatic mutation and CNV analysis

We analyzed genomic characteristics and mutation spectrum of the TIDE subtypes by performing somatic mutation and CNV analyses. Somatic mutation data and CNV data of TCGA-BLCA were downloaded, followed by the identification of significant amplified or deleted genomic regions using GISTIC_2.0 (v6.15.30, https://cloud.genepattern.org/gp/pages/index.jsf). The G-score for each region was calculated for the amplitude and frequency of variations. Mutation types, frequencies, and CNVs were further analyzed and visualized using maftools package (v2.12.0)[53].

Survival Analysis

We performed survival analysis using the Survival package (v3.4.0) and the Survminer package (v0.4.9) based on the Kaplan-Meier method, and univariate and multivariate Cox regression

analyses. The survival curve was plotted by the Kaplan-Meier method, and the log-rank test was applied to compare survival differences among the TIDE subgroups.

Bias Analysis

The Egger's test and Peter's test were employed to assess publication bias in the bladder cancer datasets ($n = 7$, < 10) and the pan-cancer datasets ($n = 17$, ≥ 10), respectively. The trim-and-fill analysis was conducted to rectify the biased results.

Evaluation of tumor immune microenvironment (TIME) patterns

We applied 54 immune-related signatures from Charoentong et al[54]. and Şenbabaoğlu et al[55]. to examine the TIME patterns of BC samples. The ssGSEA algorithm[50] was used to compute the active scores of these signatures. Consensus clustering[41] of the ssGSEA scores were used to classify BC samples into different immune subtypes. We also used the ESTIMATE package (v1.0.13)[56] to analyze the TME status using the transcriptome data. This package can estimate the levels of stromal cells, immune infiltration, and tumor purity. Moreover, we used the SCDC[57] and DECEPTICON (v1.0, https://github.com/Hao-Zou-lab/DECEPTICON) algorithms to evaluate the abundance of immunocytes in the tissue samples.

Evaluation of immunotherapy and targeted therapeutic efficacy

To evaluate the immunotherapy and targeted treatment responses among different TIDE subtypes, we employed unsupervised subclass mapping analysis[58] (SubMap, v4.0, https://cloud.genepattern.org/gp/pages/index.jsf). Submap is an unsupervised machine learning method to measure the similarity between TIDE subtypes and therapeutic efficacy, which helps us to determine the sensitivity to immunotherapy and targeted treatment in different groups[58]. Furthermore, we also validated this finding by analyzing a dataset of pan-tumors received ICB treatment.

Evaluation of drug sensitivity

The oncoPredict package[59] (v0.2) was used to evaluate drug sensitivity of TIDE subtypes using GDSC[60] (v8.4, https://www.cancerrxgene.org/) and CTRP[61] (v2, https://portals.broadinstitute.org/ctrp/) databases. The half-maximal inhibitory concentration (IC50) was used to measure drug sensitivity. The IC50 of drugs in BC patients was computed using ridge regression algorithm[59].

Identification of TIDE marker genes for TIDE subtyping

We identified TIDE marker genes based on 8 bulk RNA-seq datasets of BC[1-6]. Spearman correlation analysis was performed between gene expression levels and TIDE scores of the datasets. The selected genes were significantly correlated with TIDE scores ($FDR < 0.05$) called TIDE.Genes. Calculate the arithmetic mean of Spearman's R for each gene in the collected datasets. Genes were

merged as TIDE.Sig if they were significantly correlated with TIDE score in at least four datasets and an average R not less than 0.3. The univariate COX analysis was performed on TIDE.Sig based on the survival data, and genes that were significantly associated with prognosis ($p < 0.05$) in at least four datasets were selected as TIDE marker genes.

Hematoxylin-eosin (H&E) and immunohistochemistry (IHC) staining

The collected BC tissue specimens were fixed in formalin and embedded in paraffin in the Pathology Department of the Shanghai Sixth People's Hospital. BC sections were cut from paraffin-embedded tissue blocks, soaked with 10% formaldehyde, and fixed on slides. The slides were incubated at 42°C overnight. For pathological evaluation, all sections were stained with H&E staining. For IHC analyses, sections were deparaffinized and rehydrated followed with antigen retrieval and serum blocking. They were incubated with primary antibodies at 4°C overnight, secondary antibodies at 37°C for 1 hour and Strept Avidin-Biotin Complex (SABC) at 37°C for 30 min. All sections were colored with DAB and countercolored with hematoxylin. The primary antibodies used including the Anti-MKI67 antibody (1:200, C650056-0100, Sangon Biotech) and Anti-HER2 antibody (1:200, D199358-0100, Sangon Biotech).

Enzyme-linked immunosorbent assay (ELISA)

Fasting morning urine samples were collected from the enrolled 31 patients. The urine samples were centrifuged, and the supernatants were used for ELISA. The procedure was according to the manufacturer's instruction. Firstly, add 100 μL of sample diluent (blank group), cytokine standards (standard curve group) and urine samples (experimental group) to a 96-well plate. Add 50 μL of enzyme-labeled antibody to each well and incubate at 37°C for 60 minutes. Wash the plate three times with washing buffer, each time for 5 minutes, to remove excess enzyme-labeled antibody. Add 50 μL of chromogen to each well and incubate in the dark at room temperature for 15 minutes. Finally, 50 μL of stop solution was used to stop the color development. Measure the optical density (OD) value at 450 nm with an enzyme-labeled instrument. Plot the standard curve with the OD values, and obtain the equation and correlation coefficient. Calculate the cytokine concentrations of the samples with the standard curve equation. The ELISA kits were used including Human IL-6 ELISA Kit (D711391, Sangon Biotech), Human IL-8 ELISA Kit (D711366, Sangon Biotech), Human IL-4 ELISA Kit (D711052, Sangon Biotech), Human IL-10 ELISA Kit (D711393, Sangon Biotech), Human IFN-γ (Interferon Gamma) ELISA Kit (D711044, Sangon Biotech), Human IL-12 (Interleukin 12) ELISA Kit (D711069, Sangon Biotech), Human TNF-α ELISA Kit (D711045, Sangon Biotech).

Immunofluorescent (IF) staining

For IF staining, dewax the paraffin sections with xylene, absolute ethanol, 85% alcohol, 75% alcohol and distilled water in sequence. Perform antigen retrieval with EDTA antigen retrieval buffer (pH = 8.0) in a microwave oven. Medium heat for 8 minutes, stop for 8 minutes, and medium-low heat for 7 minutes. After cooling, wash with PBS (pH = 7.4) for 3×5 minutes. Draw a circle with a tissue marker around the tissue, add self-fluorescence quenching agent for 5 minutes, and rinse with

running water for 10 minutes. Add BSA solution to seal the sections for 30 minutes. Shake off the sealing liquid and add the first primary antibody at 4°C overnight. Wash the slides with PBS for 3×5 minutes and add the HRP-labeled second antibody. Cover the tissue and incubate for 50 minutes at room temperature. Following PBS wash for 3×5 min, add CY3-TSA solution with incubation for 10 min at room temperature, and wash with TBST for 3×5 min. For double IF staining, repeat microwave treatment to remove the first primary and secondary antibodies. After BSA blocking buffer treatment, add the second primary antibody, HRP-labeled second antibody, and incubate with FITC-TSA for 10 min at room temperature. For triple IF staining, slides were subsequently treated with microwave again. After BSA buffer incubation, add the third primary antibody, HRP-labeled second antibody, and incubate in the CY5-TSA buffer in the dark for 10 min. The primary antibodies of double IF included FAP antibody (1:300, PA5-32765, Thermo Fisher Scientific) and CD45 antibody (1:1000, GB113886, Servicebio). The primary antibodies of triple IF included PD-1 antibody (1:200, GB12338, Servicebio), PD-L1 (CD274) antibody (1:200, A20344, ABclonal Technology) and CTLA-4 antibody (1:200, A2063, ABclonal Technology). Finally, the slides were stained with DAPI and imaged by confocal microscopy. Images analyses were carried out with ImageJ (v2) software.

CT-based calculation of tumor volume

For mass-like tumors, we approximate them as half an ellipsoid, and we need to measure the long and short diameters of the largest cross-section of the tumors, count the number of layers where the tumor appears and record the CT slice thickness, the volume formula is:

$$\text{Volume} = \frac{\pi}{6} * L * S * H,$$

$$\text{H} = \text{number of layers} * \text{slice thickness},$$

where L is the long diameter, S is the short diameter, and H is the longitudinal diameter.

For the whole bladder infiltrating tumor, we approximate the bladder as an ellipsoid, and approximate the tumor volume as the bladder volume minus the bladder capacity, the formula is:

$$\text{Volume} = \frac{3\pi}{4} * L * S * H - \frac{3\pi}{4} * L' * S' * H',$$

$$\text{H or H}' = \text{number of layers} * \text{slice thickness},$$

Where L, S, H are the long, short, and longitudinal diameters of the bladder; L’, S’, H’ are the same for the bladder cavity.

Statistical analysis

Parametric tests (independent sample t-tests and ANOVA) and non-parametric tests (Wilcoxon rank-sum test, Mann-Whitney U test, Kruskal-Wallis test, and Fisher's exact test) were used for normally and non-normally distributed or categorical variables, respectively. The Pearson correlation test and Spearman correlation test were utilized to evaluate the correlation between normally and non-normally distributed variables, respectively. Log-rank test was applied to compare

survival differences between groups based on the Kaplan-Meier method. Statistical significance was considered at $P < 0.05$. Data visualizations and statistical analyses were conducted using R software (v4.2.1), Python software (v3.10) and ImageJ (v2) software. Binomial 95% confidence intervals were used to report all confidence intervals (CIs).

Additional References

1. Riester M, Werner L, Bellmunt J, Selvarajah S, Guancial EA, Weir BA, Stack EC, Park RS, O'Brien R, Schutz FA *et al*: **Integrative analysis of 1q23.3 copy-number gain in metastatic urothelial carcinoma**. *Clin Cancer Res* 2014, **20**(7):1873-1883.
2. Robertson AG, Groeneveld CS, Jordan B, Lin X, McLaughlin KA, Das A, Fall LA, Fantini D, Taxter TJ, Mogil LS *et al*: **Identification of Differential Tumor Subtypes of T1 Bladder Cancer**. *Eur Urol* 2020, **78**(4):533-537.
3. Choi W, Porten S, Kim S, Willis D, Plimack ER, Hoffman-Censits J, Roth B, Cheng T, Tran M, Lee IL *et al*: **Identification of distinct basal and luminal subtypes of muscle-invasive bladder cancer with different sensitivities to frontline chemotherapy**. *Cancer Cell* 2014, **25**(2):152-165.
4. Sjödahl G, Lauss M, Lövgren K, Chebil G, Gudjonsson S, Veerla S, Patschan O, Aine M, Fernö M, Ringnér M *et al*: **A molecular taxonomy for urothelial carcinoma**. *Clin Cancer Res* 2012, **18**(12):3377-3386.
5. Kim WJ, Kim EJ, Kim SK, Kim YJ, Ha YS, Jeong P, Kim MJ, Yun SJ, Lee KM, Moon SK *et al*: **Predictive value of progression-related gene classifier in primary non-muscle invasive bladder cancer**. *Mol Cancer* 2010, **9**:3.
6. Mariathasan S, Turley SJ, Nickles D, Castiglioni A, Yuen K, Wang Y, Kadel EE, III, Koeppen H, Astarita JL, Cubas R *et al*: **TGFβ attenuates tumour response to PD-L1 blockade by contributing to exclusion of T cells**. *Nature* 2018, **554**(7693):544-548.
7. Snyder A, Nathanson T, Funt SA, Ahuja A, Buros Novik J, Hellmann MD, Chang E, Aksoy BA, Al-Ahmadie H, Yusko E *et al*: **Contribution of systemic and somatic factors to clinical response and resistance to PD-L1 blockade in urothelial cancer: An exploratory multi-omic analysis**. *PLoS Med* 2017, **14**(5):e1002309.
8. Pusztai L, Yau C, Wolf DM, Han HS, Du L, Wallace AM, String-Reasor E, Boughey JC, Chien AJ, Elias AD *et al*: **Durvalumab with olaparib and paclitaxel for high-risk HER2-negative stage II/III breast cancer: Results from the adaptively randomized I-SPY2 trial**. *Cancer Cell* 2021, **39**(7):989-998.e985.
9. Wolf DM, Yau C, Wulfkuhle J, Brown-Swigart L, Gallagher RI, Lee PRE, Zhu Z, Magbanua MJ, Sayaman R, O'Grady N *et al*: **Redefining breast cancer subtypes to guide treatment prioritization and maximize response: Predictive biomarkers across 10 cancer therapies**. *Cancer Cell* 2022, **40**(6):609-623.e606.
10. Keenan TE, Guerriero JL, Barroso-Sousa R, Li T, O'Meara T, Giobbie-Hurder A, Tayob N, Hu J, Severgnini M, Agudo J *et al*: **Molecular correlates of response to eribulin and pembrolizumab in hormone receptor-positive metastatic breast cancer**. *Nat Commun* 2021, **12**(1):5563.
11. Choueiri TK, Fishman MN, Escudier B, McDermott DF, Drake CG, Kluger H, Stadler WM, Perez-Gracia JL, McNeel DG, Curti B *et al*: **Immunomodulatory Activity of Nivolumab in Metastatic Renal Cell Carcinoma**. *Clin Cancer Res* 2016, **22**(22):5461-5471.
12. Braun DA, Hou Y, Bakouny Z, Ficial M, Sant' Angelo M, Forman J, Ross-Macdonald P, Berger AC, Jegede OA, Elagina L *et al*: **Interplay of somatic alterations and immune infiltration modulates**

response to PD-1 blockade in advanced clear cell renal cell carcinoma. *Nat Med* 2020, **26**(6):909-918.

13. Motzer RJ, Rini BI, McDermott DF, Redman BG, Kuzel TM, Harrison MR, Vaishampayan UN, Drabkin HA, George S, Logan TF *et al*: **Nivolumab for Metastatic Renal Cell Carcinoma: Results of a Randomized Phase II Trial**. *J Clin Oncol* 2015, **33**(13):1430-1437.
14. Motzer RJ, Tannir NM, McDermott DF, Arén Frontera O, Melichar B, Choueiri TK, Plimack ER, Barthélémy P, Porta C, George S *et al*: **Nivolumab plus Ipilimumab versus Sunitinib in Advanced Renal-Cell Carcinoma**. *N Engl J Med* 2018, **378**(14):1277-1290.
15. Mahoney KM, Ross-Macdonald P, Yuan L, Song L, Veras E, Wind-Rotolo M, McDermott DF, Stephen Hodi F, Choueiri TK, Freeman GJ: **Soluble PD-L1 as an early marker of progressive disease on nivolumab**. *J Immunother Cancer* 2022, **10**(2).
16. Miao D, Margolis CA, Gao W, Voss MH, Li W, Martini DJ, Norton C, Bossé D, Wankowicz SM, Cullen D *et al*: **Genomic correlates of response to immune checkpoint therapies in clear cell renal cell carcinoma**. *Science* 2018, **359**(6377):801-806.
17. Ascierto ML, McMiller TL, Berger AE, Danilova L, Anders RA, Netto GJ, Xu H, Pritchard TS, Fan J, Cheadle C *et al*: **The Intratumoral Balance between Metabolic and Immunologic Gene Expression Is Associated with Anti-PD-1 Response in Patients with Renal Cell Carcinoma**. *Cancer Immunol Res* 2016, **4**(9):726-733.
18. Motzer RJ, Banchereau R, Hamidi H, Powles T, McDermott D, Atkins MB, Escudier B, Liu LF, Leng N, Abbas AR *et al*: **Molecular Subsets in Renal Cancer Determine Outcome to Checkpoint and Angiogenesis Blockade**. *Cancer Cell* 2020, **38**(6):803-817.e804.
19. Motzer RJ, Robbins PB, Powles T, Albiges L, Haanen JB, Larkin J, Mu XJ, Ching KA, Uemura M, Pal SK *et al*: **Avelumab plus axitinib versus sunitinib in advanced renal cell carcinoma: biomarker analysis of the phase 3 JAVELIN Renal 101 trial**. *Nat Med* 2020, **26**(11):1733-1741.
20. Liu S, Knochelmann HM, Lomeli SH, Hong A, Richardson M, Yang Z, Lim RJ, Wang Y, Dumitras C, Krysan K *et al*: **Response and recurrence correlates in individuals treated with neoadjuvant anti-PD-1 therapy for resectable oral cavity squamous cell carcinoma**. *Cell Rep Med* 2021, **2**(10):100411.
21. Phillips D, Matusiak M, Gutierrez BR, Bhate SS, Barlow GL, Jiang S, Demeter J, Smythe KS, Pierce RH, Fling SP *et al*: **Immune cell topography predicts response to PD-1 blockade in cutaneous T cell lymphoma**. *Nat Commun* 2021, **12**(1):6726.
22. van den Ende T, de Clercq NC, van Berge Henegouwen MI, Gisbertz SS, Geijsen ED, Verhoeven RHA, Meijer SL, Schokker S, Dings MPG, Bergman J *et al*: **Neoadjuvant Chemoradiotherapy Combined with Atezolizumab for Resectable Esophageal Adenocarcinoma: A Single-arm Phase II Feasibility Trial (PERFECT)**. *Clin Cancer Res* 2021, **27**(12):3351-3359.
23. Zhao J, Chen AX, Gartrell RD, Silverman AM, Aparicio L, Chu T, Bordbar D, Shan D, Samanamud J, Mahajan A *et al*: **Immune and genomic correlates of response to anti-PD-1 immunotherapy in glioblastoma**. *Nat Med* 2019, **25**(3):462-469.
24. Kim ST, Cristescu R, Bass AJ, Kim KM, Odegaard JI, Kim K, Liu XQ, Sher X, Jung H, Lee M *et al*: **Comprehensive molecular characterization of clinical responses to PD-1 inhibition in metastatic gastric cancer**. *Nat Med* 2018, **24**(9):1449-1458.
25. Obradovic A, Graves D, Korrer M, Wang Y, Roy S, Naveed A, Xu Y, Luginbuhl A, Curry J, Gibson M *et al*: **Immunostimulatory Cancer-Associated Fibroblast Subpopulations Can Predict Immunotherapy Response in Head and Neck Cancer**. *Clin Cancer Res* 2022, **28**(10):2094-2109.

26. Cho JW, Hong MH, Ha SJ, Kim YJ, Cho BC, Lee I, Kim HR: **Genome-wide identification of differentially methylated promoters and enhancers associated with response to anti-PD-1 therapy in non-small cell lung cancer**. *Exp Mol Med* 2020, **52**(9):1550-1563.
27. Jung H, Kim HS, Kim JY, Sun JM, Ahn JS, Ahn MJ, Park K, Esteller M, Lee SH, Choi JK: **DNA methylation loss promotes immune evasion of tumours with high mutation and copy number load**. *Nat Commun* 2019, **10**(1):4278.
28. Patil NS, Nabet BY, Müller S, Koeppen H, Zou W, Giltnane J, Au-Yeung A, Srivats S, Cheng JH, Takahashi C *et al*: **Intratumoral plasma cells predict outcomes to PD-L1 blockade in non-small cell lung cancer**. *Cancer Cell* 2022, **40**(3):289-300.e284.
29. Auslander N, Zhang G, Lee JS, Frederick DT, Miao B, Moll T, Tian T, Wei Z, Madan S, Sullivan RJ *et al*: **Robust prediction of response to immune checkpoint blockade therapy in metastatic melanoma**. *Nat Med* 2018, **24**(10):1545-1549.
30. Pomeranz Krummel DA, Nasti TH, Izar B, Press RH, Xu M, Lowder L, Kallay L, Rupji M, Rosen H, Su J *et al*: **Impact of Sequencing Radiation Therapy and Immune Checkpoint Inhibitors in the Treatment of Melanoma Brain Metastases**. *Int J Radiat Oncol Biol Phys* 2020, **108**(1):157-163.
31. Hugo W, Zaretsky JM, Sun L, Song C, Moreno BH, Hu-Lieskovan S, Berent-Maoz B, Pang J, Chmielowski B, Cherry G *et al*: **Genomic and Transcriptomic Features of Response to Anti-PD-1 Therapy in Metastatic Melanoma**. *Cell* 2016, **165**(1):35-44.
32. Riaz N, Havel JJ, Makarov V, Desrichard A, Urba WJ, Sims JS, Hodi FS, Martín-Algarra S, Mandal R, Sharfman WH *et al*: **Tumor and Microenvironment Evolution during Immunotherapy with Nivolumab**. *Cell* 2017, **171**(4):934-949.e916.
33. Liu D, Schilling B, Liu D, Sucker A, Livingstone E, Jerby-Arnon L, Zimmer L, Gutzmer R, Satzger I, Loquai C *et al*: **Integrative molecular and clinical modeling of clinical outcomes to PD1 blockade in patients with metastatic melanoma**. *Nat Med* 2019, **25**(12):1916-1927.
34. Gide TN, Quek C, Menzies AM, Tasker AT, Shang P, Holst J, Madore J, Lim SY, Velickovic R, Wongchenko M *et al*: **Distinct Immune Cell Populations Define Response to Anti-PD-1 Monotherapy and Anti-PD-1/Anti-CTLA-4 Combined Therapy**. *Cancer Cell* 2019, **35**(2):238-255.e236.
35. Chiappinelli KB, Strissel PL, Desrichard A, Li H, Henke C, Akman B, Hein A, Rote NS, Cope LM, Snyder A *et al*: **Inhibiting DNA Methylation Causes an Interferon Response in Cancer via dsRNA Including Endogenous Retroviruses**. *Cell* 2017, **169**(2):361.
36. Lauss M, Donia M, Harbst K, Andersen R, Mitra S, Rosengren F, Salim M, Vallon-Christersson J, Törngren T, Kvist A *et al*: **Mutational and putative neoantigen load predict clinical benefit of adoptive T cell therapy in melanoma**. *Nat Commun* 2017, **8**(1):1738.
37. Garcia-Diaz A, Shin DS, Moreno BH, Saco J, Escuin-Ordinas H, Rodriguez GA, Zaretsky JM, Sun L, Hugo W, Wang X *et al*: **Interferon Receptor Signaling Pathways Regulating PD-L1 and PD-L2 Expression**. *Cell Rep* 2017, **19**(6):1189-1201.
38. Kraehenbuehl L, Holland A, Armstrong E, O'Shea S, Mangarin L, Chekalil S, Johnston A, Bomalaski JS, Erinjeri JP, Barker CA *et al*: **Pilot Trial of Arginine Deprivation Plus Nivolumab and Ipilimumab in Patients with Metastatic Uveal Melanoma**. *Cancers (Basel)* 2022, **14**(11).
39. Salomé B, Sfakianos JP, Ranti D, Daza J, Bieber C, Charap A, Hammer C, Banchereau R, Farkas AM, Ruan DF *et al*: **NKG2A and HLA-E define an alternative immune checkpoint axis in bladder cancer**. *Cancer Cell* 2022, **40**(9):1027-1043.e1029.

40. Jiang P, Gu S, Pan D, Fu J, Sahu A, Hu X, Li Z, Traugh N, Bu X, Li B *et al*: **Signatures of T cell dysfunction and exclusion predict cancer immunotherapy response**. *Nat Med* 2018, **24**(10):1550-1558.
41. Wilkerson MD, Hayes DN: **ConsensusClusterPlus: a class discovery tool with confidence assessments and item tracking**. *Bioinformatics* 2010, **26**(12):1572-1573.
42. Brunet JP, Tamayo P, Golub TR, Mesirov JP: **Metagenes and molecular pattern discovery using matrix factorization**. *Proc Natl Acad Sci U S A* 2004, **101**(12):4164-4169.
43. Subramanian A, Tamayo P, Mootha VK, Mukherjee S, Ebert BL, Gillette MA, Paulovich A, Pomeroy SL, Golub TR, Lander ES *et al*: **Gene set enrichment analysis: a knowledge-based approach for interpreting genome-wide expression profiles**. *Proc Natl Acad Sci U S A* 2005, **102**(43):15545-15550.
44. Szklarczyk D, Kirsch R, Koutrouli M, Nastou K, Mehryary F, Hachilif R, Gable AL, Fang T, Doncheva NT, Pyysalo S *et al*: **The STRING database in 2023: protein-protein association networks and functional enrichment analyses for any sequenced genome of interest**. *Nucleic Acids Res* 2023, **51**(D1):D638-d646.
45. Hao Y, Hao S, Andersen-Nissen E, Mauck WM, 3rd, Zheng S, Butler A, Lee MJ, Wilk AJ, Darby C, Zager M *et al*: **Integrated analysis of multimodal single-cell data**. *Cell* 2021, **184**(13):3573-3587.e3529.
46. Aran D, Looney AP, Liu L, Wu E, Fong V, Hsu A, Chak S, Naikawadi RP, Wolters PJ, Abate AR *et al*: **Reference-based analysis of lung single-cell sequencing reveals a transitional profibrotic macrophage**. *Nat Immunol* 2019, **20**(2):163-172.
47. Qiu X, Mao Q, Tang Y, Wang L, Chawla R, Pliner HA, Trapnell C: **Reversed graph embedding resolves complex single-cell trajectories**. *Nat Methods* 2017, **14**(10):979-982.
48. Zou J, Deng F, Wang M, Zhang Z, Liu Z, Zhang X, Hua R, Chen K, Zou X, Hao J: **scCODE: an R package for data-specific differentially expressed gene detection on single-cell RNA-sequencing data**. *Brief Bioinform* 2022, **23**(5).
49. Şenbabaoğlu Y, Michailidis G, Li JZ: **Critical limitations of consensus clustering in class discovery**. *Sci Rep* 2014, **4**:6207.
50. Hänzelmann S, Castelo R, Guinney J: **GSVA: gene set variation analysis for microarray and RNA-seq data**. *BMC Bioinformatics* 2013, **14**:7.
51. Ritchie ME, Phipson B, Wu D, Hu Y, Law CW, Shi W, Smyth GK: **limma powers differential expression analyses for RNA-sequencing and microarray studies**. *Nucleic Acids Res* 2015, **43**(7):e47.
52. Yu G, Wang LG, Han Y, He QY: **clusterProfiler: an R package for comparing biological themes among gene clusters**. *Omics* 2012, **16**(5):284-287.
53. Mayakonda A, Lin DC, Assenov Y, Plass C, Koeffler HP: **Maftools: efficient and comprehensive analysis of somatic variants in cancer**. *Genome Res* 2018, **28**(11):1747-1756.
54. Charoentong P, Finotello F, Angelova M, Mayer C, Efremova M, Rieder D, Hackl H, Trajanoski Z: **Pan-cancer Immunogenomic Analyses Reveal Genotype-Immunophenotype Relationships and Predictors of Response to Checkpoint Blockade**. *Cell Rep* 2017, **18**(1):248-262.
55. Şenbabaoğlu Y, Gejman RS, Winer AG, Liu M, Van Allen EM, de Velasco G, Miao D, Ostrovnaya I, Drill E, Luna A *et al*: **Tumor immune microenvironment characterization in clear cell renal cell carcinoma identifies prognostic and immunotherapeutically relevant messenger RNA signatures**. *Genome Biol* 2016, **17**(1):231.

56. Yoshihara K, Shahmoradgoli M, Martínez E, Vegesna R, Kim H, Torres-Garcia W, Treviño V, Shen H, Laird PW, Levine DA *et al*: **Inferring tumour purity and stromal and immune cell admixture from expression data**. *Nat Commun* 2013, **4**:2612.
57. Dong M, Thennavan A, Urrutia E, Li Y, Perou CM, Zou F, Jiang Y: **SCDC: bulk gene expression deconvolution by multiple single-cell RNA sequencing references**. *Brief Bioinform* 2021, **22**(1):416-427.
58. Hoshida Y, Brunet JP, Tamayo P, Golub TR, Mesirov JP: **Subclass mapping: identifying common subtypes in independent disease data sets**. *PLoS One* 2007, **2**(11):e1195.
59. Maeser D, Gruener RF, Huang RS: **oncoPredict: an R package for predicting in vivo or cancer patient drug response and biomarkers from cell line screening data**. *Brief Bioinform* 2021, **22**(6).
60. Yang W, Soares J, Greninger P, Edelman EJ, Lightfoot H, Forbes S, Bindal N, Beare D, Smith JA, Thompson IR *et al*: **Genomics of Drug Sensitivity in Cancer (GDSC): a resource for therapeutic biomarker discovery in cancer cells**. *Nucleic Acids Res* 2013, **41**(Database issue):D955-961.
61. Rees MG, Seashore-Ludlow B, Cheah JH, Adams DJ, Price EV, Gill S, Javaid S, Coletti ME, Jones VL, Bodycombe NE *et al*: **Correlating chemical sensitivity and basal gene expression reveals mechanism of action**. *Nat Chem Biol* 2016, **12**(2):109-116.
